# Acid-Induced Rearrangement of Epoxygermacranolides: Synthesis of Furanoheliangolides and Cadinanes from Nobilin

**DOI:** 10.3390/molecules22122252

**Published:** 2017-12-18

**Authors:** Maria De Mieri, Martin Smieško, Isidor Ismajili, Marcel Kaiser, Matthias Hamburger

**Affiliations:** 1 Pharmaceutical Biology, Pharmazentrum, University of Basel, Klingelbergstrasse 50, 4056 Basel, Switzerland; isidor.ismajili@gmail.com; 2 Molecular Modeling, Pharmazentrum, University of Basel, Klingelbergstrasse 50, 4056 Basel, Switzerland; martin.smiesko@unibas.ch; 3 Department of Medical Parasitology & Infection Biology, Swiss Tropical and Public Health Institute, Socinstrasse 57, 4000 Basel, Switzerland; marcel.kaiser@swisstph.ch; 4 University of Basel, Petersplatz 1, 4001 Basel, Switzerland

**Keywords:** sesquiterpene lactones, epoxygermacranolides, acid-catalyzed rearrangement, mechanism of reaction, anti-protozoal activity

## Abstract

The acid-induced rearrangement of three epoxyderivatives of nobilin **1**, the most abundant sesquiterpene lactone in *Anthemis* *nobilis* flowers, was investigated. From the 1,10-epoxyderivative **2**, furanoheliangolide **5** was obtained, while the 4,5-epoxy group of **3** did not react. Conversely, when the 3-hydroxy function of nobilin was acetylated (**12**), the 4,5-epoxy derivative did cyclize into cadinanes (**15** and **16**) under Lewis acid catalysis. The reactivity of the 4,5- and 1,10-epoxy derivatives of nobilin (**2** and **3**) was compared with that of parthenolide, and rationalized on the basis of quantum chemical calculations. All isolated reaction products were fully characterized by spectroscopic and computational methods, and their in vitro anti-protozoal activity was evaluated. The paper could provide new insights into the biosynthesis of this class of natural products.

## 1. Introduction

Sesquiterpene lactones are the largest group of secondary metabolites, comprising more than 10,000 structures known up to now. They possess high structural diversity and are chemotaxonomic markers of the *Asteraceae* and *Compositae* families [1]. Germacrene-type sesquiterpenoids possess a cyclodecadiene ring in their scaffold, and are considered key precursors in the biosynthesis of other natural sesquiterpenes. The two endocyclic double bonds, found in four possible geometrical isomers, are responsible for the high reactivity of the cyclodecadiene moiety, which undergoes cyclization even under mild conditions [2]. Therefore, germacrenes are ideal starting points for diversity-oriented and biomimetic synthesis [3,4,5,6]. It is well established that the outcome of the cyclization depends on the spatial rearrangement of the cyclodecadiene ring and, therefore, on the stereochemistry of the diene. We recently reported on the spontaneous rearrangement of nobilin **1**, an *E*,*Z*-germacranolide isolated from the flowers of Roman chamomile (*Anthemis nobilis*) in aqueous media under physiological conditions [7]. We then investigated the rearrangement of **1** in aqueous and organic acidic media and found a transformation into various cadinane derivatives [8]. In the present study we investigated the rearrangement of epoxy derivatives **2**–**4** of nobilin (**1**) under acidic conditions. We hypothesized that the presence of an epoxy group, acting as a conformational anchor, would strongly affect the outcome of the cyclization [9]. This has previously been shown for parthenolide, the major sesquiterpene lactone in feverfew (*Parthenium integrifolium*), which undergoes transannular cyclization to a guianolide skeleton [10,11,12]. We here compare the reactivity of nobilin (**1**) and of its epoxy derivatives **2** and **3** with that of parthenolide, and rationalize the outcome on the basis of conformational analysis and computational studies.

## 2. Results and Discussion

### 2.1. Chemical Studies

**Scheme 1:**
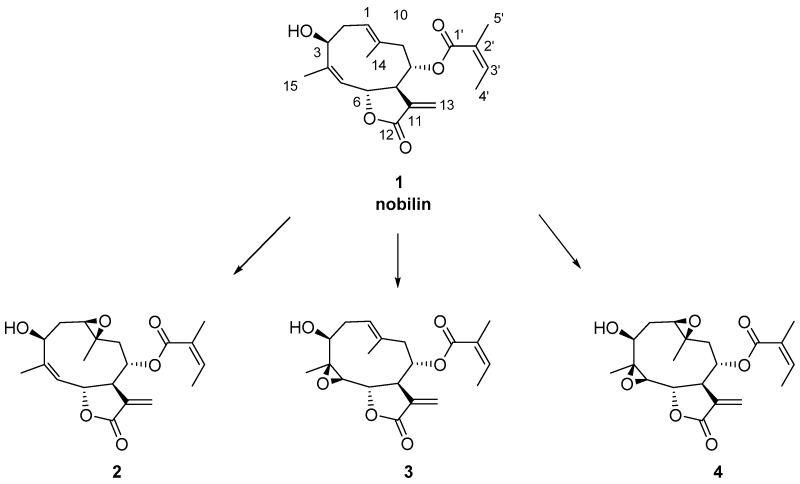
Structures of nobilin **1** and epoxy derivatives **2**–**4**. The carbon numbering follows the semi-systematic nomenclature of sesquiterpenes.

Nobilin **1** was isolated in large quantities from the *Anthemis nobilis* flower cones. Epoxides **2** and **3** were prepared according to reported procedures [13]. Treatment of nobilin **1** with *meta*-chloroperoxybenzoic acid (*m*-CPBA) in dichlomethane afforded compounds **2**–**4** in a 10:1:0.5 ratio. Epoxide **2** showed identical spectroscopic data to those reported for the natural compound [14]. Epoxide **3** was here obtained for the first time by the Sharpless epoxidation method (Scheme 1) [15]. The *beta* stereochemistry of the 4,5-epoxy group of **3** was inferred via *J* couplings and NOEs. The ^3^*J* H-5/H-6 value of 9.7 Hz was indicative of the anti-diaxial orientation of H-5 and H-6, and key NOEs between H-6/H-8 and H-6/H_3_-14 supported the cofacial orientation of H-6, H-8, and H_3_-14. Similarly, key NOEs between H-5/H-7, H-5/H_3_-15, and H-1/H_3_-15 showed that H-7, H_3_-15, and H-1 were on the same molecular plane, in agreement with the optimized structure of **3**.

**Scheme 2:**
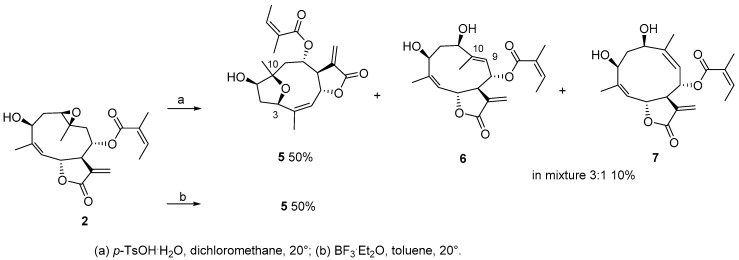
Reactivity of **2** with acids.

We began our study by treating compound **2** with *p*-toluensulfonic acid (*p*-TsOH·H_2_O), a mild acid that has been extensively used to induce transannular cyclization in germacradiene derivatives [8,16]. After 1 h at room temperature, compound **5** (50%) and an inseparable mixture of **6** and **7** in a 3:2 ratio (10%) were obtained (Scheme 2). Compound **5** had the same molecular formula and degree of unsaturation as **2** (molecular ion at *m*/*z* 385.1632 [M + Na]^+^, calculated for C_20_H_26_NaO_6_: 385.1622) by analysis of its ^13^C-NMR and HRESIMS spectra. Analysis of COSY and HMBC spectra revealed that the fragment C4–C9 of **5** was as in the starting material. A significant downfield shift for C-1 and C-10 (δ_C_ = 77.6, C-1; δ_C_ = 87.3, C-10) compared to those in **2** (δ_C_ = 60.7, C-1; δ_C_ = 57.7, C-10) suggested that a rearrangement of the epoxy group has occurred, although both carbons still bore hydroxyl groups. The downfield shift of C-3 (δ_C_ = 81.7 in **5** vs. δ_C_ = 71.2 in **2**), along with the long-range HMBC correlation from H-3 to C-10, suggested the presence of an ether bridge between carbons C-3 and C-10. Hence, **5** was a furanoheliangolide. The beta stereochemistry of the epoxy bridge, inferred on the basis of geometrical and mechanistic considerations, was confirmed through key NOEs between H-1/H-3, H-3/H-6, H_3_-14/H-8, and H_3_-14/H-6 that were in agreement with the 3D structure of **5** (Figures S22 and S23).

Compounds **6** and **7** were obtained as an inseparable mixture in a ratio of 3:1. For both compounds, a [M + Na]^+^ ion at *m*/*z* 385.1632 was observed, corresponding to a molecular formula of C_20_H_26_NaO_6_ (calculated for C_20_H_26_NaO_6_: 385.1622). Hence, the two molecules were isomers. Close inspection of the NMR data (Table 1 and Table 2) revealed that in both compounds the methylene group at C-9 had been replaced by vinylic moieties (δ_H_ = 5.18 and 5.30 for **6** and **7**, respectively), thereby suggesting that the opening of the oxirane and subsequent formation of an allylic carbon had occurred. The differing chemical shifts of the vinylic protons were attributed to the *E*- and *Z*-stereochemistry of the newly formed double bond, respectively. The strong NOESY contact between H-9 and H-1 (δ_H_ = 5.18 and 4.05, respectively), and between H_3_-14 and H-6 (δ_H_ = 1.95 and 6.00, respectively), in the major isomer **6** of the mixture were in favor of a *trans*-oriented double bond. For the minor compound **7** of the mixture, a strong NOESY cross peak between H-9 and H_3_-14 (δ_H_ = 5.30 and 1.80, respectively) supported the *cis* configuration of the double bond at C-9/C-10 (Figure S28). Interestingly, the C-1 epimer of compound **7** has recently been isolated from *Anthemis nobilis* [17].

A similar rearrangement to a furanoheliangolide was observed after treatment of **2** with BF_3_·Et_2_O in anhydrous conditions, and **5** was obtained as the major reaction product (50%) (Scheme 2). Surprisingly, epoxy derivative **3** did not react under the above reaction conditions, as confirmed by TLC and NMR (Figure S63).

**Scheme 3:**
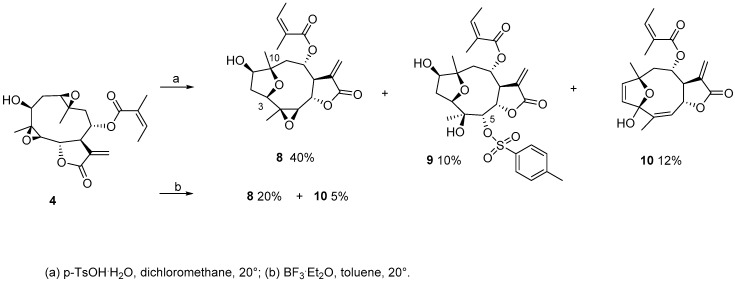
Reactivity of **4** with acids.

For further confirmation of the higher reactivity of the 1,10-epoxyde compared to the 4,5-epoxide, the bisepoxy derivative **4** was submitted to the same acidic treatment (Scheme 3). Similar to **2**, treatment of **4** afforded C-3-C-10 furanoheliangolide derivatives **8**–**10**. In compound **8** (HRESIMS *m*/*z* 401.1591 [M + Na]^+^, calculated for C_20_H_26_NaO_7_ 401.1571), which was obtained as a major reaction product by treatment with both acid catalysts, the 4,5-epoxy group did not react. Conversely, the 4,5-epoxide underwent nucleophilic addition of the catalyst and elimination in compounds **9** and **10**, respectively. 

Compound **9**, showed a molecular formula of C_27_H_34_O_10_S (HRESIMS *m*/*z* 573.1786 [M + Na]^+^, calculated for C_27_H_34_NaO_10_S: 573.1765). In the ^1^H-NMR spectrum, resonances characteristic of an AA′BB′ aromatic system were present (δ_H_ = 7.41 and 7.76 pseudo d, *J* = 8.3 Hz, 2 protons each), together with an additional methyl group attached to an aromatic ring (δ_H_ = 2.45, s, 3H). These spectroscopic data, together with the sulphonyl group indicated by the molecular formula, showed that the *p*-toluensulphonate group has reacted with **4**. The site of the nucleophilic attach was located at C-5, as indicated by the downfield shift of H-5 (δ_H_ = 4.79 and δ_C_ = 89.3 in **9** vs. δ_H_ = 2.85 and δ_C_ = 63.4 in **4**), while its orientation was established as *alpha* on the basis of the ^3^*J*H-5/H-6 value of ≤1 Hz.

Compound **10** showed a molecular formula of C_20_H_24_NaO_6_ (HRESIMS *m*/*z* 383.1500 [M + Na]^+^, calculated for C_20_H_24_NaO_6_: 383.1465) with nine degrees of hydrogen deficiency, which, compared to **4**, indicated the loss of an oxygen group and the formation of an additional double bond. The NMR data revealed diagnostic differences in comparison to **4**, including three olefinic protons in the low field region, two of which showed ^3^*J* coupling (δ_H_ = 5.48 dq, *J* = 5.8 and 1.2 Hz, δ_H_ = 5.89 d, *J* = 5.7 Hz, and δ_H_ = 6.22 d, *J* = 5.7 Hz), absence of oxygenated protons at C-1, C-3 and C-5, absence of the methylene group attached at C-2, and the presence of a quaternary carbon resonating at δ_C_ = 110.4, diagnostic of a hemiacetal group. The latter carbon resonance exhibited HMBC correlations with the ^3^*J* coupled olefin and the olefinic methyl group. All this spectroscopic evidence suggested that a dihydrofurane had been formed via elimination of the hydroxyl group at C-1, and that the 4(5)-epoxide had rearranged into an allylic hemiacetal. A speculative mechanism for the latter transformation would start with the opening of the 4,5-epoxy group at C-4, subsequent formation of an allylic alcohol (double bond between C-3 and C-4 and hydroxyl group at C-5), followed by transposition of the hydroxyl group to C-3 and of the double bond between C-4 and C-5 [18,19]. The *cis* stereochemistry of the C-4/C-5 double bond was assigned via the key NOE between H-5 and H_3_-15. The 8-epimer of compound **10** has been previously isolated as a natural product from the sunflower cultivar cv. Peredovick [20].

Given that the postulated reactivity of the 1(10)-epoxy group towards a transannular cyclization was precluded by the high reactivity of the hydroxyl group at C-3, we acetylated this moiety of epoxy derivatives **2** and **3** in order to prevent the formation of the furane ring and to facilitate a transannular cyclization. 

**Scheme 4:**
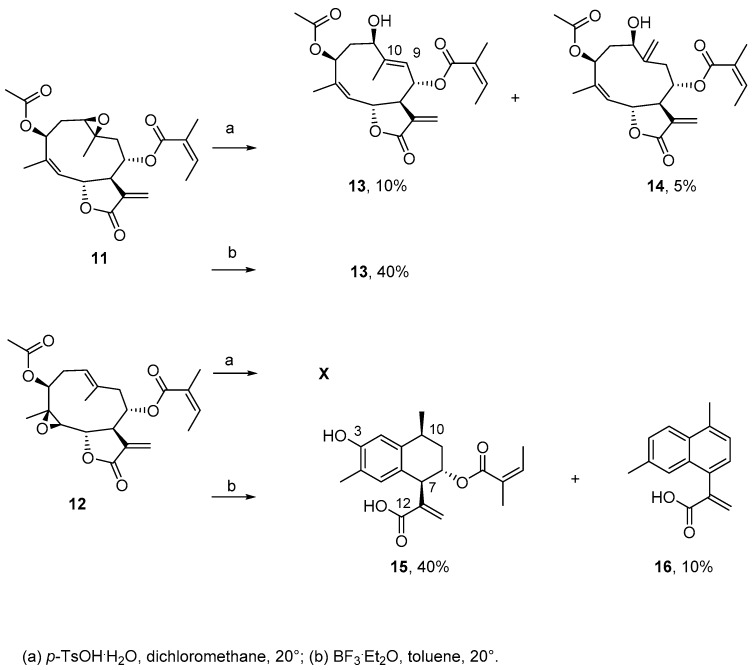
Reactivity of **11** and **12** with acids.

The reaction of **11** with *p*-TsOH·H_2_O proceeded slowly and afforded compounds **13** and **14** in 10% and 5% yield, respectively (Scheme 4). Compound **13** (HRESIMS *m*/*z* 427.1749 [M + Na]^+^, calculated for C_22_H_28_NaO_7_: 427.1727) showed NMR data virtually superimposable to those of **6**, with the difference of an additional 3-acetoxy moiety. The compound was isolated in higher yield (40%) using BF_3_·Et_2_O as acid catalyst.

Compound **14** showed the same molecular formula as **13** and was a positional isomer of the latter. Compared to **13**, the NMR spectra indicated the absence of a methyl group at C-10 and of an olefinic proton at C-9, together with the appearance of an exocyclic methylene group (δ_H_ = 5.48 and 5.51, br s, δ_C_ = 116.5). Hence, the position of the unsaturation was at C-10/C-14.

As for **3**, derivative **12** was recovered unchanged after stirring at room temperature for 12 h in the presence of *p*-TsOH·H_2_O. Conversely, treatment of **12** with BF_3_·Et_2_O yielded cadinane acids **15** and **16**. Compound **15** showed a molecular formula of C_20_H_24_O_5_ (HRESIMS *m*/*z* 367.1530 [M + Na]^+^; calculated for C_20_H_24_NaO_5_: 367.1516). Close inspection of the NMR data revealed that **15** was the 3-hydroxy derivative of a cadinane that had been previously obtained by a reaction of nobilin with acids, whereby the transannular cyclization of the 1(10) double bond was facilitated by the alkyl–oxygen cleavage of the lactone ring. The reaction led to the formation of a single bond, C-5/C-10, which transformed the cyclodecadiene into a decaline ring system [7,8].

Compound **16** had a molecular formula of C_15_H_14_O_2_ (HRESIMS *m*/*z* 249.0899 [M + Na]^+^, calculated for C_15_H_14_NaO_2_: 249.0886). Its ^1^H-NMR spectrum displayed resonances of seven olefinic protons and two methyl groups (Table 3 and Table 4), indicative of the loss of the angelate and hydroxyl substituents, and the formation of a naphthalene ring bearing the acrylate group (Scheme 4).

### 2.2. Computational Studies

**Scheme 5:**
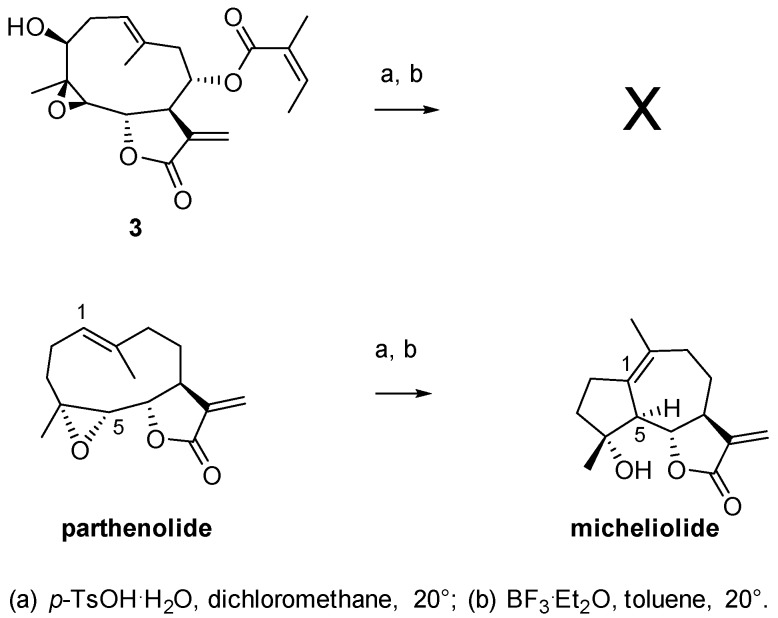
Reactivity of **3** and parthenolide with acids.

**Figure 1:**
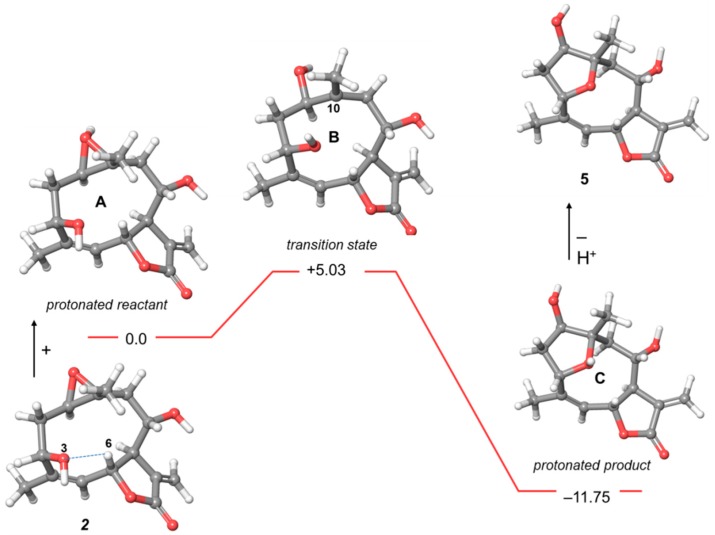
Energy profile of the transformation of **2** into **5**. Relative energies are reported in kcal/mol. The hydrogen bonding interaction O-3…H-6 is represented with a dashed line.

**Figure 2:**
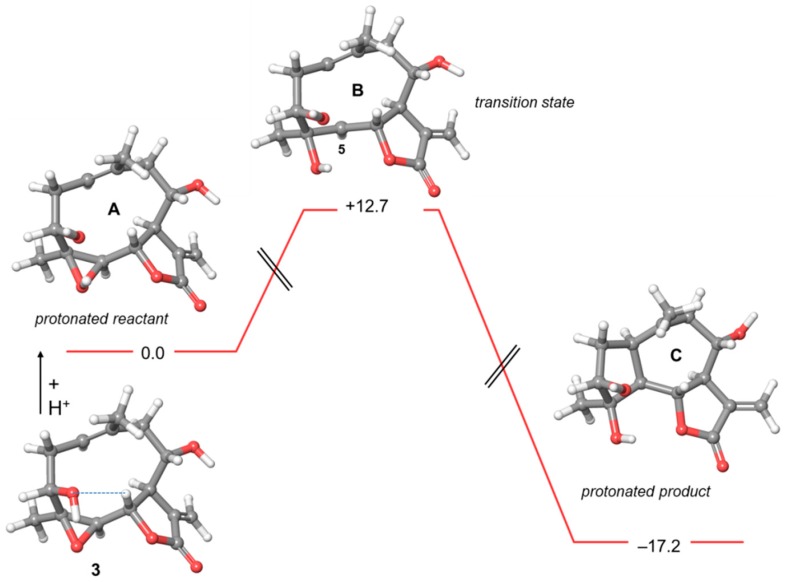
Energy profile of the putative transannular cyclization of **3**. Relative energies are reported in kcal/mol. The hydrogen bonding interaction O-3…H-6 is represented with a dashed line.

**Figure 3:**
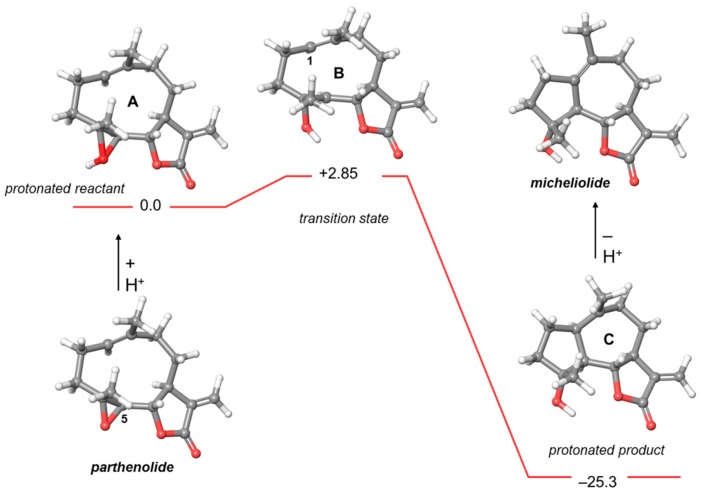
Energy profile of the transformation of parthenolide into micheliolide. Relative energies are reported in kcal/mol.

In order to rationalize the differing reactivity of epoxides **2** and **3** (Figure 1 and Figure 2, respectively) and compare it with that of parthenolide (Scheme 5), we performed theoretical studies. The aim was to characterize, at the molecular level, transition states of intermediates and products and to predict reaction diagrams. Furthermore, we postulated the conversion of **3** into its corresponding micheliolide derivative following the same reaction pathway as reported for parthenolide Figure 3.

Prior to reaction calculations, an exhaustive conformational analysis of **2**, **3**, and parthenolide was carried out, followed by optimization of the most stable conformer by density functional calculation at the B3LYP/6-31+G(d,p) level [21]. Calculations were performed using the SMD solvation model [22] with dichloromethane as a solvent to mimic the reaction conditions (a). For compounds **2** and **3**, the angelate ester moiety was omitted since it had no influence on the conformation and, therefore, on the reactivity of the germacranolide ring, as previously shown [23]. 

The global minimum structure of **2** (Figure 1) was stabilized by a non-conventional intramolecular hydrogen bond between O-3 and H-6 (distance O3-H6 2.23 Å). Upon treatment with acid, the epoxy group was protonated leading to the protonated reactant **A**. The intrinsic reaction coordinate of the transition state **B** involved the formation of a carbocation at C-10 requiring an activation energy of 5.03 kcal/mol. The occurrence of a transannular cyclization for **B** was precluded by the long distances of C10–C5 and C10–C4 (4.05 and 3.68 Å, respectively) due to the non-conventional intramolecular hydrogen bond between O-3 and H-6 (2.08 Å). Conversely, the close distance of O3–C1 (2.83 Å) enabled the formation of the protonated product **C**.

The reaction diagram of the conversion of **3** into **C**, a congener of micheliolide is shown in Figure 2, with the guaianolide scaffold resulting from the transannular cyclization. As in case of **2**, the energetically optimized conformation of **3** was stabilized by a non-conventional intramolecular hydrogen bond between O-3 and H-6 (2.33 Å). In acidic media, **3** would lead to the protonated epoxide **A**. However, evolution of **3** into transition state **B** bearing a C-5 carbocation would be prevented by the too high energy barrier of 12.7 kcal/mol.

Theoretical studies on the conversion of parthenolide into micheliolide (Figure 3) showed that the formation of the transition state **B** bearing the carbocation at C-5 was energetically more favored. Furthermore, key distances of C1–C5 (3.03 and 2.61 Å in **A** and **B**, respectively) allowed a transannular cyclization of the C1(10) double bond into the new bond C1–C5.

### 2.3. In Vitro Anti-Protozoal Activity

Considering the antiprotozoal activity of other sesquiterpene lactones [24,25], compounds **1**, **3**–**8**, and **10**–**15** were tested for their in vitro activity against *Trypanosoma brucei rhodesiense*, *T. cruzi*, *Leishmania donovani*, and *Plasmodium falciparum* (Table 5)*.* In parallel, their cytotoxicity in L6 cells was evaluated in order to obtain a first appreciation of the selectivity. The tested compounds exhibited anti-protozoal activity in the micromolar range. Among the tested parasites, *T. b. rhodesiense* was generally found to be the most sensitive (IC_50_ 0.47–13.4 µM, Selectivity Index (SI) 0.3–4). Epoxides **3**–**4** were as active as nobilin (**1**) against *T. b. rhodesiense*. The anti-trypanosomal activity of furanoheliangolides **5**, **8**, and **10** varied depending on the substitution patterns. Decoration of the cyclodecadiene skeleton with more polar functional groups, e.g., a hydroxyl group as in **6** and **7** caused a doubling of activity. Acetylation of the alcoholic function at C-3 increased activity as observed in compounds **11**–**14** (IC_50_ 0.47–1.57 µM). However, all compounds showed significant cytotoxicity in L6 cells.

## 3. Experimental Section

### 3.1. General Information

Optical rotation was measured in MeOH (1 mg/mL) with a P-2000 digital polarimeter (Jasco, Tokyo, Japan) using a 10-cm microcell. UV and CD spectra were recorded in MeOH (120 μg/mL) on a Chirascan CD spectrometer, and data analyzed with Pro-Data V2.4 software. NMR spectra were recorded on a Bruker Avance III spectrometer operating at 500.13 MHz for ^1^H, and 125.77 MHz for ^13^C. ^1^H-NMR, COSY, HSQC, HMBC, and NOESY spectra were measured at 18 °C in a 1 mm TXI probe with a z-gradient, using standard Bruker pulse sequences. ^13^C-NMR/DEPTQ spectra were recorded at 23 °C in a 5 mm BBO probe with a z-gradient. Spectra were analyzed by Bruker TopSpin 3.0 (Bruker Biospin, Fällende, Zurich, Switzerland). The following abbreviations are used to describe the signal multiplicities: s (singlet), d (doublet), t (triplet), q (quartet,) and m (multiplet).

High-resolution mass spectra (ESI-TOF-MS) were recorded in positive mode on a Bruker microTOF ESIMS system. Nitrogen was used as a nebulizing gas at a pressure of 2.0 bar, and as drying gas at a flow rate of 9.0 L/min (dry gas temperature 240 °C). Capillary voltage was set at 45,000 V, and the hexapole at 230.0 Vpp. Instrument calibration was done with a reference solution of 0.1% sodium formiate in 2-propanol/water (1:1) containing 5 mM NaOH.

HPLC-PDA-ESIMS spectra were obtained in positive mode on a Bruker Daltonics Esquire 3000 Plus ion trap MS system, connected via T-splitter (1:10) to an Agilent HP 1100 system consisting of degasser, binary mixing pump, autosampler, column oven, and a diode array detector (G1315B). Data acquisition and processing was performed with Hystar 3.0 software (Bruker Optics, Fällende, Zurich, Switzerland). Semipreparative HPLC separations were carried out with an Agilent HP 1100 series system consisting of a quaternary pump, autosampler, column oven, and diode array detector (G1315B). Waters SunFire™ (Petaluma, CA, USA) (C18, 3.5 μm, 3.0 × 150 mm i.d.) and SunFire™ Prep C18 (5 μm, 10 × 150 mm i.d) columns were used for analytical and semi-preparative separations, respectively. HPLC-grade MeOH, acetonitrile, (Scharlau Chemie S.A., Barcelona, Spain) and water were used for HPLC separations. HPLC solvents contained 0.1% of HCO_2_H for analytical and semi-preparative separations. Dry toluene was obtained by passing commercially available analytical grade toluene (Scharlau Chemie S.A, Barcelona, Spain) through an activated alumina column. NMR spectra were recorded in methanol-*d*_4_ (Armar Chemicals). Technical-grade solvents purified by distillation were used for extraction and open column chromatography. Silica gel (63–200 μm and 15–40 μm, Merck, Kenilworth, NJ, USA) was used for open-column chromatography.

### 3.2. In Vitro Biological Testing

Compounds were dissolved in DMSO (10 mg/mL) and stored at −20 °C until testing. Fresh dilutions in medium were prepared for each bioassay. The DMSO concentration in the assay did not exceed 1%. All assays were performed in at least three independent experiments. The purity of all compounds was >95% unless stated otherwise. 

The in vitro activities against the protozoan parasites *T. b. rhodesiense* (STIB900) bloodstream forms, *T. cruzi* (Tulahuen C4 LacZ) intracellular amastigotes, *L. donovani* (MHOM-ET-67/L82) axenically grown amastigotes, and *P. falciparum* (NF54) erythrocytic stage and cytotoxicity in L6 cells (rat skeletal myoblasts) were determined as reported elsewhere [26].

### 3.3. Isolation of Nobilin *(**1**)*

Nobilin (**1**) was isolated from flower cones of *Anthemis nobilis* (Dixa AG, St. Gallen, Switzerland). Flowers were milled and percolated with *n*-hexane (2 × 5 L), followed by CH_2_Cl_2_ (2 × 5 L). The CH_2_Cl_2_ extract was dried and separated by CC on SiO_2_ (400–600 mesh), using a gradient of *n*-hexane/EtOAc (90:10 to 50:50) as mobile phase. Nobilin-enriched fractions were combined and purified by CC on SiO_2_ (400–150 mesh). A gradient of *n*-hexane/EtOAc 95:5 to 60:40 was used as mobile phase. After recrystallization from EtOAc/*n*-hexane, 800 mg of white solid were obtained. Spectroscopic data were identical to those reported in the literature. *R*_f_ = 0.80 (SiO_2_, *n*-hexane/EtOAc 60:40). ${\left[\alpha \right]}_{D}^{25}$ = −21.67 (*c* = 0.44, CH_3_OH). ^1^H-NMR (CD_3_OD, 500 MHz): δ 1.74 (d, *J* = 1.4 Hz, 3H, 15-CH_3_), 1.87 (quint, *J* = 1.5 Hz, 3H, 5′-CH_3_), 1.89 (br s, 3H, 14-CH_3_), 1.93 (dq, *J* = 7.2 and 1.5 Hz, 3H, 4′-CH_3_), 2.23 (ddd, *J* = 13.8, 7.2 and 3.5 Hz, 1H, 2-H_b_), 2.46 (dd, *J* = 12.7 and 10.5 Hz, 1H, 9-H_b_), 2.50–2.68 (mov, 2H, 2-H_a_ and 9-H_a_), 3.05 (dq, *J* = 10.0, and 1.8 Hz, 1H, 7-H), 4.39 (dd, *J* = 3.5 and 2.8 Hz, 1H, 3-H), 5.03 (ddd, *J* = 10.5, 10.0 and 3.7 Hz, 1H, 8-H), 5.17 (dq, *J* = 10.4 and 1.4 Hz, 1H, 5-H) 5.37 (dd, *J* = 9.3 and 7.2 Hz, 1H, 1-H), 5.70 (br s, 1H, 13-H_b_), 6.05 (dd, *J* = 10.4 and 1.8 Hz, 1H, 6-H), 6.11 (qq, *J* = 7.2 and 1.5 Hz, 1H, 3′-H), 6.20 (br s, 1H, 13-H_a_) ppm. ^13^C-NMR (CD_3_OD, 125 MHz): δ 16.1 (CH_3_, 4′-C), 18.6 (CH_3_, 14-C), 20.6 (CH_3_, 5′-C), 23.6 (CH_3_, 15-C), 32.5 (CH_2_, 2-C), 49.0 (CH_2_, 9-C), 52.2 (CH, 7-C), 71.3 (CH, 8-C), 77.4 (CH, 3-C), 79.4 (CH, 6-C), 126.2 (CH, 5-C), 127.0 (CH, 1-C), 127.3 (CH_2_, 13-C), 128.9 (C, 2′-C), 134.6 (C, 10-C), 137.3 (C, 11-C), 139.9 (CH, 3′-C), 142.4 (C, 4-C), 168.3 (C, 1′-C), 172.4 (C, 12-C) ppm. HR-ESIMS calculated for C_20_H_26_O_5_Na [M + Na]^+^, 369.1672; found, 369.1672. The CD spectrum (Figure S62) was in agreement with the literature [14].

### 3.4. Synthesis of Compounds ***2*** and ***4***

To a stirred solution of **1** (200 mg; 0.58 mmol) in DCM (10 mL), a solution of mCPBA (77%; 207 mg; 0.93 mmol) in DCM (1 mL) was added under cooling with ice. The reaction mixture was then allowed to stand at room temperature for 4 h. On the basis of a TLC analysis, the reaction was quenched by the addition of 10% Na_2_S_2_O_3_ (2 × 5 mL). The absence of peroxides was tested with KI paper. The organic phase was then dried under Na_2_S_2_O_4_ and the solvent removed under reduced pressure. The obtained residue was purified by chromatography (SiO_2_; *n*-hexane/AcOEt) to afford **2** (140 mg; 0.39 mmol; 70%), **3** (10 mg; 0.028 mmol; 7%), and **4** (9 mg; 0.024 mmol; 4%) as white solids. 

*(−)-(1R,10R)-1,10-Epoxynobilin* (**2**) *R*_f_ = 0.5 (SiO_2_, hexane/EtOAc 60:40). ${\left[\alpha \right]}_{D}^{25}$ = −16.9 (*c* = 0.1, CH_3_OH). ^1^H-NMR (CD_3_OD, 500 MHz): δ 1.48 (dd, *J* = 13.0, and 11.5 Hz, 1H, 9-H_ax_), 1.53 (s, 3H, 14-CH_3_), 1.63 (ddd, *J* = 14.5, 10.2 and 2.6 Hz, 1H, 2-H_ax_), 1.80 (br s, 3H, 15-CH_3_), 1.85 (quint, *J* = 1.5 Hz, 3H, 5′-CH_3_), 1.92 (dq, *J* = 7.0 and 1.5 Hz, 3H, 4′-CH_3_), 2.48 (ddd, *J* = 14.5, 4.5 and 4.3 Hz, 1H, 2-H_eq_), 2.50 (dd, *J* = 13.0, and 3.0 Hz, 1H, 9-H_eq_), 2.94 (dd, *J* = 10.2, and 4.5 Hz, 1H, 1-H), 3.04 (dq, *J* = 10.0, and 1.6 Hz, 7-H), 4.40 (dd, *J* = 4.3 and 2.6 Hz, 1H, 3-H), 5.08 (ddd, *J* = 11.5, 10.0, and 3.0 Hz, 1H, 8-H), 5.27 (dq, *J* = 10.8, and 1.4 Hz, 1H, 5-H), 5.71 (br s, 1H, 13-H_b_), 6.12 (qq, *J* = 7.0 and 1.5 Hz, 1H, 3′-H), 6.22 (br m, 1H, 13-H_a_), 6.38 (dd, *J* = 10.8, and 1.6 Hz, 1H, 6-H) ppm. ^13^C-NMR (CD_3_OD, 125 MHz, extracted from HSQCC and HMBC spectra): δ 14.5 (CH_3_, 4′-C), 17.2 (CH_3_, 14-C), 19.1 (CH_3_, 5′-C), 21.9 (CH_3_, 15-C), 32.7 (CH_2_, 2-C), 46.8 (CH_2_, 9-C), 50.7 (CH, 7-C), 57.7 (C, 10-C), 60.7 (CH, 1-C), 69.4 (CH, 8-C), 71.2 (CH, 3-C), 76.1 (CH, 6-C), 124.3 (CH, 5-C), 126.0 (CH_2_, 13-C), 127.3 (C, 2′-C), 135.4 (C, 11-C), 138.4 (CH, 3′-C), 142.1 (C, 4-C), 166.8 (C, 1′-C), 170.3 (C, 12-C) ppm. HR-ESIMS calculated for C_20_H_26_O_6_Na [M + Na]^+^, 385.1622; found, 385.1637.

The ECD spectrum of **2** (Figure S62) was in agreement with the literature [14].

*(−)-(1R,4S,5R,10R)-1(10),4(5)-Diepoxynobilin* (**4**): *R*_f_ = 0.30 (SiO_2_, *n*-hexane/EtOAc 60:40). ${\left[\alpha \right]}_{D}^{25}$ = −16.2 (*c* = 0.1, CH_3_OH). ^1^H-NMR (CDCl_3_, 500 MHz): δ 1.40 (s, 3H, 15-CH_3_), 1.45 (overlapped, 1H, 9-H_ax_), 1.46 (br s, 3H, 14-CH_3_), 1.80 (overlapped, 1H, 2-H_ax_), 1.82 (quint, *J* = 1.5 Hz, 3H, 5′-CH_3_), 1.92 (dq, *J* = 7.0 and 1.5 Hz, 3H, 4′-CH_3_), 2.52 (dt, *J* = 15.0 and 4.2 Hz, 1H, 2-H_eq_), 2.60 (dd, *J* = 13.2, and 2.5 Hz, 1H, 9-H_eq_), 2.85 (d, *J* = 9.7 Hz, 1H, 5-H), 2.96 (dd, *J* = 10.6, and 4.2 Hz, 1H, 1-H), 3.07 (br d, *J* = 9.4 Hz, 7-H), 4.28 (dd, *J* = 4.2 and 3.0 Hz, 1H, 3-H), 5.02 (ddd, *J* = 12.0, 9.4 and 2.5 Hz, 1H, 8-H), 5.36 (br d, *J* = 9.7 Hz, 1H, 6-H), 5.73 (br m, 1H, 13-H_b_), 6.06 (qq, *J* = 7.2 and 1.5 Hz, 1H, 3′-H), 6.34 (br m, 1H, 13-H_a_) ppm. ^13^C-NMR (CDCl_3_, 125 MHz): δ 15.8 (CH_3_, 4′-C), 17.7 (CH_3_, 14-C), 20.0 (CH_3_, 15-C), 20.3 (CH_3_, 5′-C), 30.6 (CH_2_, 2-C), 47.4 (CH_2_, 9-C), 49.2 (CH, 7-C), 57.8 (C, 10-C), 58.9 (CH, 1-C), 62.4 (C, 4-C), 63.4 (CH, 5-C), 68.5 (CH, 8-C), 69.1 (CH, 3-C), 75.8 (CH, 6-C), 126.9 (C, 2′-C), 128.1 (CH_2_, 13-C), 133.3 (C, 11-C), 140.0 (CH, 3′-C), 166.1 (C, 1′-C), 168.9 (C, 12-C) ppm. HR-ESIMS calculated for C_20_H_26_O_7_Na [M + Na]^+^, 401.1571; found, 401.1588.

### 3.5. Synthesis of Compound ***3***

To a stirred solution of **1** (60 mg; 0.17 mmol) in DCM (3 mL), VO(acac)_2_ (14 mg; 0.05 mmol) and *t*BHP (solution 5.5 M in decane; 42 μL; 0.2 mmol) were added under ice and argon atmosphere. The reaction mixture was then allowed to stand at room temperature for 12 h. After TLC monitoring, the reaction was quenched by adding 10% Na_2_S_2_O_3_ (2 × 5 mL). The absence of peroxides was tested with KI paper. The organic phase was dried under Na_2_S_2_O_4_, the solvent was removed under reduced pressure, and the obtained residue was purified by chromatography (SiO_2_; *n*-hexane/AcOEt 70:30) to give **3** (54 mg; 0.15 mmol; 88%) as a white solid.

*(−)-(4S,5R)-4,5-Epoxynobilin* (**3**): *R*_f_ = 0.70 (SiO_2_, *n*-hexane/EtOAc 60:40). ${\left[\alpha \right]}_{D}^{25}$ = −6.19 (*c* = 0.1, CH_3_OH). ^1^H-NMR (CDCl_3_, 500 MHz): δ 1.34 (br s, 3H, 15-CH_3_), 1.82 (br s, 3H, 14-CH_3_), 1.84 (quint, *J* = 1.5 Hz, 3H, 5′-CH_3_), 1.93 (dq, *J* = 7.0 and 1.5 Hz, 3H, 4′-CH_3_), 2.23 (ddd, *J* = 14.4, 7.0 and 3.6 Hz, 1H, 2-H_eq_), 2.37 (dd, *J* = 12.0, and 11.0 Hz, 1H, 9-H_ax_), 2.59 (dd, *J* = 12.0, and 3.7 Hz, 1H, 9-H_eq_), 2.76 (dd, *J* = 14.4, 10.2, and 2.6 Hz, 1H, 2-H_ax_), 2.69 (br d, *J* = 9.7 Hz, 5-H), 3.05 (dddd, *J* = 9.7, 1.5,. 1.0, and 1.0 Hz, 7-H), 4.20 (dd, *J* = 3.6, and 2.6 Hz, 1H, 3-H), 4.92 (dd, *J* = 9.7, and 1.0 Hz, 1H, 6-H), 5.01 (ddd, *J* = 11.0, 9.7, and 3.7 Hz, 1H, 8-H), 5.40 (ddd, *J* = 10.2, 7.0, and 1.0 Hz, 1H, 1-H), 5.70 (dd, *J* = 1.5, and 0.8 Hz, 1H, 13-H_b_), 6.07 (qq, *J* = 7.0 and 1.5 Hz, 1H, 3′-H), 6.33 (br m, 1H, 13-H_a_) ppm. ^13^C-NMR (CDCl_3_, 125 MHz): δ 15.7 (CH_3_, 4′-C), 18.0 (CH_3_, 14-C), 20.0 (CH_3_, 15-C), 20.2 (CH_3_, 5′-C), 28.4 and 28.3 (CH_2_, 2-C), 46.8 (CH_2_, 9-C), 48.5 (CH, 7-C), 62.9 (C, 4-C), 63.8 (CH, 5-C), 69.3 (CH, 8-C), 73.2 and 73.1 (CH, 3-C), 77.4 (CH, 6-C), 122.9 (CH, 1-C), 127.1 (C, 2′-C), 127.4 (CH_2_, 13-C), 133.8 (C, 11-C), 135.0 (C, 10-C), 139.2 (CH, 3′-C), 166.2 (C, 1′-C), 169.1 (C, 12-C) ppm HR-ESIMS calculated for C_20_H_26_O_6_Na [M + Na]^+^, 385.1622; found, 385.1639. The ECD spectrum is reported in the Supplementary Materials (Figure S62).

### 3.6. General Procedure for Degradation with p-TsOH·H_2_O

*p*-TsOH·H_2_O (1–2 mg) was added to a solution of epoxy derivatives **2**–**4** and **11**–**12** (20 mg; 0.055 mmol for **2** and **3**; 0.053 mmol for **4**; 0.050 mmol for **11** and **12**, respectively) in dichloromethane (3 mL), and stirred at 20 °C. After the starting material (TLC on SiO_2_, *n*-hexane/EtOAc 40:60) disappeared, the reaction was quenched with aqueous Na_2_HPO_4_ 0.5 N (2 mL). The layers were separated, and the organic layer dried over Na_2_SO_4_ and concentrated in vacuo to give a brownish oil. The major reaction products were isolated by semi-preparative HPLC (gradient of H_2_O/CH_3_CN; detection at 220 nm).

*(+)-(4Z,1R,3R,6R,7R,8S,10S)-8-Angeloxy-3,10-epoxy-1-hydroxy-germacra-4(5),11(13)-dien-6,12-olide* (**5**): white solid (10 mg, 0.027 mmol, 50%); ${\left[\alpha \right]}_{D}^{25}$ = +6.85 (*c* 0.1, MeOH); for ^1^H and ^13^C-NMR data, see Table 1 and Table 3, respectively. HRESIMS *m*/*z* 385.1632 [M + Na]^+^ (calculated for C_20_H_26_NaO_6_: 385.1622).

*(4Z,9E,1R,3S,6R,7S,8S)-8-Angeloxy-1,3-dihydroxy-germacra-4(5),9(10),11(13)-trien-6,12-olide and (4Z,9Z,1R,3S,6R,7S,8S)-8-Angeloxy-1,3-dihydroxy-germacra-4(5),9(10),11(13)-trien-6,12-olide* (**6** and **7**): white solid (2 mg, 0.006 mol, 11% in a ratio of 3:1); for ^1^H and ^13^C-NMR data, see Table 1 and Table 3, respectively. HRESIMS *m*/*z* 385.1644 [M + Na]^+^ (calculated for C_20_H_26_NaO_6_: 385.1622).

*(+)-(1R,3R,4S,5R,6S,7R,8S,10S)-8-Angeloxy-3(10),4(5)-diepoxy-germacra-11(13)-en-6,12-olide* (**8**): white solid (8 mg, 0.02 mol, 40%); ${\left[\alpha \right]}_{D}^{25}$ = +3.75 (*c* 0.1, MeOH); for ^1^H and ^13^C-NMR data, see Table 1 and Table 3, respectively. HRESIMS *m*/*z* 401.1591 [M + Na]^+^ (calculated for C_20_H_26_NaO_7_: 401.1571).

*(−)-(1R,3R,4S,5S,6S,7R,8S,10S)-8-Angeloxy-3,10-epoxy-4-hydroxy-5-tosyloxygermacra-11(13)-en-6,12-olide* (**9**): white solid (2.7 mg, 0.005 mol, 10%); ${\left[\alpha \right]}_{D}^{25}$ = −2.9 (*c* 0.2, MeOH); for ^1^H and ^13^C-NMR data, see Table 1 and Table 3, respectively. HRESIMS *m*/*z* 573.1786 [M + Na]^+^ (calculated for C_27_H_34_NaO_10_S: 573.1765).

*(+)-(1Z,4Z,3S,6R,7R,8S,10S)-8-Angeloxy-3,10-epoxy-3-hydroxy-germacra-1(2),4(5),11(13)-trien-6,12-olide* (**10**): white solid (2.2 mg, 0.06 mol, 12%); ${\left[\alpha \right]}_{D}^{25}$ = +2.87 (*c* 0.1, MeOH); for ^1^H and ^13^C-NMR data, see Table 1 and Table 3, respectively. HRESIMS *m*/*z* 383.1500 [M + Na]^+^ (calculated for C_20_H_24_NaO_6_: 383.1465).

*(+)-(4Z,9E,1R,3S,6R,7S,8S)-3-Acetoxy-8-angeloxy-1-hydroxy-germacra-4(5),9(10),11(13)-trien-6,12-olide* (**13**): white solid (2 mg, 0.005 mol, 10%); ${\left[\alpha \right]}_{D}^{25}$ = +78.66 (*c* 0.5, MeOH); for ^1^H and ^13^C-NMR data, see Table 2 and Table 4, respectively. HRESIMS *m*/*z* 427.1749 [M + Na]^+^ (calculated for C_22_H_28_NaO_7_: 427.1727).

*(+)-(4Z,1R,3S,6R,7R,8S)-3-Acetoxy-8-angeloxy-1-hydroxy-germacra-4(5),10(14),11(13)-trien-6,12-olide* (**14**): white solid (1 mg, 0.002 mol, 5%); ${\left[\alpha \right]}_{D}^{25}$ = +51.75 (*c* 0.1, MeOH); for ^1^H and ^13^C-NMR data, see Table 2 and Table 4, respectively. HRESIMS *m*/*z* 427.1749 [M + Na]^+^ (calculated for C_22_H_28_NaO_7_: 427.1727).

### 3.7. General Procedure for Degradation with BF_3_·Et_2_O

To a solution of epoxy derivatives **2**–**4** and **11**–**12** (20 mg; 0.055 mmol for **2** and **3**; 0.053 mmol for **4**; 0.050 mmol for **11** and **12**, respectively) in anhydrous toluene (5 mL), BF_3_·Et_2_O (5 μL) was added under cooling in ice, and stirred at 20 °C. After disappearing of the starting material (TLC on SiO_2_, *n*-hexane/EtOAc 40:60) the reaction was quenched with aqueous Na_2_HPO_4_ 0.5 N (10 mL). The layers were separated, and the organic layer dried over Na_2_SO_4_ and concentrated to dryness under reduced pressure. The major reaction products were isolated by semi-preparative HPLC (gradient of CH_3_CN/H_2_O; detection at 220 nm). Yields for individual compounds are reported in Scheme 2, Scheme 3 and Scheme 4.

*(+)-(7R,8S,10S)-8-Angeloxy-3-hydroxy-7,8,9,10-tetrahydro-cadalen-11(13)-en-12-oic acid* (**15**): white solid (6.8 mg, 0.02 mol, 40%); ${\left[\alpha \right]}_{D}^{25}$ = +82.95 (*c* 0.1, MeOH); for ^1^H and ^13^C-NMR data, see Table 2 and Table 4, respectively. HRESIMS *m*/*z* 367.1530 [M + Na]^+^ (calculated for C_20_H_24_NaO_5_: 367.1516).

*Cadalen-11(13)-en-12-oic acid* (**16**): white solid (1.1 mg, 0.005 mol, 10%); for ^1^H and ^13^C-NMR data, see Table 2 and Table 4, respectively. HRESIMS *m*/*z* 249.0899 [M + Na]^+^ (calculated for C_15_H_14_NaO_2_: 249.0886).

### 3.8. General Procedure for Synthesis of Compounds ***11*** and ***12***

To a stirred solution of epoxy derivatives **2** and **3** (20 mg: 0.055 mmol) in dichlomethane (2 mL), acetic anhydride (35 μL; 0.37 mmol), Hünig’s base (20 μL; 0.12 mmol) and DMAP (cat.) were added. After disappearance of the starting material (TLC on SiO_2_, *n*-hexane/EtOAc 70:30) the reaction was quenched by adding HCl 0.5 M (2 mL). The layers were separated and the organic layer dried over Na_2_SO_4_ and concentrated to dryness under reduced pressure. Compounds **11** (17.8 mg; 80%) and **12** (15.5 mg; 70%) were isolated by chromatography on SiO_2_ (*n*-hexane/EtOAc 70:30; detection with phosphomolybdic acid).

*(+)-(1R,10R)-3-Acetoxy-1,10-epoxy nobilin* (**11**): *R*_f_ = 0.3 (SiO_2_, hexane/EtOAc 60:40). ${\left[\alpha \right]}_{D}^{25}$ = +13.12 (*c* = 0.1, CHCl_3_). ^1^H-NMR (CDCl_3_, 500 MHz): δ 1.36 (dd, *J* = 13.2, and 11.3 Hz, 1H, 9-H_ax_), 1.52 (s, 3H, 14-CH_3_), 1.66 (br dd, *J* = 10.2, and 4.5 Hz, 1H, 2-H_ax_), 1.81 (quint, *J* = 1.5 Hz, 3H, 5′-CH_3_), 1.85 (d, *J* = 1.5 Hz, 3H, 15-CH_3_), 1.91 (dq, *J* = 7.0 and 1.5 Hz, 3H, 4′-CH_3_), 2.08 (s, 3H,2″), 2.53 (ddd, *J* = 15.5, 5.0 and 4.5 Hz, 1H, 2-H_eq_), 2.58 (dd, *J* = 13.2, and 3.0 Hz, 1H, 9-H_eq_), 2.83 (br dd, *J* = 10.2, and 4.5 Hz, 1H, 1-H), 2.85 (dq, *J* = 10.5, and 1.5 Hz, 7-H), 5.06 (ddd, *J* = 11.3, 10.5, and 3.0 Hz, 1H, 8-H), 5.20 (dd, *J* = 5.0, and 2.2 Hz, 1H, 3-H), 5.22 (dq, *J* = 11.0, and 1.5 Hz, 1H, 5-H), 5.65 (dd, *J* = 11.0, and 1.5 Hz, 1H, 6-H), 5.66 (br m, 1H, 13-H_b_), 6.05 (qq, *J* = 7.0 and 1.5 Hz, 1H, 3′-H), 6.29 (br m, 1H, 13-H_a_), ppm. ^13^C-NMR (Extracted from HSQC and HMBC spectra; CDCl_3_, 125 MHz): δ 15.6 (CH_3_, 4′-C), 17.2 (CH_3_, 14-C), 20.2 (CH_3_, 5′-C), 21.0 (CH_3_, 2″), 22.8 (CH_3_, 15-C), 30.2 (CH_2_, 2-C), 46.8 (CH_2_, 9-C), 50.7 (CH, 7-C), 57.7 (C, 10-C), 60.3 (CH, 1-C), 69.0 (CH, 8-C), 72.6 (CH, 3-C), 75.2 (CH, 6-C), 125.0 (CH, 5-C), 127.0 (CH_2_, 13-C), 127.4 (C, 2′-C), 134.0 (C, 11-C), 139.0 (CH, 3′-C), 139.0 (C, 4-C), 166.4 (C, 1′-C), 168.9 (C, 12-C), 169.3 (C, 1″_3_) ppm. HR-ESIMS calculated for C_22_H_28_O_7_Na [M + Na]^+^, 427.1727; found, 427.1753.

*(+)-(4S,5R)-3-Acetoxy-4,5-epoxy nobilin* (**12**): *R*_f_ = 0.2 (SiO_2_, *n*-hexane/EtOAc 60:40). ${\left[\alpha \right]}_{D}^{25}$ = +94.97 (*c* = 0.1, CHCl_3_). ^1^H-NMR (CDCl_3_, 500 MHz): δ 1.36 (br s, 3H, 15-CH_3_), 1.79 (br s, 3H, 14-CH_3_), 1.83 (quint, *J* = 1.5 Hz, 3H, 5′-CH_3_), 1.92 (dq, *J* = 7.0 and 1.5 Hz, 3H, 4′-CH_3_), 2.02 (s, 3H,2″), 2.37 (ddd, *J* = 15.0, 7.0 and 3.0 Hz, 1H, 2-H_eq_), 2.40 (dd, *J* = 12.8, and 11.0 Hz, 1H, 9-H_ax_), 2.56 (br d, *J* = 9.7 Hz, 5-H), 2.58 (dd, *J* = 12.8, and 3.6 Hz, 1H, 9-H_eq_), 2.76 (dd, *J* = 15.0, 10.0, and 2.5 Hz, 1H, 2-H_ax_), 3.06 dq, *J* = 9.9, 1.5 Hz, 7-H), 4.61 (dd, *J* = 9.7, and 1.5 Hz, 1H, 6-H), 5.03 (ddd, *J* = 11.0, 9.9, and 3.6 Hz, 1H, 8-H), 5.36 (br t, *J* = 3.0 Hz, 1H, 3-H), 5.48 (ddd, *J* = 10.0, 7.0, and 1.0 Hz, 1H, 1-H), 5.69 (br m, 1H, 13-H_b_), 6.05 (qq, *J* = 7.0 and 1.5 Hz, 1H, 3′-H), 6.31 (br m, 1H, 13-H_a_) ppm. ^13^C-NMR (Extracted from HSQC and HMBC spectra; CDCl_3_, 125 MHz): δ 15.7 (CH_3_, 4′-C), 18.0 (CH_3_, 14-C), 20.2 (CH_3_, 5′-C), 20.5 (CH_3_, 15-C), 20.9 (CH_3_,2″), 27.6 (CH_2_, 2-C), 46.8 (CH_2_, 9-C), 48.7 (CH, 7-C), 60.5 (C, 4-C), 62.4 (CH, 5-C), 69.4 (CH, 8-C), 72.9 (CH, 3-C), 77.6 (CH, 6-C), 122.8 (CH, 1-C), 127.0 (C, 2′-C), 127.3 (CH_2_, 13-C), 134.0 (C, 11-C), 135.0 (C, 10-C), 139.2 (CH, 3′-C), 166.1 (C, 1′-C), 169.0 (C, 12-C), 169.2 (C,2″) ppm. HR-ESIMS calculated for C_22_H_28_O_7_Na [M + Na]^+^, 427.1727; found, 427.1748.

### 3.9. Computational Methods

Conformational analysis of parthenolide, **2**, **3**, and **5** was performed with Schrödinger MacroModel 9.8 (Schrödinger, LLC, New York, NY, USA) employing the OPLS_2005 (Optimized Potentials for Liquid Simulations) force field. For parthenolide, **2** and **3**, the calculations were done with the chloroform solvation model (N.B. the dichloromethane solvation model is not available within MacroModel). Conformers within a 2.0 kcal/mol energy window from the global minimum were selected for geometrical optimization and energy calculation. Structural optimizations, transition state (TS) searches, and vibrational calculations were carried out by applying DFT with the Becke’s nonlocal three-parameter exchange and correlation functional, and the Lee–Yang–Parr correlation functional level (B3LYP) using the 6-31+G(d,p) basis set in combination with the SMD solvation model [21] (solvent = dichloromethane), as implemented in the Gaussian 09 program package [27]. At optimized geometries, vibrational analysis was performed, yielding one imaginary frequency for the transition states and no imaginary frequencies for other molecular species.

## 4. Conclusions

A study of the reactivity of nobilin epoxy derivatives in acidic media has been carried out through chemical and theoretical analysis. In agreement with what has been reported for similar epoxy heliangolides [28], the type of rearrangement that compounds **2**–**4** undergo under acidic catalysis depends solely on their conformation, which activates or deactivates the latent reactivity of functional groups towards a structural rearrangement. Provided that the epoxy group and olefin, can provide an nucleophilic site that can trigger a subsequent rearrangement, the reactivity of the 1,10-epoxide towards electrophiles, similar to what has been observed for the corresponding 1(10)-olefin of **1**, is much higher than that of the 4,5-epoxide. A key role in deactivating the reactivity of the latter epoxide could be played by the 3-hydroxy group, which stabilizes the germacranolide skeleton through non-conventional hydrogen bonding with H-6. This assumption would explain the different reactivity of parthenolide compared to that of compounds **3** and **13**. Nevertheless, the high reactivity of the hydroxyl function at C-3 towards the C-10 carbocation provides easy access to the class of furanoheliangolides with good yield. Since many of the isolated products are structurally related to natural products, the present study provides new insight into the mechanism of natural sesquiterpene-forming reactions.

## Figures and Tables

**Table 1:** ^1^H-NMR spectroscopic data for compounds **5**–**10** (CD_3_OD for **5**–**9**, CDCl_3_ for **10**; 500.13 MHz; δ in ppm).

Position	5 δ_H_ (mult, *J* in Hz)	6 δ_H_ (mult, *J* in Hz)	7 δ_H_ (mult, *J* in Hz)	8 δ_H_ (mult, *J* in Hz)	9 ^b^ δ_H_ (mult, *J* in Hz)	10 δ_H_ (mult, *J* in Hz)
1	3.85 (br d, 4.6)	4.05 (dd, 10.6, 6.5)	4.96 (dd, 10.2, 3.0)	3.90 (d, 4.3)	3.96 (dd, 5.2, 1.4)	6.22 (d, 5.7)
2	2.32–2.20 ^a^	1.94 ^a^ 2.36 (ddd, 14.5, 6.6, 4.5)	2.03 (ddd, 14.5, 3.8, 3.0) 2.19 (ddd, 14.5, 10.2, 3.7)	2.12 (dd, 13.8, 6.8) 2.44 (ddd, 13.8, 11.0, 4.3)	1.82 (ddd, 13.2, 6.2, 1.4) 2.41 (ddd, 13.2, 11.4, 5.2)	5.89 (d, 5.7)
3	4.79 (dd, 8.6, 8.3)	4.49 ^a^	4.49 ^a^	4.63 (dd, 11.0, 6.8)	4.22 (dd, 11.4, 6.2)	-
4	-	-	-	-	-	-
5	5.26 (dq, 7.2, 1.5)	5.27 (dq, 10.5, 1.5)	5.39 (dq, 10.5, 1.5)	3.06 (d, 7.3)	4.79 (br s)	5.48 (dq, 5.8, 1.2)
6	6.14 ^a^	6.00 (dd, 10.5, 9.5)	6.38 (dd, 10.5, 9.2)	5.25 (dd, 7.3, 5.5)	5.63 (br d, 5.7)	5.95 (ddq, 5.8, 5.2, 1.2)
7	3.08 (dddd, 9.0, 7.5, 3.0, 3.0)	3.09 (dddd, 9.5, 9.5, 3.5, 3.0)	3.02 (dddd, 9.5, 9.2, 3.5, 3.0)	3.35 (dddd, 9.0, 5.5, 2.6, 2.5)	3.31 (m)	3.26 (dddd, 10.5, 5.2, 2.8, 2.5)
8	5.33 (dd, 9.5, 9.0)	5.57 (dd, 10.2, 9.5)	6.00 (dd, 10.5, 9.5)	5.00 (dd, 9.8, 9.0)	5.06 (ddd, 10.0, 6.9, 1.2)	5.17 (ddd, 10.5, 5.0, 3.2)
9	2.09 (dd, 14.8, 9.5) 1.70 (d, 14.8)	5.18 (dq, 10.2, 1.5)	5.30 (dq, 10.5, 1.5)	1.87 ^a^ β 2.05 (br dd, 14.6, 9.8)	1.91 ^a^ 2.13 (dd, 14.3, 10.3)	2.14 (dd, 15.4, 5.0) 2.22 (dd, 15.4, 3.2)
10	-	-	-	-	-	-
11	-	-	-	-	-	-
12	-	-	-	-	-	-
13	5.65 (dd, 3.0, 0.7) 6.17 ^a^	5.71 (d, 3.0) 6.16 (d, 3.5)	5.85 (d, 3.0) 6.25 (d, 3.5)	5.74 (br d, 2.5) 6.23 (br d, 2.8)	5.41 (br d, 2.5) 5.86 (br d, 2.8)	5.89 (br d, 2.5) 6.22 br d, 2.8)
14	1.49 (s)	1.95 (d, 1.5)	1.80 (d, 1.5)	1.37 (br s)	1.33 (s)	1.53 (s)
15	1.66 (br s)	1.76 (d, 1.5)	1.79 (d, 1.5)	1.27 (s)	1.18 (br s)	1.73 (t, 1.2)
1′	-	-	-	-	-	-
2′	-	-	-	-	-	-
3′	6.17 (qq, 7.0, 1.5)	6.20 (qq, 7.0, 1.5)	6.20 (qq, 7.0, 1.5)	6.16 (qq, 7.0, 1.5)	6.15 (br q, 7.2)	6.16 (br q, 7.2)
4′	1.96 (dq, 7.0, 1.5)	1.95 (dq 7.0, 1.5)	1.98 (dq 7.0, 1.5)	1.94 (dq 7.0, 1.5)	1.91 (br d, 7.2)	2.00 (br d, 7.2)
5′	1.89 (quint, 1.5)	1.89 (quint, 1.5)	1.91 (quint, 1.5)	1.88 (quint, 1.5)	1.84 (quint, 1.5)	1.91 (quint, 1.5)

^a^ Overlapped signals; ^b^ *p*-toluenesulphonate group: H-2, H-2′ δ_H_ = 7.76 pseudo d, *J* = 8.3 Hz; H-3, H-3′ δ_H_ = 7.41 pseudo d, *J* = 8.3 Hz; -CH*_3_* δ_H_ = 2.45, s.

**Table 2:** ^13^C-NMR spectroscopic data for compounds **5**–**8** (CD_3_OD for **5**–**9**, CDCl_3_ for **10**; 125.77 MHz; δ in ppm).

Position	5 δ_C_	6 δ_C_	7 δ_C_^a^	8 δ_C_	9 δ_C_^c^	10 δ_C_^a^
1	77.6, CH	76.2, CH	69.4, CH	77.9, CH	78.8, CH	139.3, CH
2	41.8, CH_2_	38.9, CH_2_	37.0, CH_2_	39.0, CH_2_	36.6, CH_2_	128.3, CH
3	81.7, CH	74.1, CH	74.9, CH	78.6, CH	85.3, CH	110.4, C
4	141.9, C	142.2, C	145.9, C	66.2, C	75.2, C	137.8, C
5	124.0, CH	126.7, CH	125.5, CH	68.7, CH	89.3, CH	129.6, CH
6	78.5, CH	76.8, CH	78.0, CH	82.7, CH	82.5, CH	76.4, CH
7	52.2, CH	50.9, CH	52.1, CH	49.09, CH	48.0, CH	48.2, CH
8	74.1, CH	74.0, CH	72.5, CH	73.3, CH	74.0, CH	71.5, CH
9	46.8, CH_2_	126.8, CH	121.9, CH	49.15, CH_2_	49.3, CH_2_	42.9, CH_2_
10	87.3, C	143.2, C	144.1, C	87.1, C	85.8, C	87.3, C
11	139.0, C	140.1, C	140.0, C	137.2, C	137.1, C	137.7, C
12	171.8, C	172.0, C	172.0, C	171.7, C	171.3, C	169.6, C
13	124.8, CH_2_	121.8, CH_2_	121.8, CH_2_	127.0, CH_2_	125.5, CH_2_	123.8, CH_2_
14	19.6, CH_3_	11.7, CH_3_	19.5, CH_3_	20.7, CH_3_	22.6, CH_3_	28.0, CH_3_
15	20.2, CH_3_	22.8, CH_3_	23.6, CH_3_	20.5 ^b^, CH_3_	24.2, CH_3_	20.2, CH_3_
1′	167.9, C	168.2, C	168.2, C	168.0, C	168.3, C	167.4, C
2′	128.7, C	128.7, C	128.6, C	128.7, C	127.0, C	126.2, C
3′	140.4, CH	139.9, CH	140.0, CH	14.03, CH	140.3, CH	139.4, CH
4′	16.1, CH_3_	16.0, CH_3_	16.0, CH_3_	16.0, CH_3_	16.0, CH_3_	15.7, CH_3_
5′	20.7, CH_3_	20.8, CH_3_	20.9, CH_3_	20.6 ^b^, CH_3_	20.5, CH_3_	20.6, CH_3_

^a^ Extracted from ^1^H-^13^C 2D inverse detected experiments due to low amount of sample; ^b^ interchangeable within the same column; ^c^ *p*-toluensulphonate group: C-1 (134.7), C-2/C-2′ (129.0), C-3/C-3′ (131.4), C-4 (147.2), -CH_3_ (21.6).

**Table 3:** ^1^H-NMR spectroscopic data for compounds **13**–**16** (CD_3_OD; 500.13 MHz; δ in ppm).

Position	13 δ_H_ (mult, *J* in Hz)	14 δ_H_ (mult, *J* in Hz)	15 δ_H_ (mult, *J* in Hz)	16 δ_H_ (mult, *J* in Hz)
1	4.11 (dd, 10.6, 6.5)	4.01 (dd, 11.7, 3.0)	-	-
2	1.91 ^a^ 2.43 (ddd, 15.5, 6.5, 6.0)	2.17 ^a^ (ddd, 15.0, 5.0, 3.0) 2.47 (ddd, 15.0, 11.7, 2.5)	6.65 (s)	7.91 (d, 8.5)
3	5.49 (br d, 6.0)	5.30 ^a^	-	7.34 (br d, 8.5)
4	-	-	-	-
5	5.32 (dq, 8.5, 1.2)	5.32 (dq, 10.3, 1.2)	6.65 (s)	7.58 (br s)
6	5.28 ^a^	5.87 (dd, 10.3, 2.0)	-	-
7	3.17 (dddd, 9.5, 8.5, 3.5, 3.0)	3.20 (dddd, 10.7, 2.0, 1.7, 1.5)	3.97 (d, 5.8)	-
8	5.65 (dd, 10.5, 9.5)	5.03 (td, 10.5, 4.0)	5.50 (ddd, 7.5, 5.7, 2.7)	7.19 (d, 7.2)
9	5.24 (dq, 10.5, 1.2)	2.49 ^a^ 2.90 (br dd, 13.6, 4.2)	1.80 ^a^ 2.00 (ddd, 13.0, 7.5, 6.0)	7.20 (d, 7.2)
10	-	-	3.97 (sextet, 7.0)	-
11	-	-	-	-
12	-	-	-	-
13	5.74 (d, 3.0)	5.75 (d, 1.5)	5.31 (br s)	5.74 (d, 1.8)
	6.17 (d, 3.5)	6.23 (d, 1.7)	6.33 (br s)	6.51 (d, 1.8)
14	1.97 (d, 1.2)	5.48 (br s) 5.51 (br s)	1.34 (d, 7.0)	2.65 (s)
15	1.85 (d, 1.2)	1.85 (d, 1.2)	2.10 (s)	2.46 (br s)
1′	-	-	-	-
2′	-	-	-	-
3′	6.19 (qq, 7.0, 1.5)	6.15 (qq, 7.0, 1.5)	6.03 (qq, 7.0, 1.5)	-
4′	1.99 (dq, 7.0, 1.5)	1.96 (dq, 7.0, 1.5)	1.81 (dq, 7.0, 1.5)	-
5′	1.92 (quint, 1.5)	1.90 (quint, 1.5)	1.79 (quint, 1.5)	-
1″	-	-	-	-
2″	2.02, (s)	2.04, (s)	-	-

^a^ Overlapped signals.

**Table 4:** ^13^C-NMR spectroscopic data for compounds **13**–**16** (CD_3_OD; 125.77 MHz; δ in ppm).

Position	13 δ_C_^a^	14 δ_C_^a^	15 ^a^ δ_C_	16 ^a^ δ_C_
1	75.4, CH	70.6, CH	140.5, C	132.5, C
2	35.2, CH_2_	34.5, CH_2_	113.9, CH	125.0, CH
3	74.6, CH	73.6, CH	155.0, C	128.4, CH
4	137.9, C	139.4, C	124.0, C	136.4, C
5	127.0, CH	124.8, CH	132.0, CH	126.1, CH
6	76.0, CH	75.0, CH	126.6, C	128.6, CH
7	50.0, CH	49.1, CH	48.3, CH	135.60, C
8	72.8, CH	73.6, CH	71.9, CH	127.4, CH
9	127.6, CH	39.8, CH_2_	33.6, CH_2_	126.0, CH
10	141.7, C	144.4, C	31.5, CH	135.4, C
11	139.0, C	135.2, C	145.1, C	145.5, C
12	170.8, C	170.0, C	170.1, C	171.8, C
13	121.3, CH_2_	125.7, CH_2_	129.1, CH_2_	127.6, CH_2_
14	11.1, CH_3_	116.5, CH_2_	22.9, CH_3_	19.4, CH_3_
15	22.2, CH_3_	21.7, CH_3_	15.6, CH_3_	21.7, CH_3_
1′	167.6, C	166.8, C	169.4, C	-
2′	128.0, C	127.2, C	129.5, C	-
3′	139.5, CH	138.5, CH	138.8, CH	-
4′	15.6, CH_3_	14.6, CH_3_	15.4, CH_3_	-
5′	20.3, CH_3_	19.0, CH_3_	20.5, CH_3_	-
1″	169.2, C	170.0, C	-	-
2″	20.9, CH_3_	19.7, CH_3_	-	-

^a^ Extracted from ^1^H-^13^C 2D inverse detected experiments due to low amount of sample.

**Table 5:** In vitro activity of compounds **1**, **3**–**8**, and **10**–**15** against *T. b. rhodesiense* (STIB 900), *T. cruzi* (Tulahuen C4 LacZ), *L. donovani* (MHOM-ET-67/L82), *P. falciparum* (NF54), and cytotoxicity in L6 cells.

Compound	*T. b. rhodesiense* IC_50_ (μM) ^a^	*T. cruzi* IC_50_ (μM) ^a^	*L. donovani* IC_50_ (μM) ^a^	*P. falciparum* IC_50_ (μM) ^a^	L6 cells IC_50_ (μM) ^a^
1	2.08± 0.09 (2.4) ^b^	11.3± 3.3 (0.4) ^b^	2.69± 1.08 (1.8) ^b^	3.12± 0.52 (1.6) ^b^	4.91 ± 0.53
3	1.96± 0.16 (2.04) ^b^	4.24 (0.9) ^h^	13.19± 3.31 (0.3) ^b^	2.39 (1.7) ^h^	4.0 ± 0.03
4	2.18 ± 0.49 (2.6) ^b^	8.38 (0.7) ^h^	28.67 ± 8.95 (0.2) ^b^	3.20 (1.7) ^h^	5.56 ± 0.30
5	0.91 ± 0.16 (3.7) ^b^	6.35 (0.5) ^h^	8.28 ± 2.40 (0.4) ^b^	4.06 (0.8) ^h^	3.33 ± 0.28
6–7	1.21 ± 0.16 (3.9) ^b^	16.00 ± 0.34 (0.3) ^b^	12.88 ± 0.65 (0.4) ^b^	13.82 ± 1.59 (0.3) ^b^	4.74 ± 0.24
8	3.25 ± 0.50 (3.5) ^b^	47.9 ± 1.11 (0.24) ^b^	66.8 ± 7.79 (0.17) ^b^	31.3 ± 2.40 (0.4) ^b^	11.5 ± 0.72
10	13.4 ± 0.46 (1.51) ^b^	132.19 ± 0.59 (0.15) ^b^	35.96 ± 0.18 (0.56) ^b^	79.13 ± 1.02 (0.26) ^b^	20.20 ± 2.098
11	1.57 ± 0.005 (0.28) ^b^	11.49 ± 0.68 (0.2) ^b^	4.20 ± 0.28 (0.55) ^b^	3.93 ± 0.53 (0.59) ^b^	2.31 ± 0.196
12	0.47 ± 0.006 (2.19) ^b^	2.60 ± 0.69 (0.5) ^b^	1.30 ± 0.03 (0.79) ^b^	1.55 ± 0.31 (0.66) ^b^	1.03 ± 0.076
13	0.54 ± 0.013 (3.46) ^b^	12.76 ± 1.08 (0.15) ^b^	3.26 ± 0.22 (0.57) ^b^	9.17 ± 0.61 (0.20) ^b^	1.87 ± 0.026
14	1.20 ± 0.151 (4.4) ^b^	15.4 ± 1.48 (0.6) ^b^	4.97 ± 0.43 (7.2) ^b^	8.80 ± 0.94 (0.68) ^b^	3.95 ± 0.088
15	13.32 ± 0.333 (1.68) ^b^	36.81 ± 7.76 (0.61) ^b^	5.86 ± 0.59 (3.83) ^b^	35.66 ± 1.10 (0.63) ^b^	22.44 ± 0.157
Positive control	0.01 ± 0.01 ^c^	2.0 ± 0.2 ^d^	0.13 ± 0.01 ^e^	0.006 ± 0.01 ^f^	0.016 ± 0.01 ^g^

^a^ Average of two independent assays in μg/mL; ^b^ Selectivity Index (SI): IC_50_ in L6 cells divided by IC_50_ in the titled parasitic strain; ^c^ melarsoprol; ^d^ benznidazole; ^e^ miltefosine; ^f^ chloroquine; ^g^ podophyllotoxin; ^h^ one assay.

## Supplementary Materials

Supplementary data associated with this article can be found in the online version.
