# Supplementary material for: Acid-Induced Rearrangement of Epoxygermacranolides: Synthesis of Furanoheliangolides and Cadinanes from Nobilin
Source: Molecules. 2017 Dec 18;22(12):2252. doi: 10.3390/molecules22122252 (PMC6149915; doi:10.3390/molecules22122252)
Supplement: Supplementary file 1 [file molecules-22-02252-s001.pdf]

Acid-Induced Rearrangement of Epoxygermacranolides: Synthesis of Furanoheliangolides and Cadinanes from Nobilin

Maria De Mieri [1, *], Martin Smieško [2], Isidor Ismajili [1], Marcel Kaiser [3, 4], and Matthias Hamburger [1, *]

[1] Pharmaceutical Biology, Pharmazentrum, University of Basel, Klingelbergstrasse 50, 4056 Basel, Switzerland; mariademieri@gmail.com, isidor.ismajili@gmail.com, Matthias.Hamburger@unibas.ch

[2] Department of Molecular Modeling, Pharmazentrum, University of Basel, Klingelbergstrasse 50, 4056 Basel, Switzerland; martin.smiesko@unibas.ch

[3] Department of Medical Parasitology & Infection Biology, Swiss Tropical and Public Health Institute, Socinstrasse 57, 4000 Basel, Switzerland; and University of Basel, Petersplatz 1, 4001 Basel, Switzerland; marcel.kaiser@swisstph.ch

[4] University of Basel, Petersplatz 1, 4001 Basel, Switzerland.

* Correspondence: Matthias.Hamburger@unibas.ch; mariademieri@gmail.com

Content

Figure S1: ^{1}H-NMR spectrum of nobilin **1** in CD_3OD.

Figure S2: ^{13}C spectrum of nobilin **1**in CD_3OD.

Figure S3. ^{1}H NMR spectrum of compound **2** in CD_3OD.

Figure S4. ^{1}H-^{1}H-COSY spectrum of compound **2** in CD_3OD.

Figure S5. HSQC (green) and HMBC (red) overlaid spectra of compound **2** in CD_3OD.

Figure S6: 2D ^{1}H-^{1}H NOESY spectrum of compound **2** in CD_3OD.

Figure S7. ^{1}H NMR spectrum of compound **3** in $CDCl_3$.

Figure S8: ^{13}C spectrum of compound **3** in $CDCl_3$.

Figure S9. ^{1}H-^{1}H-COSY spectrum of compound **3** in $CDCl_3$.

Figure S10. HSQC (green) and HMBC(red) overlaid spectra of compound **3** in $CDCl_3$.

Figure S11: 2D ^{1}H-^{1}H NOESY spectrum of compound **3** in $CDCl_3$.

Figure S12. ^{1}H NMR spectrum of compound **4** in $CDCl_3$.

Figure S13: ^{13}C spectrum of compound **4** in $CDCl_3$.

Figure S14. ^{1}H-^{1}H-COSY spectrum of compound **4** in $CDCl_3$.

Figure S15. HSQC (green) and HMBC(red) overlaid spectra of compound **4** in $CDCl_3$.

Figure S16: 2D ^{1}H-^{1}H NOESY spectrum of compound **4** in $CDCl_3$.

Figure S17: ^{1}H-NMR spectrum of compound **5** in CD_3OD.

Figure S18: ^{13}C spectrum of compound **5** in CD_3OD.

Figure S19: ^{1}H-^{1}H COSY spectrum of compound **5** in CD_3OD.

Figure S20: Overlay of HSQC and HMBC spectra of compound **5** in CD_3OD.

Figure S21: 2D ^{1}H-^{1}H NOESY spectrum of compound **5** in CD_3OD.

Figure S22: 1D Selective NOESY spectrum of compound **5** in CD_3OD (D8 = 0.3 sec).

Figure S23: DFT 6-31+G(d,p) optimized structure of **5**

Figure S24: ^{1}H-NMR spectrum of compounds **6**-**7** in CD_3OD.

Figure S25: ^{13}C spectrum of compounds **6**-**7** in CD_3OD.

Figure S26: ^{1}H-^{1}H COSY spectrum of compounds **6**-**7** in CD_3OD.

Figure S27: Overlay of HSQC and HMBC spectra of compounds **6**-**7** in CD_3OD.

Figure S28: 2D ^{1}H-^{1}H NOESY spectrum of compounds **6**-**7** in CD_3OD.

Figure S29: ^{1}H-NMR spectrum of compound **8** in CD_3OD.

Figure S30: ^{13}C spectrum of compound **8** in CD_3OD.

Figure S31: ^{1}H-^{1}H COSY spectrum of compound **8** in CD_3OD.

Figure S32: Overlay of HSQC and HMBC spectra of compound **8** in CD_3OD.

Figure S33: 2D ^{1}H-^{1}H NOESY spectrum of compound **8** in CD_3OD.

Figure S34: ^{1}H-NMR spectrum of compound **9** in CD_3OD.

Figure S35: ^{13}C spectrum of compound **9** in CD_3OD.

Figure S36: ^1H-^1H COSY spectrum of compound **9** in CD_3OD.

Figure S37: Overlay of HSQC and HMBC spectra of compound **9** in CD_3OD.

Figure S38: 2D ^1H-^1H NOESY spectrum of compound **9** in CD_3OD.

Figure S39: ^1H NMR spectrum of compound **10** in CD_3OD.

Figure S40: ^1H-^1H-COSY spectrum of compound **10** in CD_3OD.

Figure S41: HSQC (green) and HMBC (red) overlaid spectra of compound **10** in CD_3OD.

Figure S42: 2D ^1H-^1H NOESY spectrum of compound **10** in CD_3OD.

Figure S43: ^1H-NMR spectrum of compound **11** in $CDCl_3$.

Figure S44: Overlay of HSQC and HMBC spectra of compound **11** in $CDCl_3$.

Figure S45: ^1H-NMR spectrum of compound **12** in $CDCl_3$.

Figure S46: Overlay of HSQC and HMBC spectra of compound **12** in $CDCl_3$.

Figure S47: ^1H NMR spectrum of compound **13** in CD_3OD.

Figure S48: ^1H-^1H-COSY spectrum of compound **13** in CD_3OD.

Figure S49: HSQC (green) and HMBC (red) overlaid spectra of compound **13** in CD_3OD.

Figure S50: 2D ^1H-^1H NOESY spectrum of compound **13** in CD_3OD.

Figure S51: ^1H NMR spectrum of compound **14** in CD_3OD.

Figure S52: ^1H-^1H-COSY spectrum of compound **14** in CD_3OD.

Figure S53: HSQC (green) and HMBC (red) overlaid spectra of compound **14** in CD_3OD.

Figure S54: 2D ^1H-^1H NOESY spectrum of compound **14** in CD_3OD.

Figure S55: ^1H NMR spectrum of compound **15** in CD_3OD.

Figure S56: ^1H-^1H-COSY spectrum of compound **15** in CD_3OD.

Figure S57: HSQC (green) and HMBC (red) overlaid spectra of compound **15** in CD_3OD.

Figure S58: 2D ^1H-^1H NOESY spectrum of compound **15** in CD_3OD.

Figure S59: ^1H NMR spectrum of compound **16** in CD_3OD.

Figure S60: ^1H-^1H-COSY spectrum of compound **16** in CD_3OD.

Figure S61: HSQC (green) and HMBC (red) overlaid spectra of compound **16** in CD_3OD.

Figure S62: ECD spectra in MeOH of compounds **2**, **3**, **5**, **8**-**10**.

Figure S63: Overlay of ^1H NMR spectra of **3** (a) and of the reaction of **3** with *p*-TSOH after 24 hours (b).

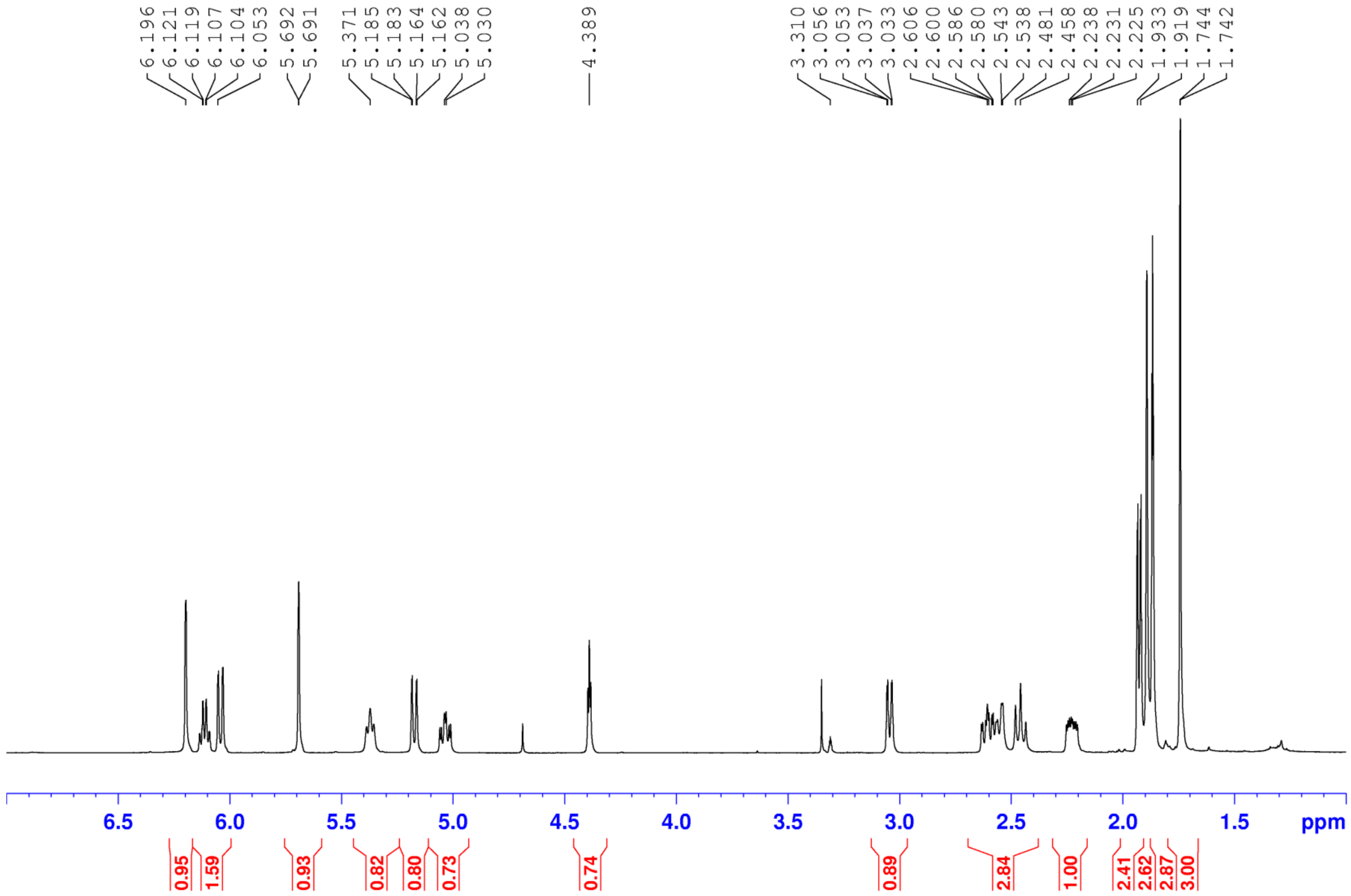

Fig. S1. ^{1}H NMR spectrum of nobilin **1** in CD_3OD.

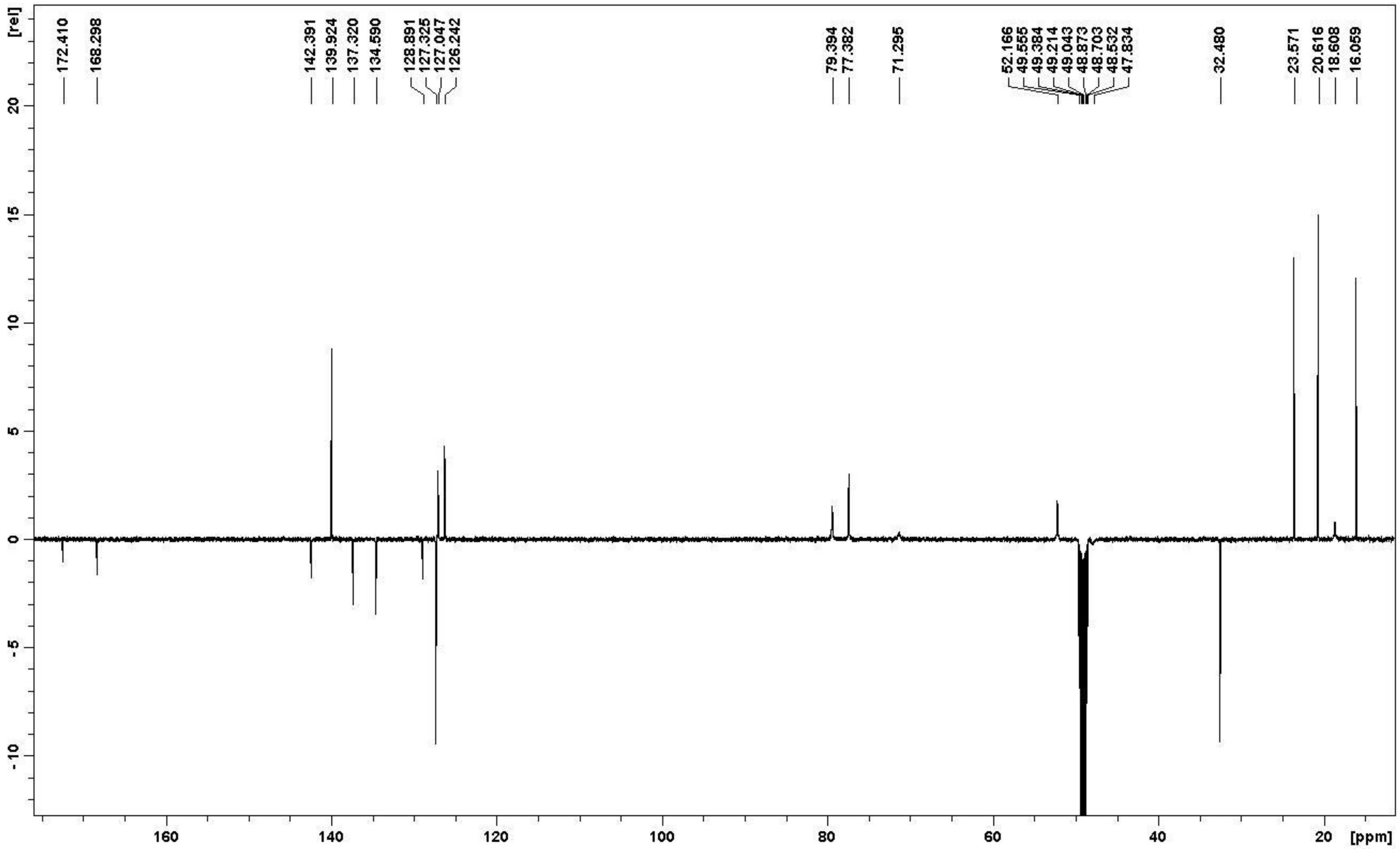

Fig. S2. ^{13}C spectrum of nobilin **1** in CD_3OD.

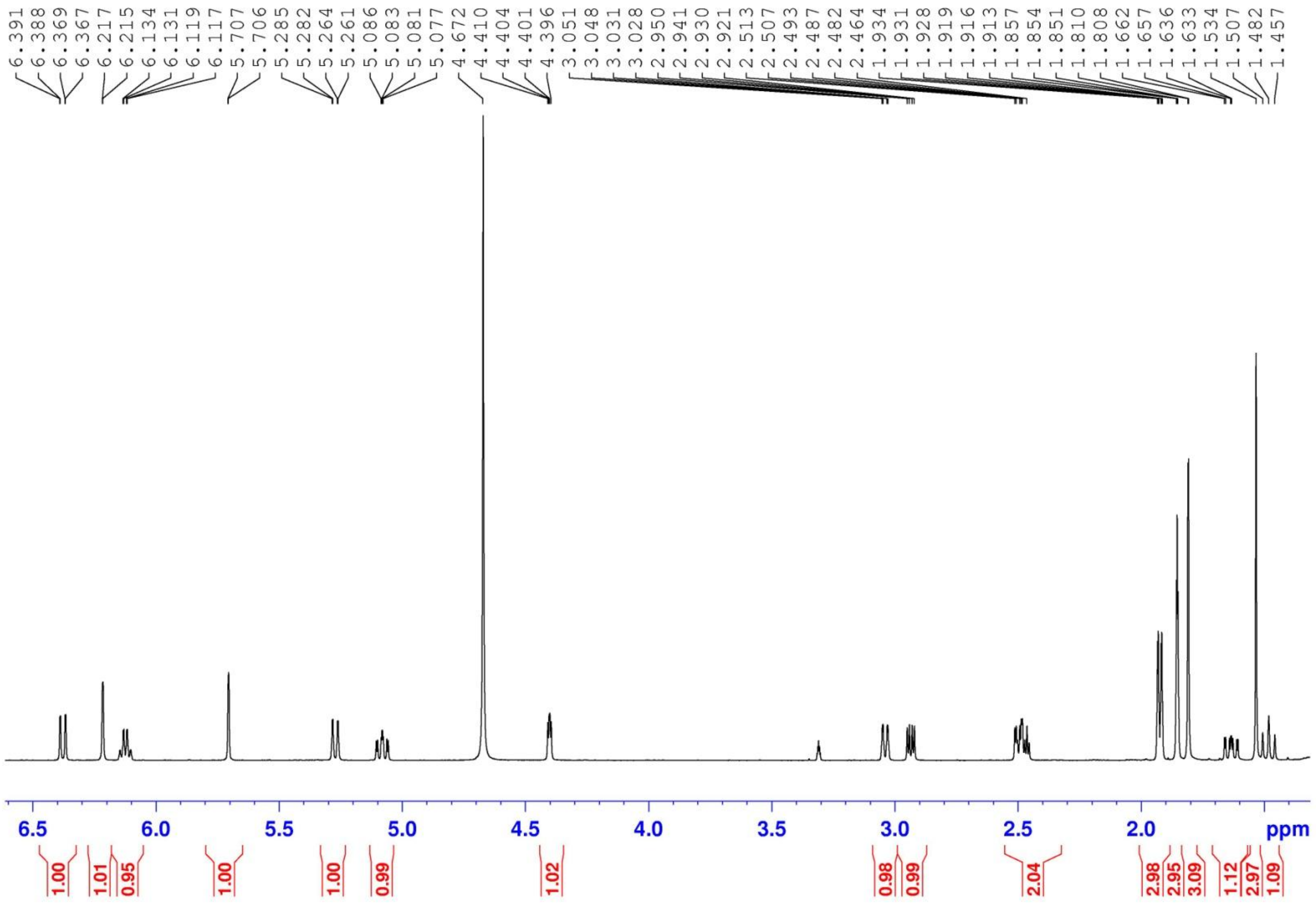

Figure S3. ^{1}H NMR spectrum of compound **2** in CD_3OD.

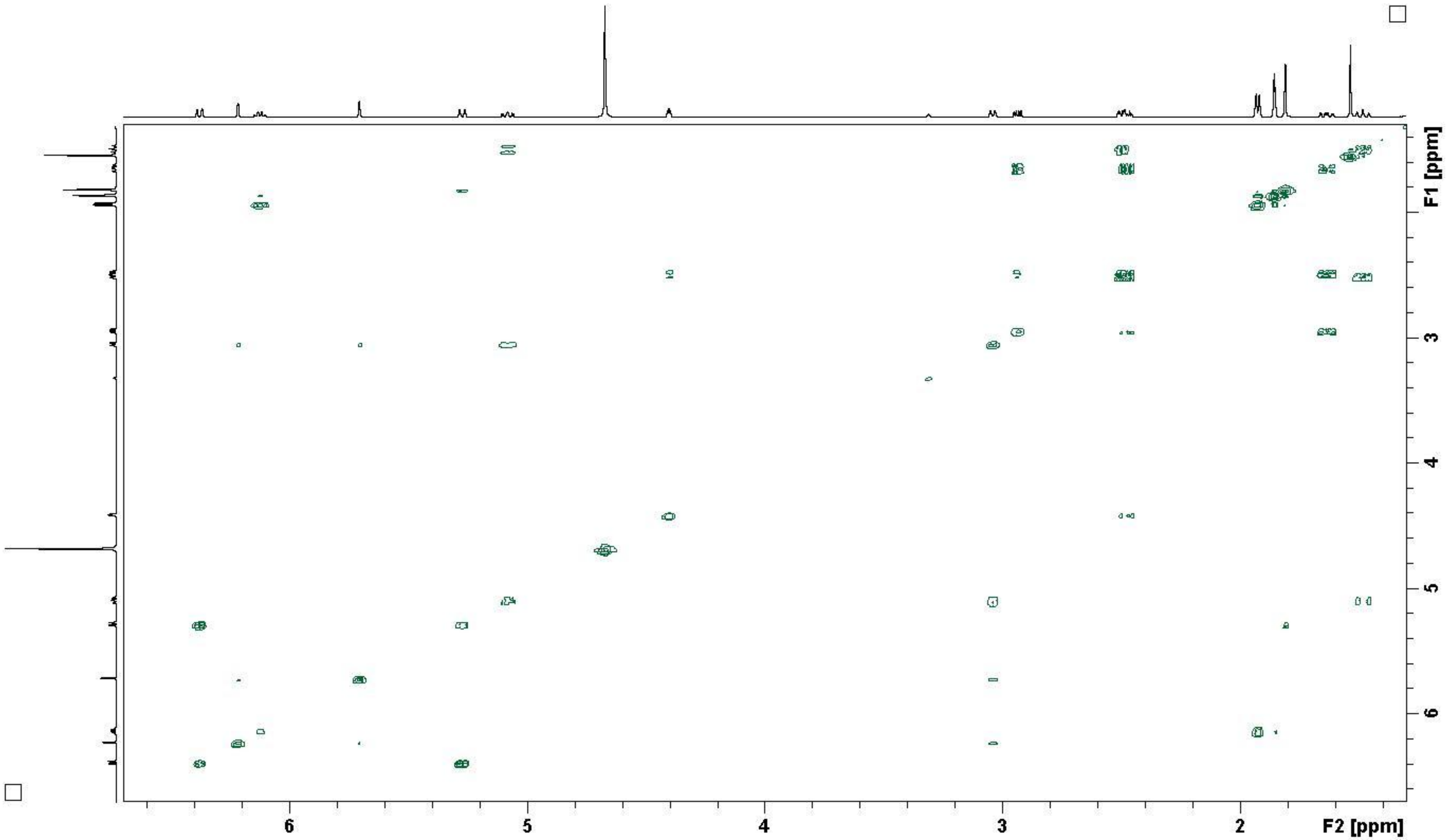

Figure S4. ^1H-^1H-COSY spectrum of compound **2** in CD_3OD.

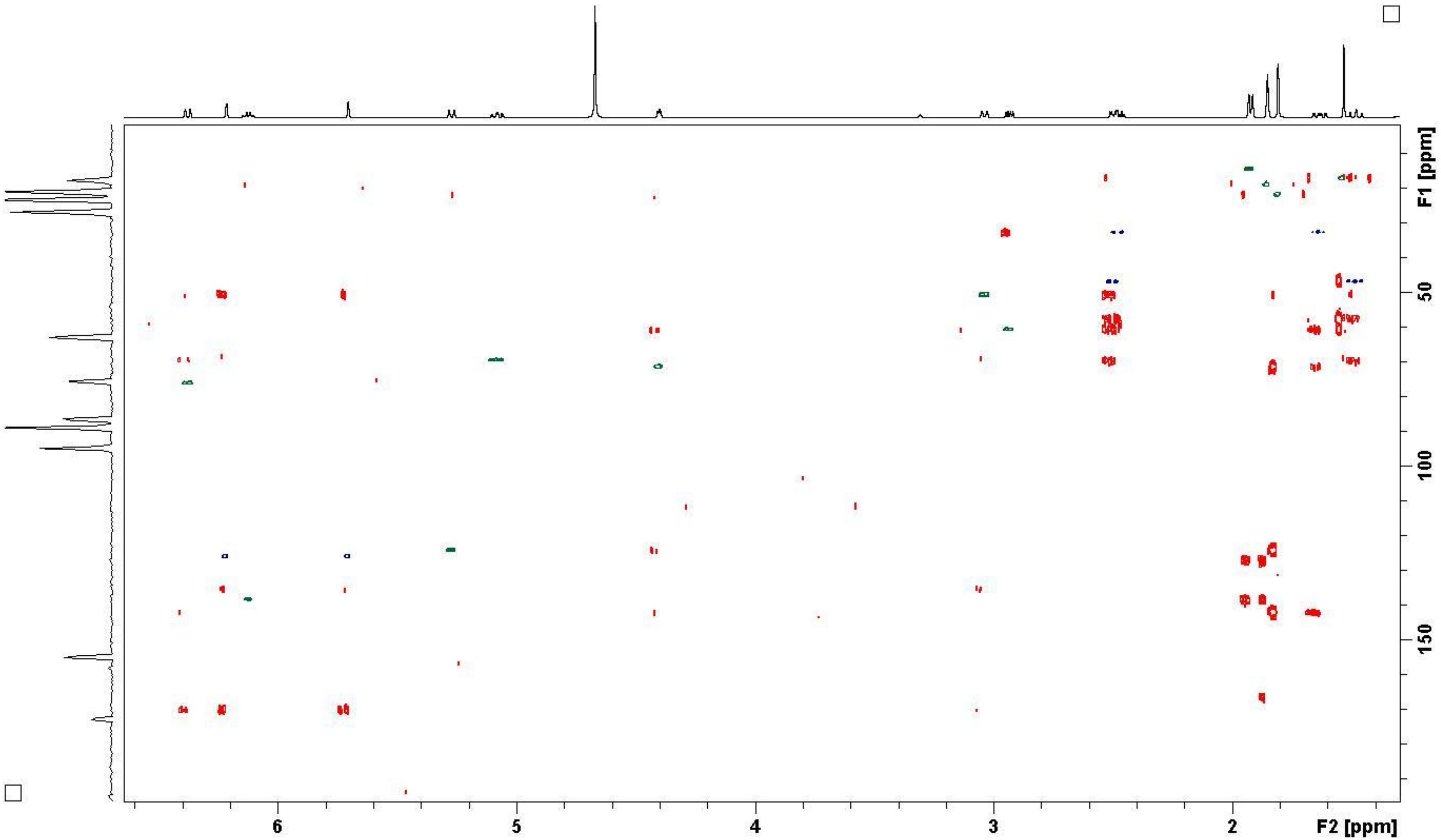

Figure S5. HSQC (green) and HMBC(red) overlaid spectra of compound **2** in CD_3OD.

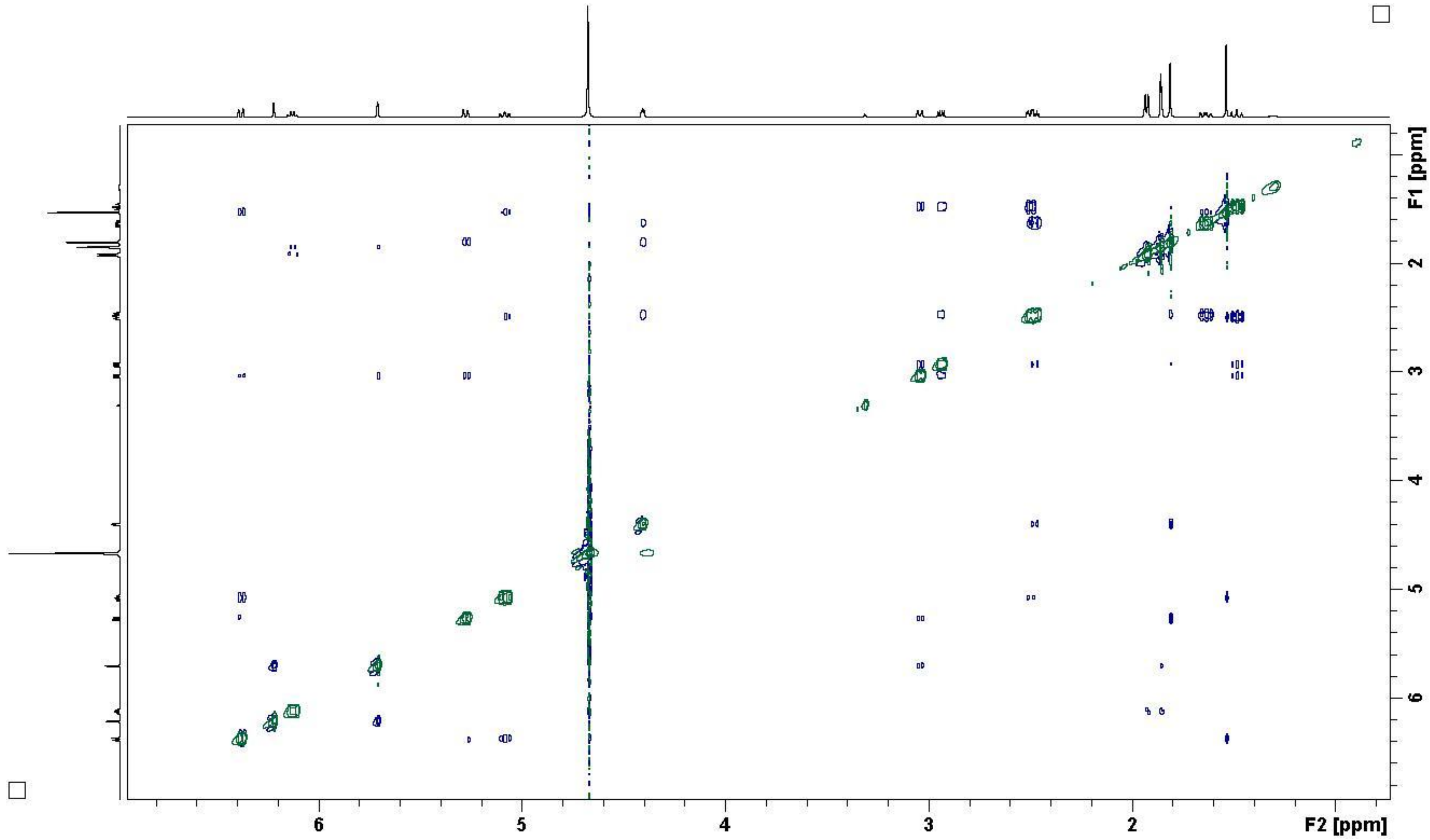

Figure S6: 2D ^1H-^1H NOESY spectrum of compound **2** in CD_3OD.

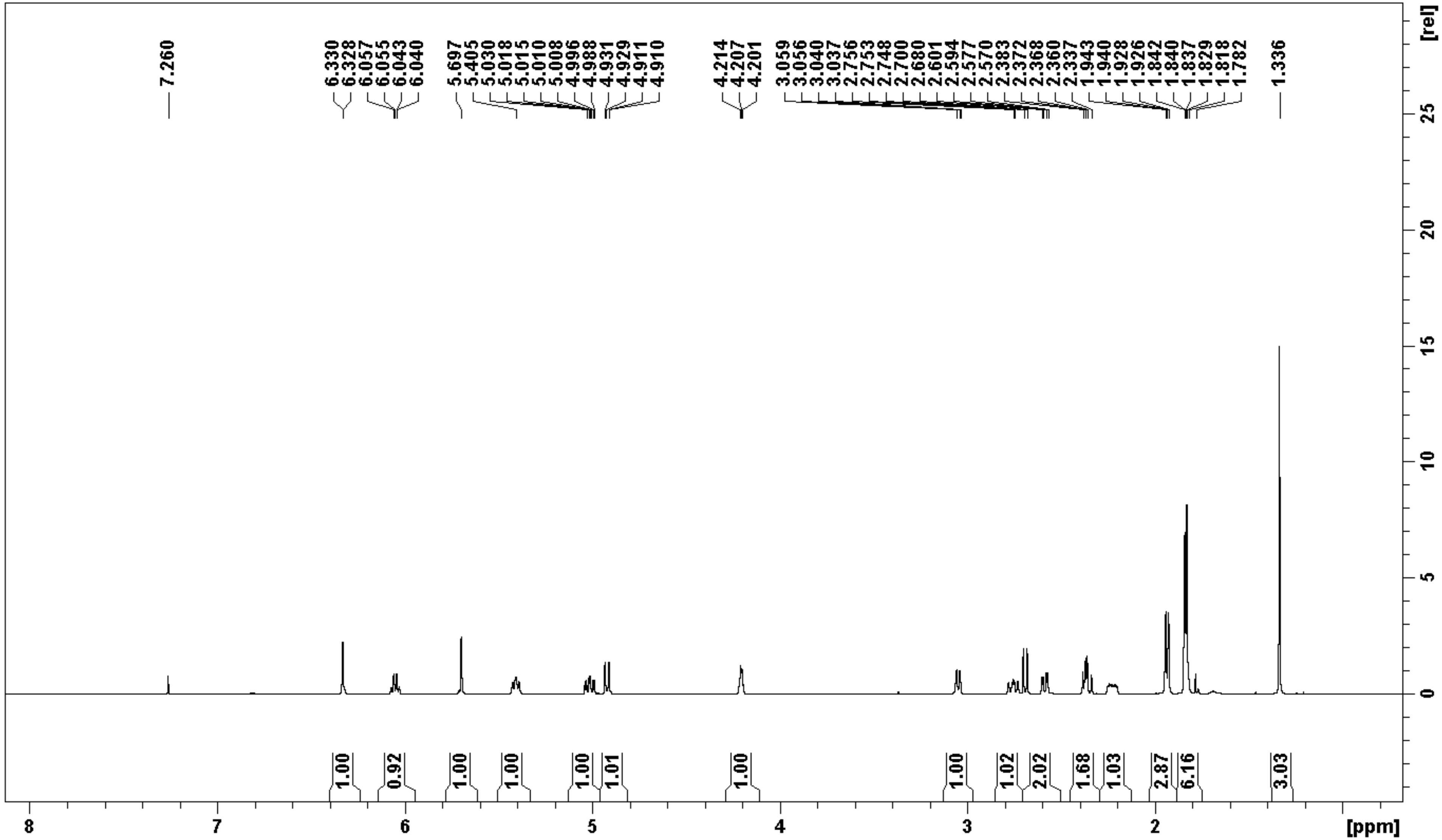

Figure S7. ^{1}H NMR spectrum of compound **3** in $CDCl_3$.

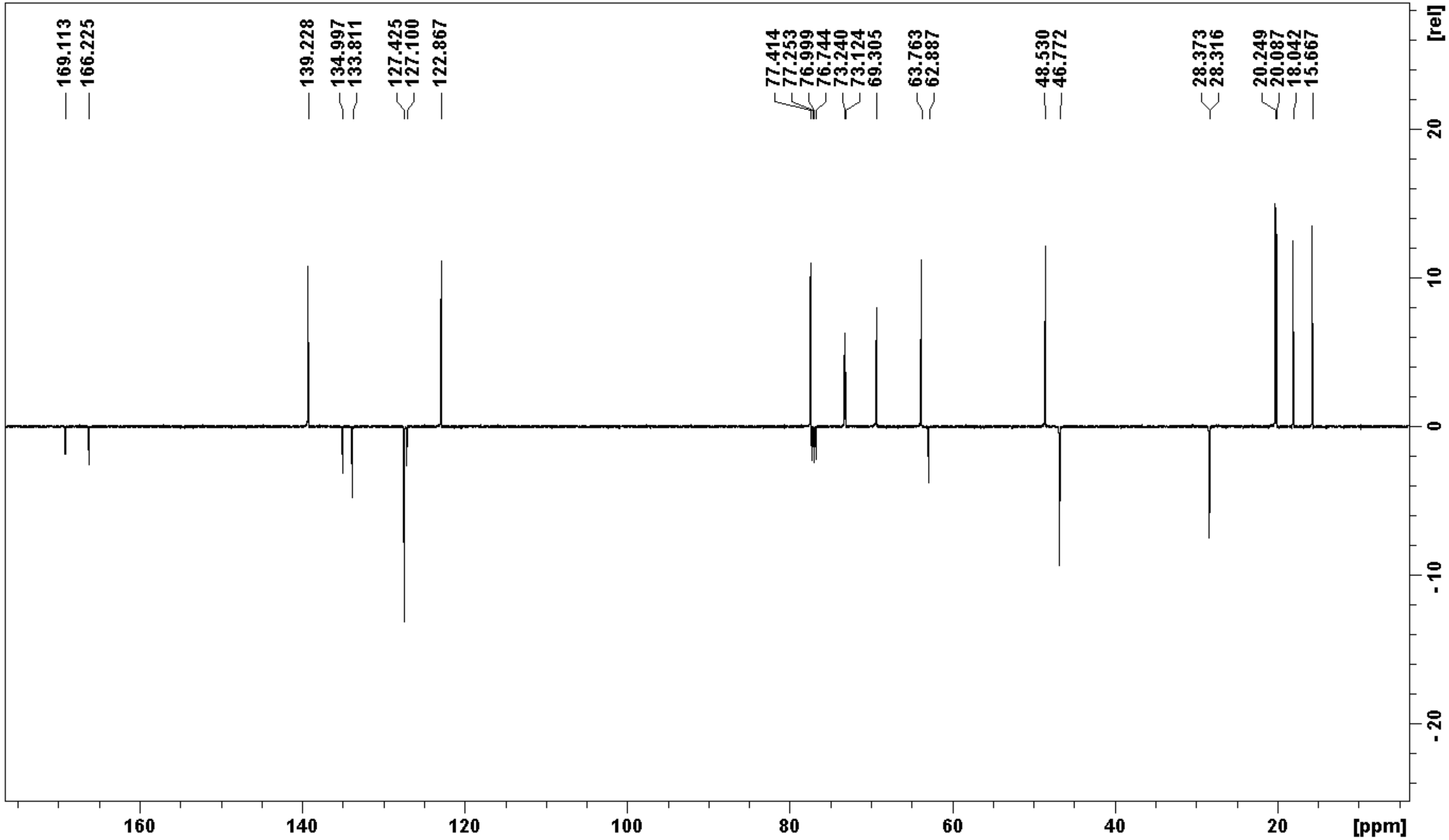

Figure S8: ^{13}C spectrum of compound **3** in $CDCl_3$.

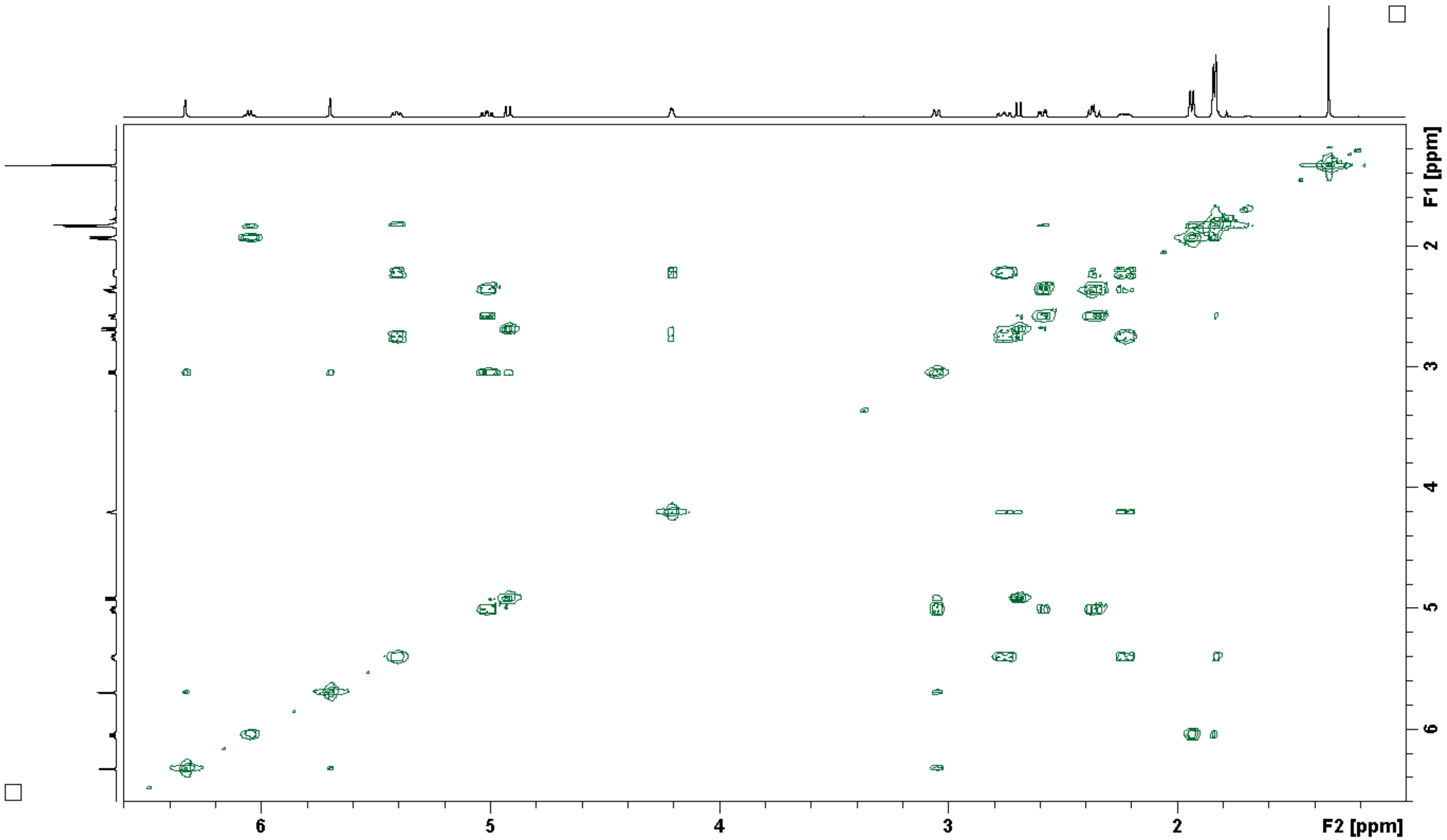

Figure S9. ^{1}H-^{1}H-COSY spectrum of compound **3** in $CDCl_3$.

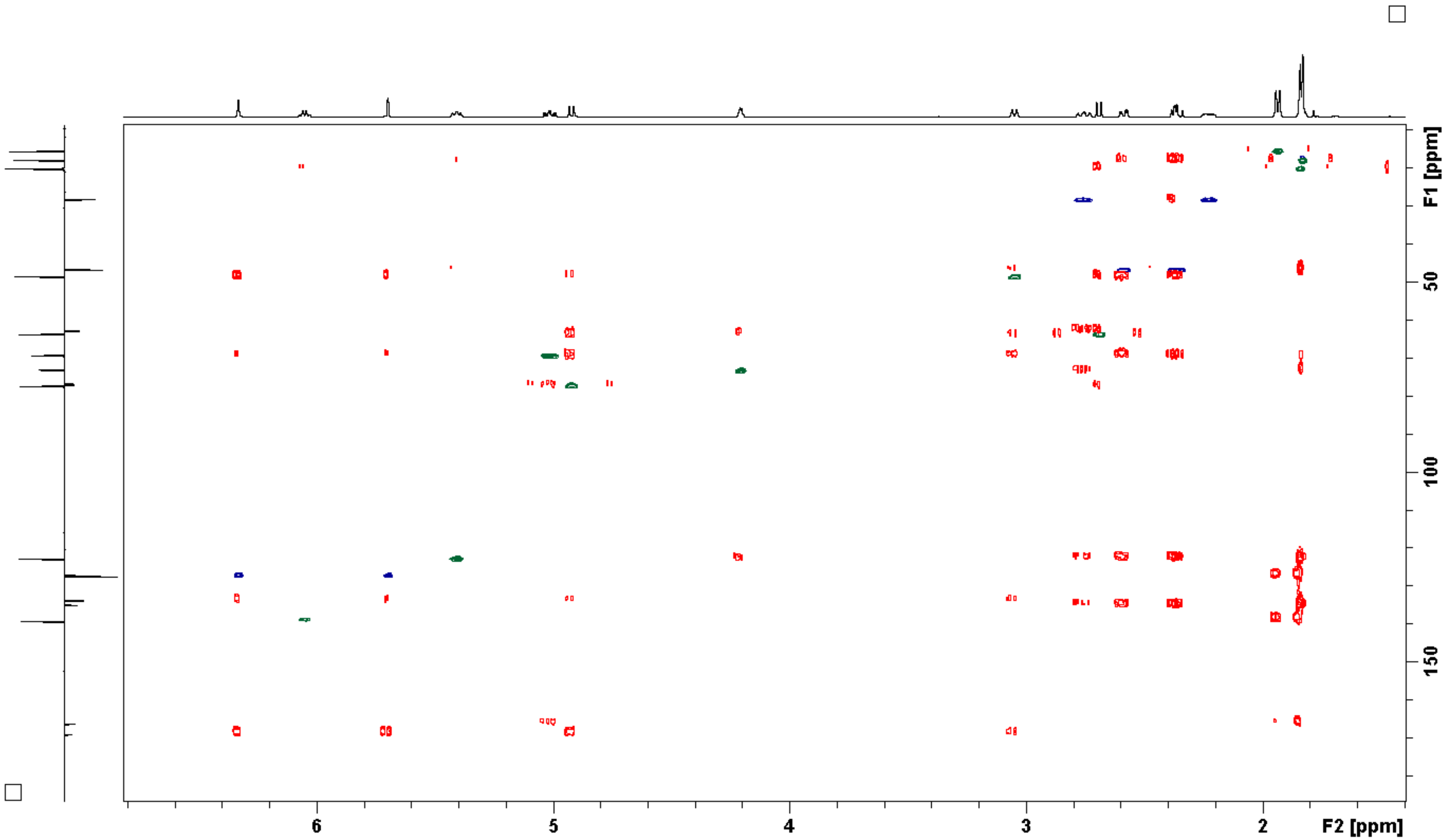

Figure S10. HSQC (green) and HMBC(red) overlaid spectra of compound **3** in $CDCl_3$.

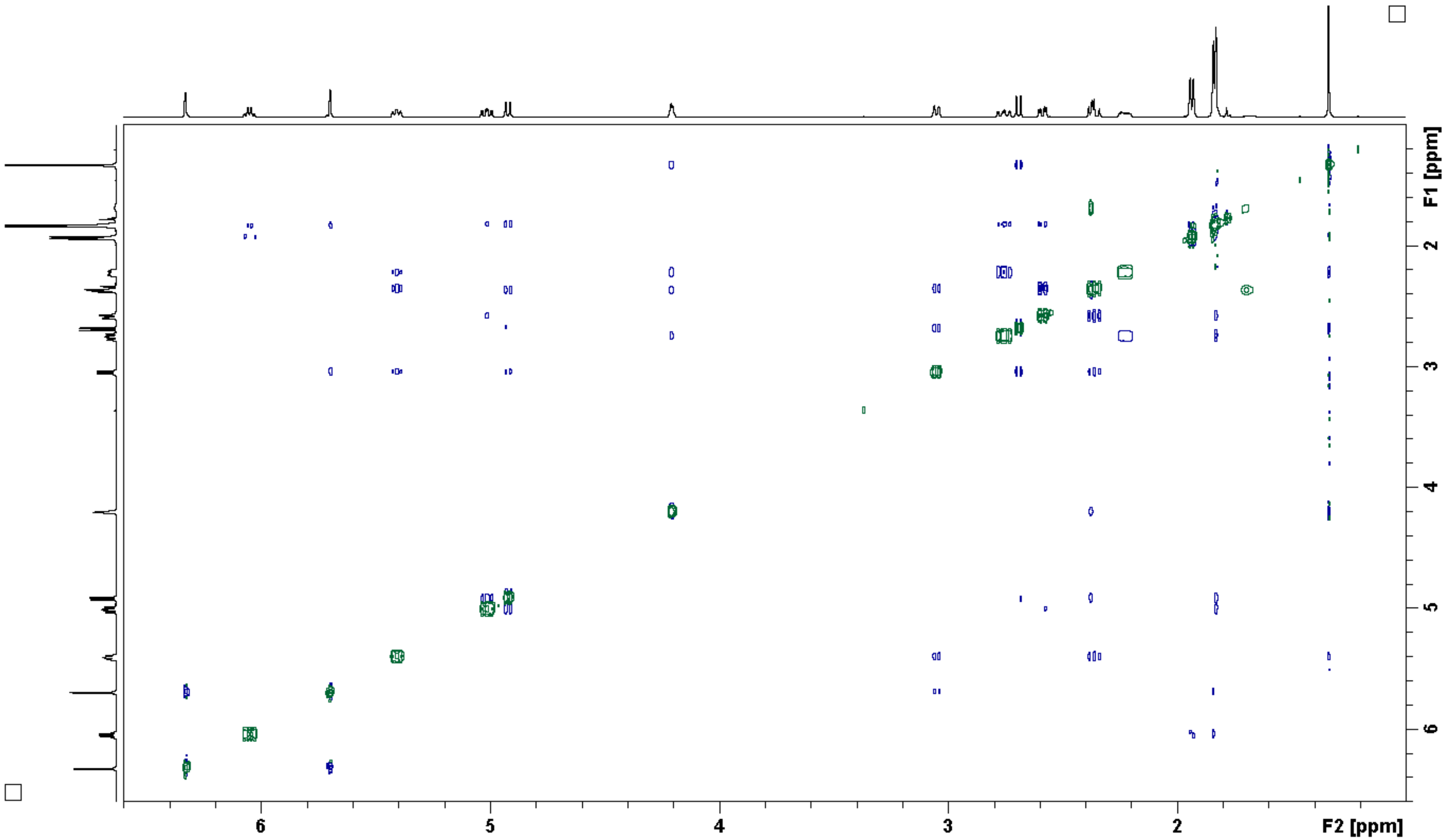

Figure S11: 2D ^{1}H-^{1}H NOESY spectrum of compound **3** in $CDCl_3$.

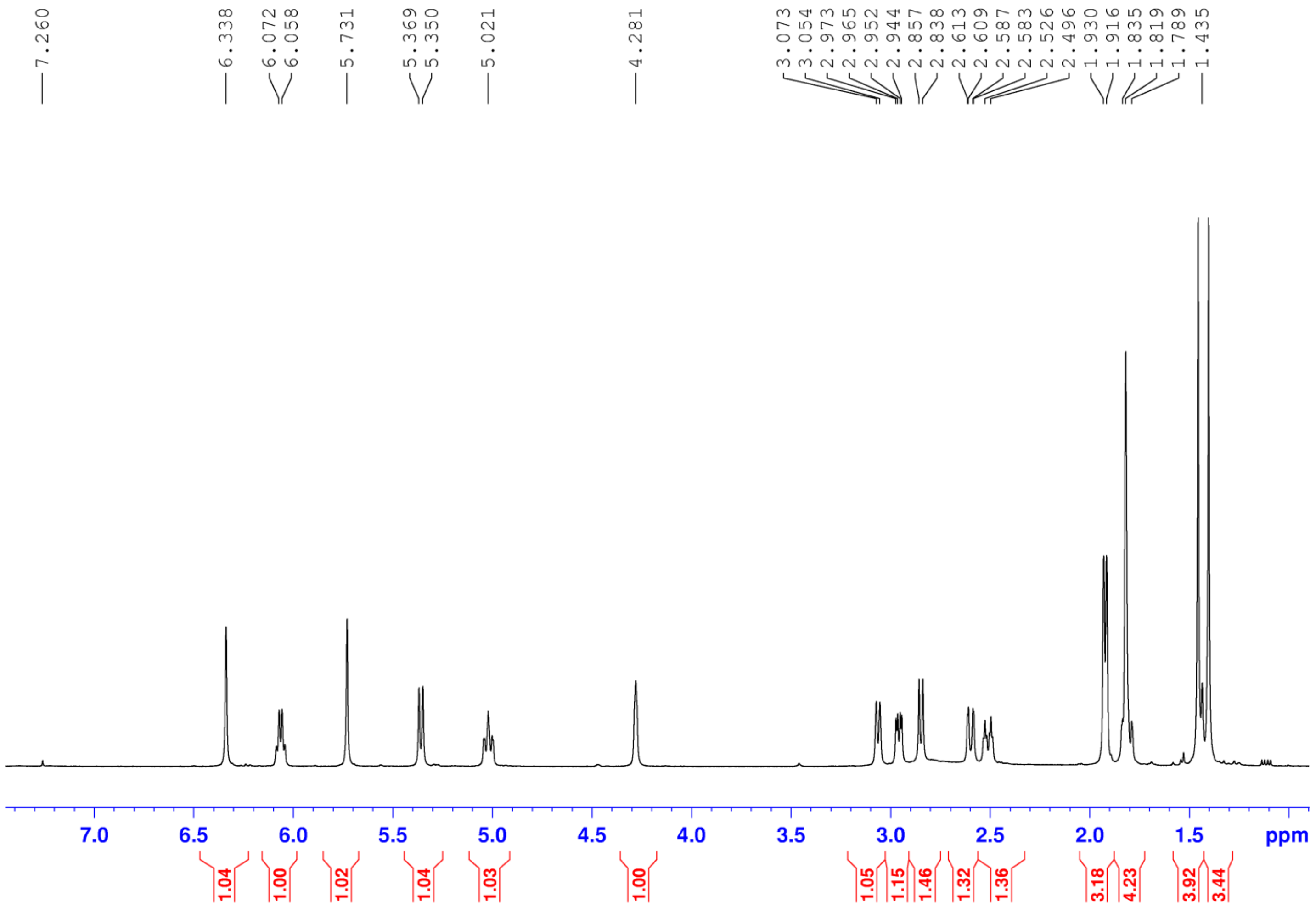

Figure S12. ^{1}H NMR spectrum of compound **4** in $CDCl_3$.

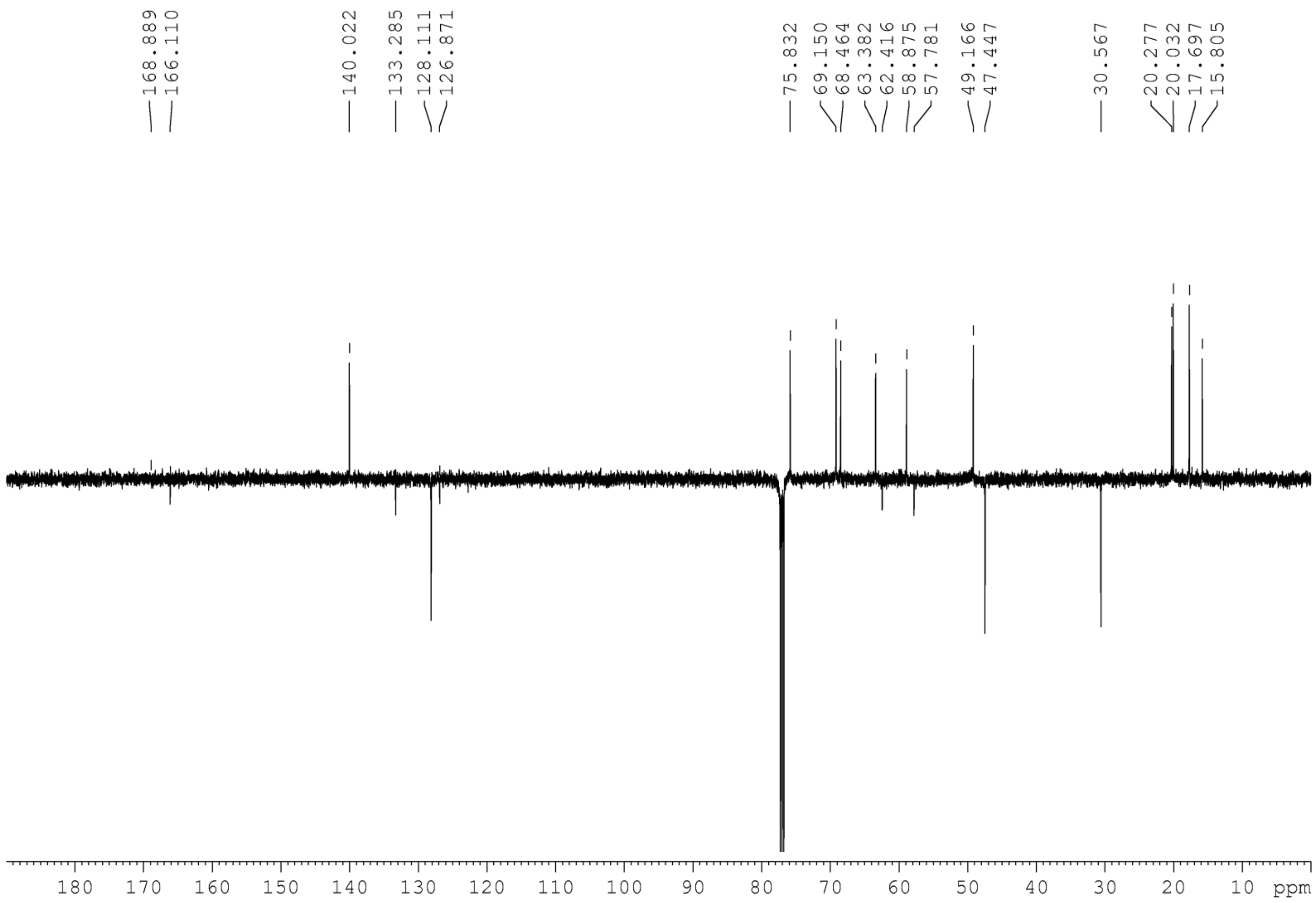

Figure S13: ^{13}C spectrum of compound **4** in $CDCl_3$.

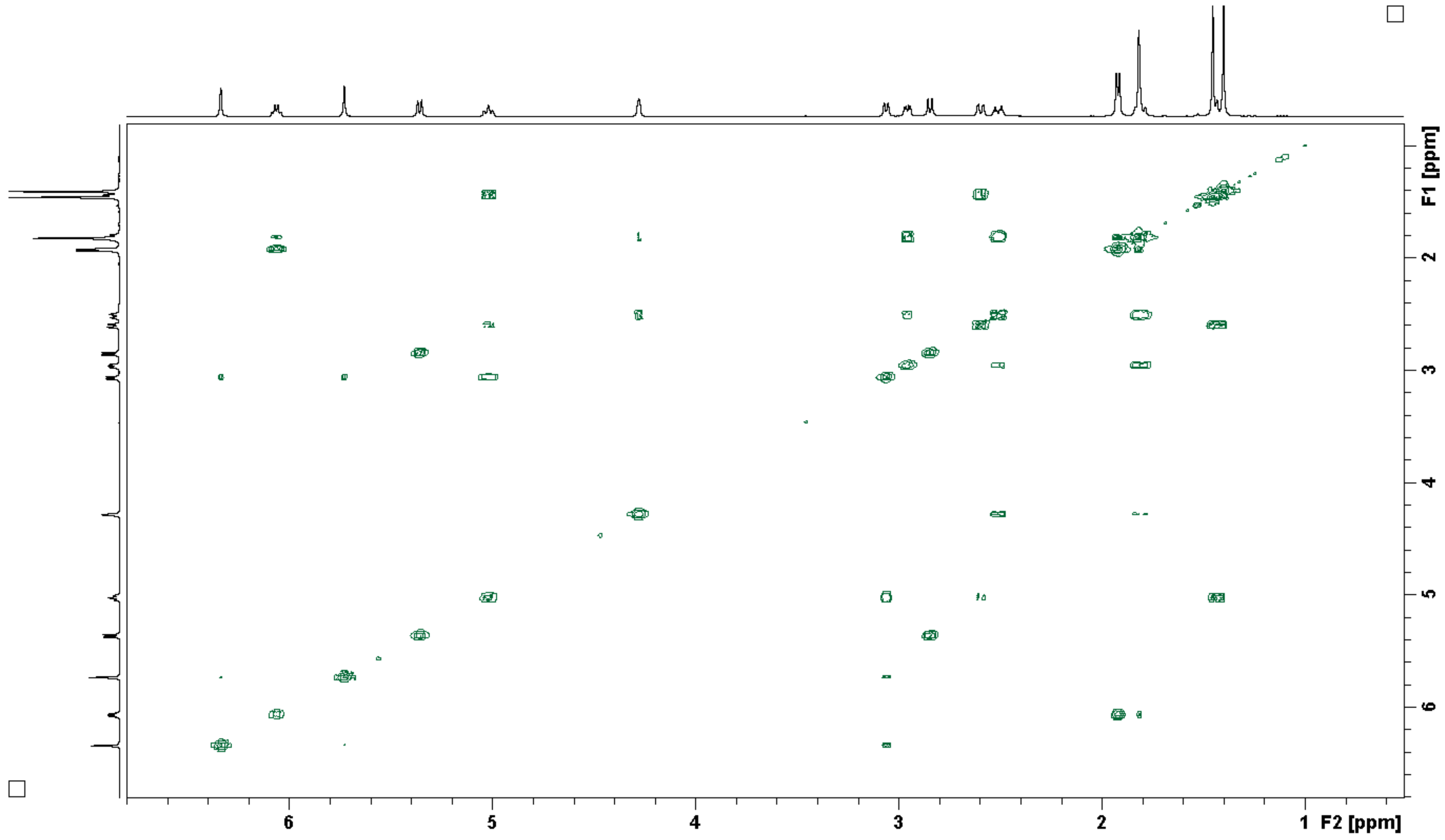

Figure S14. ¹H-¹H-COSY spectrum of compound **4** in $CDCl_3$.

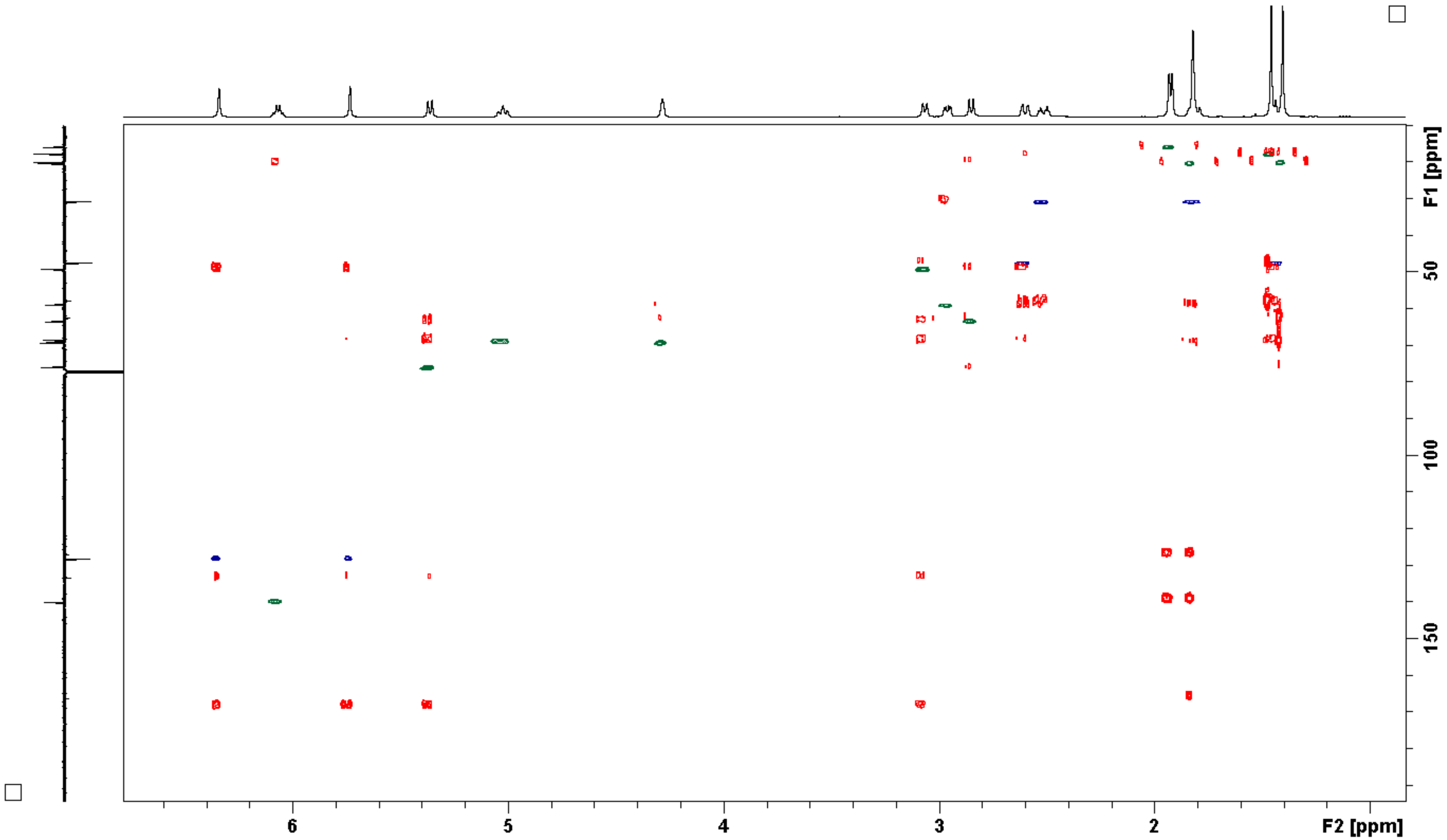

Figure S15. HSQC (green) and HMBC(red) overlaid spectra of compound **4** in $CDCl_3$.

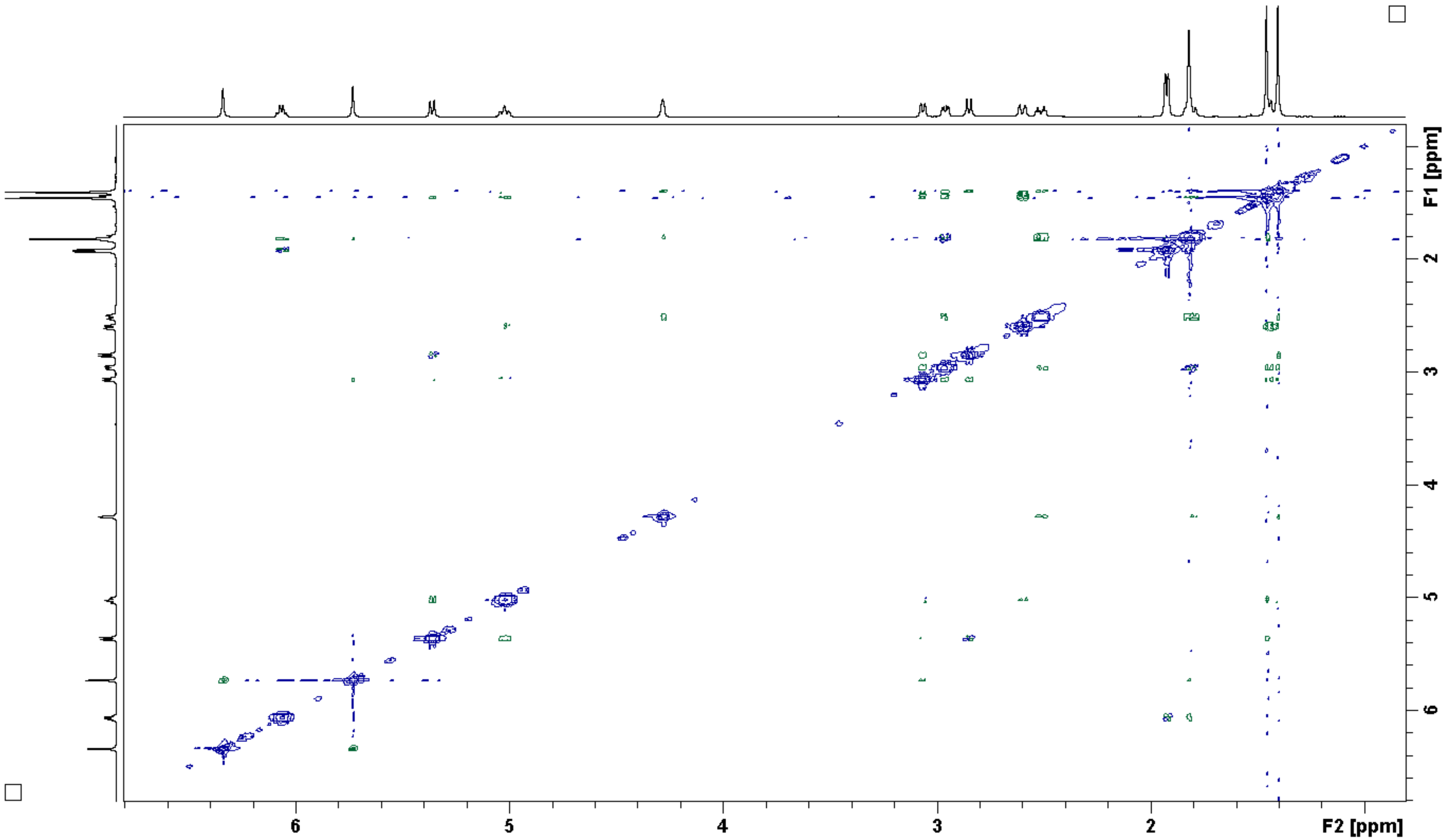

Figure S16: 2D ^{1}H-^{1}H NOESY spectrum of compound **4** in $CDCl_3$.

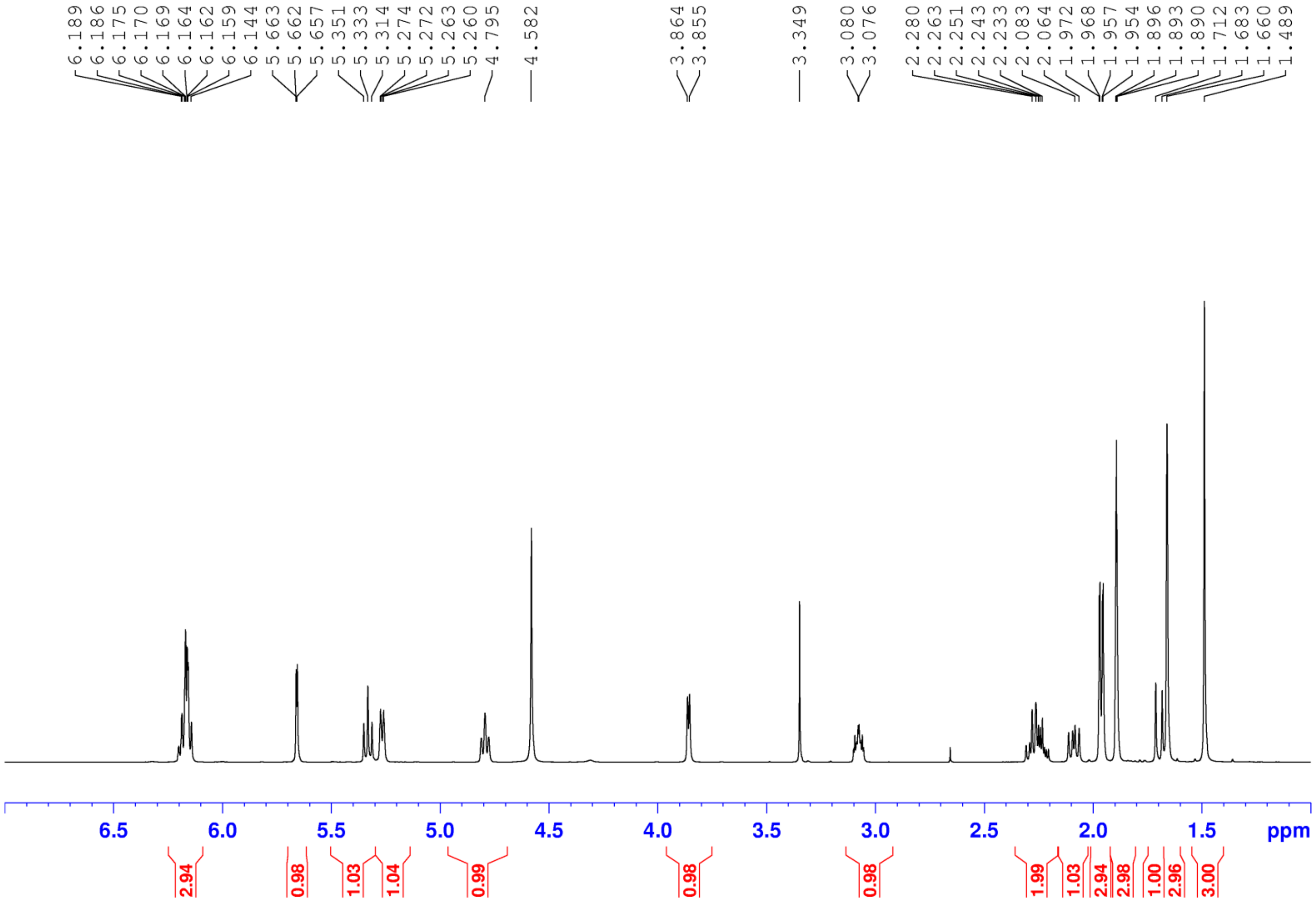

Figure S17: ^{1}H-NMR spectrum of compound **5** in CD_3OD.

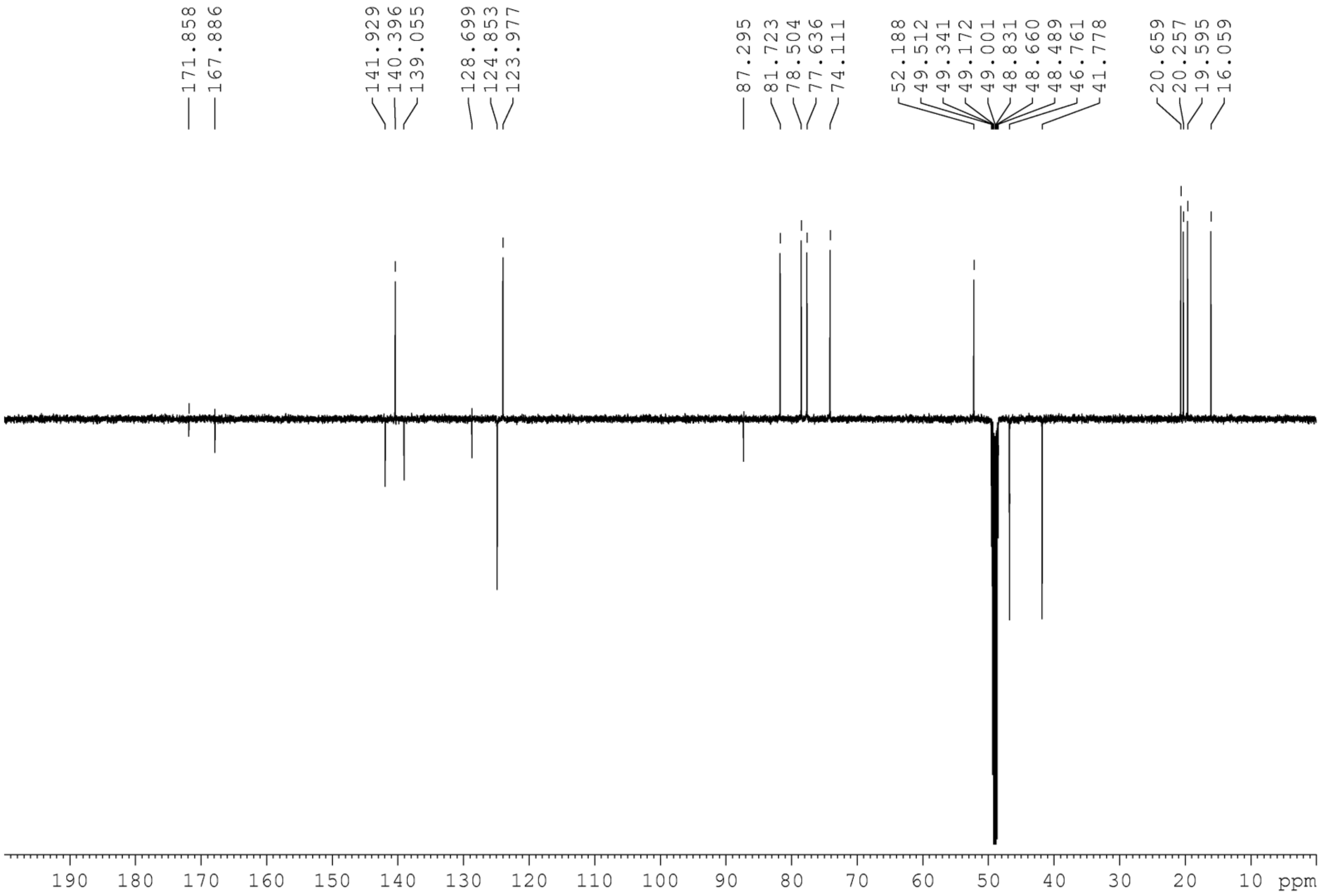

Figure S18: ^{13}C spectrum of compound **5** in CD_3OD.

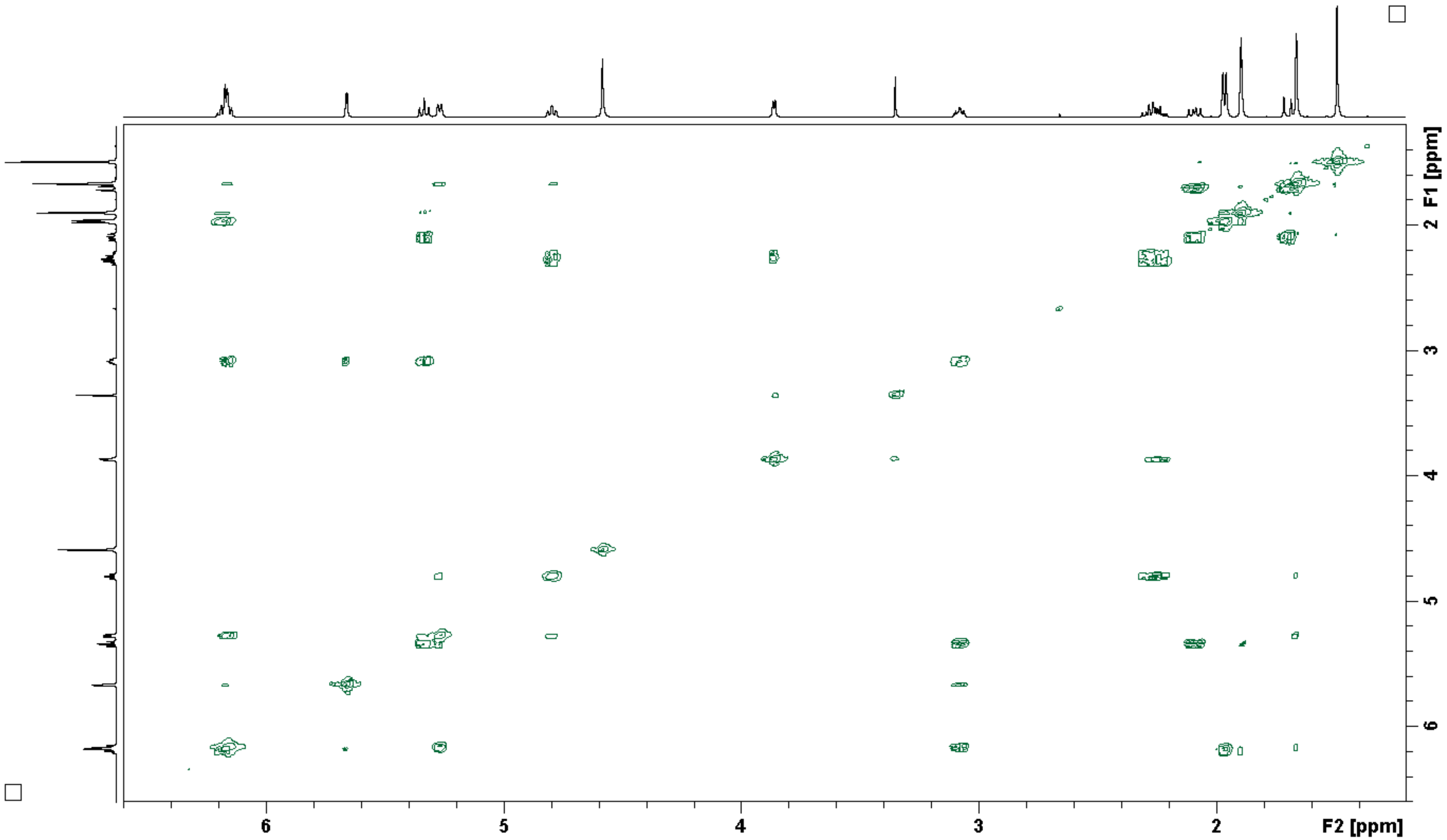

Figure S19: ¹H-¹H COSY spectrum of compound **5** in CD_3OD.

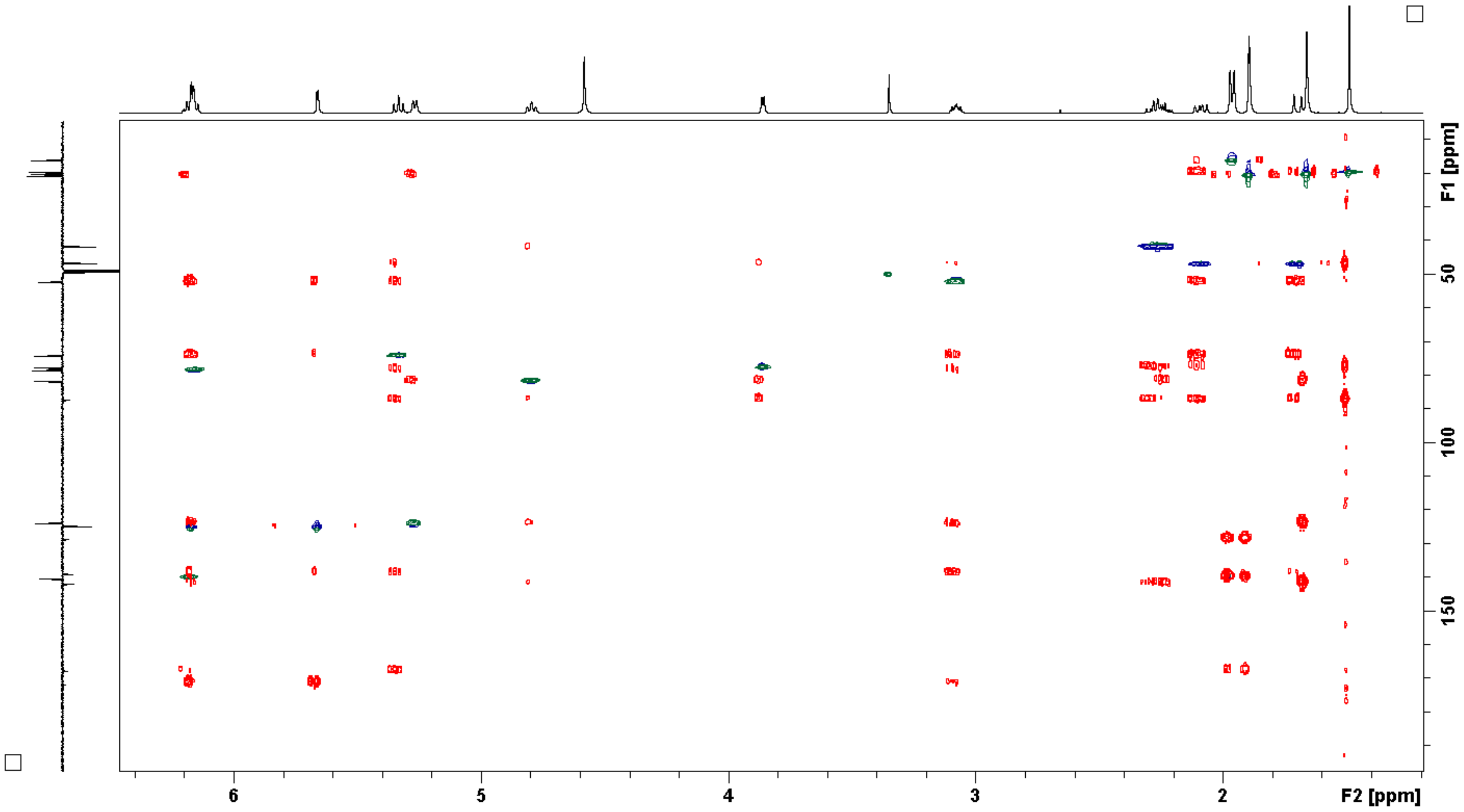

Figure S20: Overlay of HSQC and HMBC spectra of compound **5** in CD_3OD.

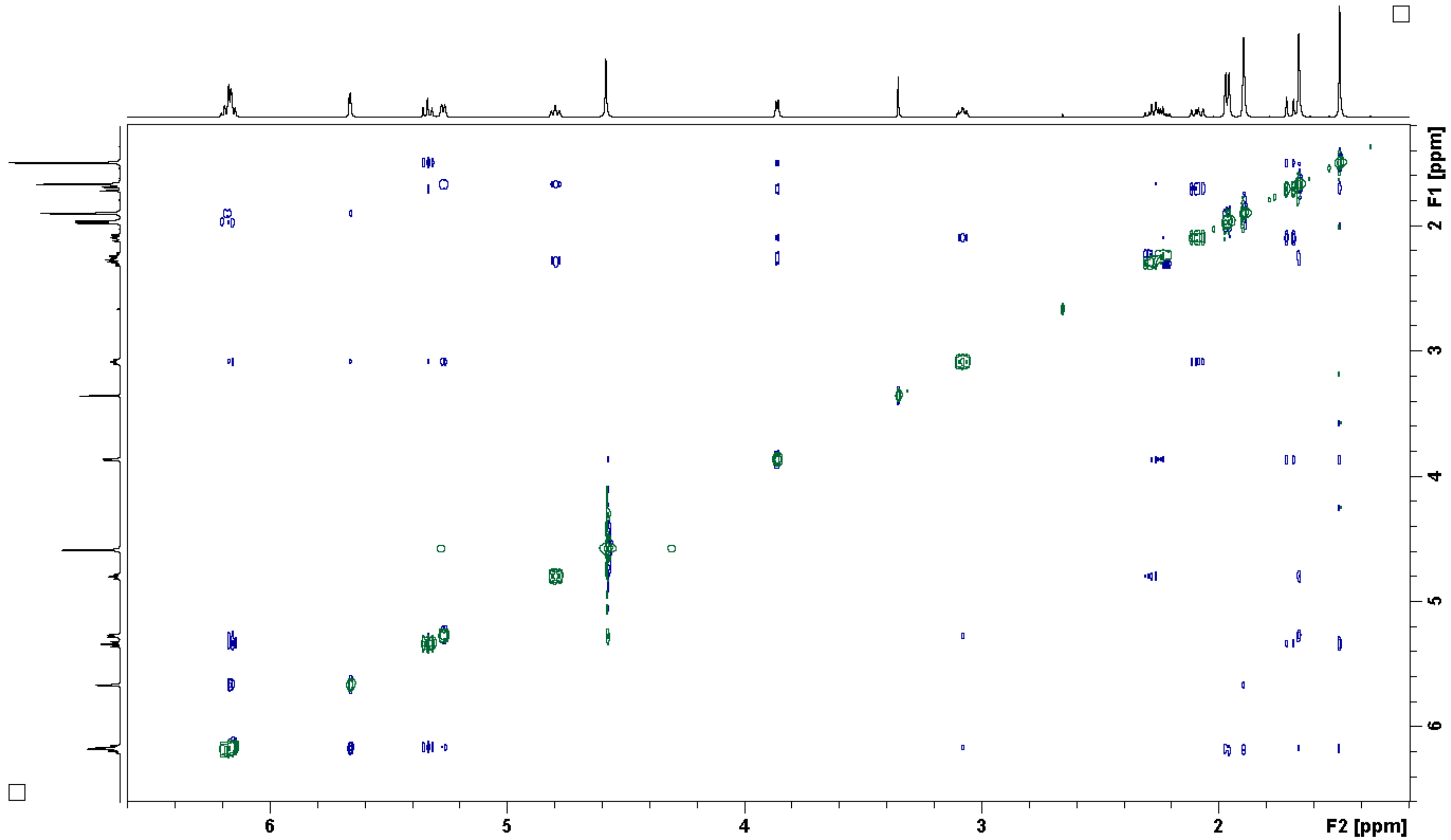

Figure S21: 2D ^{1}H-^{1}H NOESY spectrum of compound **5** in CD_3OD.

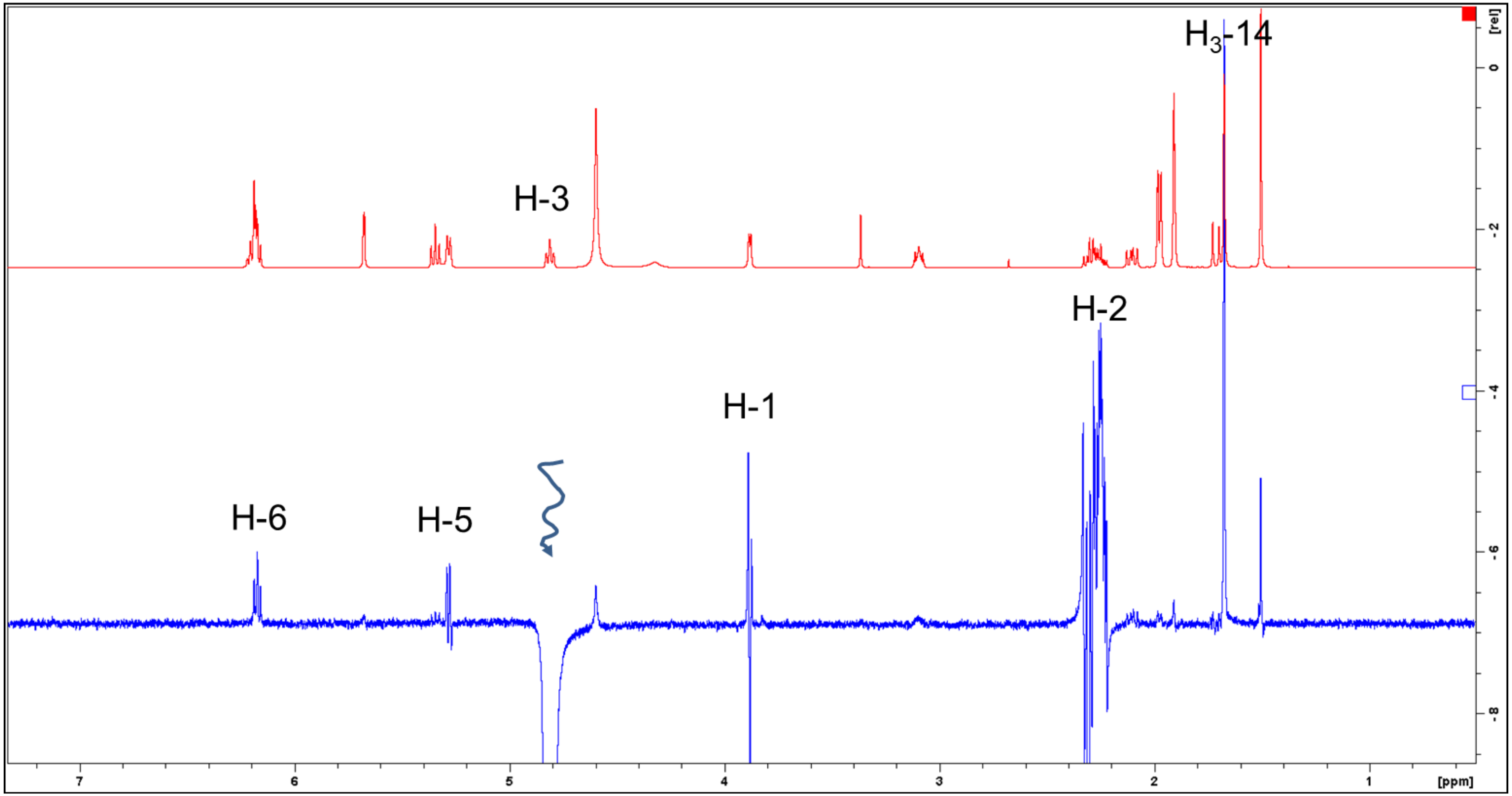

Figure S22: 1D Selective NOESY spectrum of compound **5** in CD_3OD (D8 = 0.3 sec).

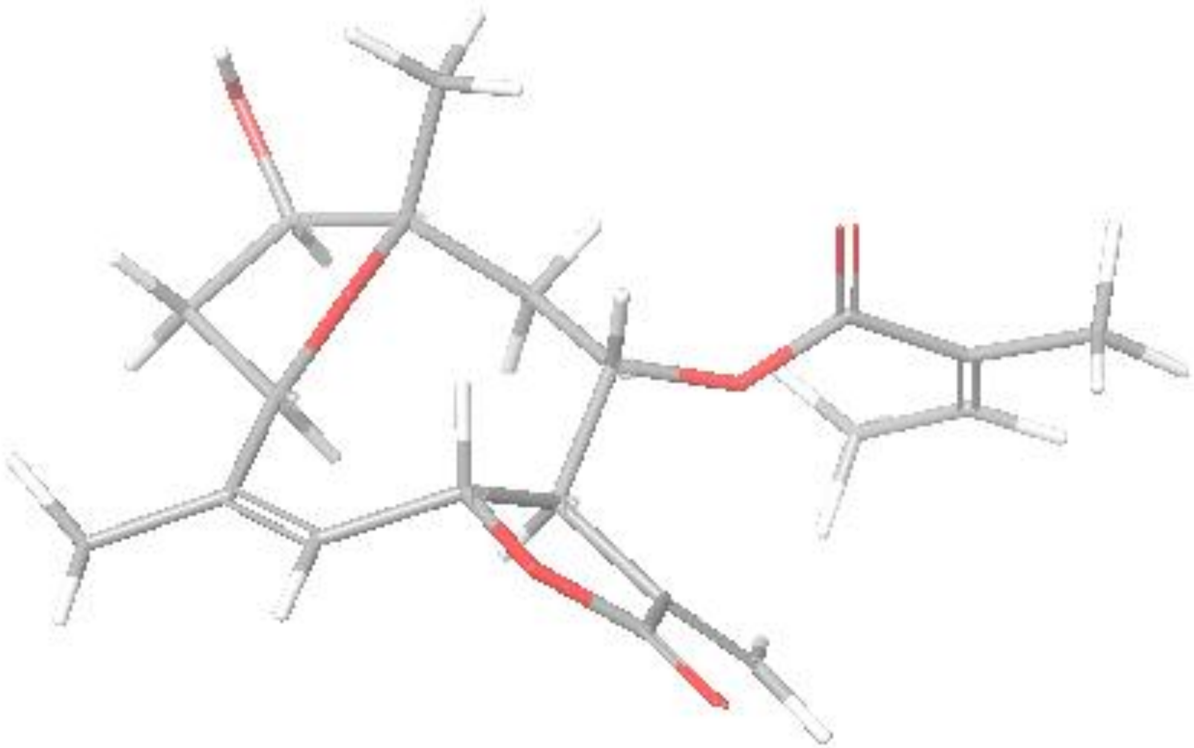

Figure S23: DFT 6-31+G(d,p) optimized structure of **5**.

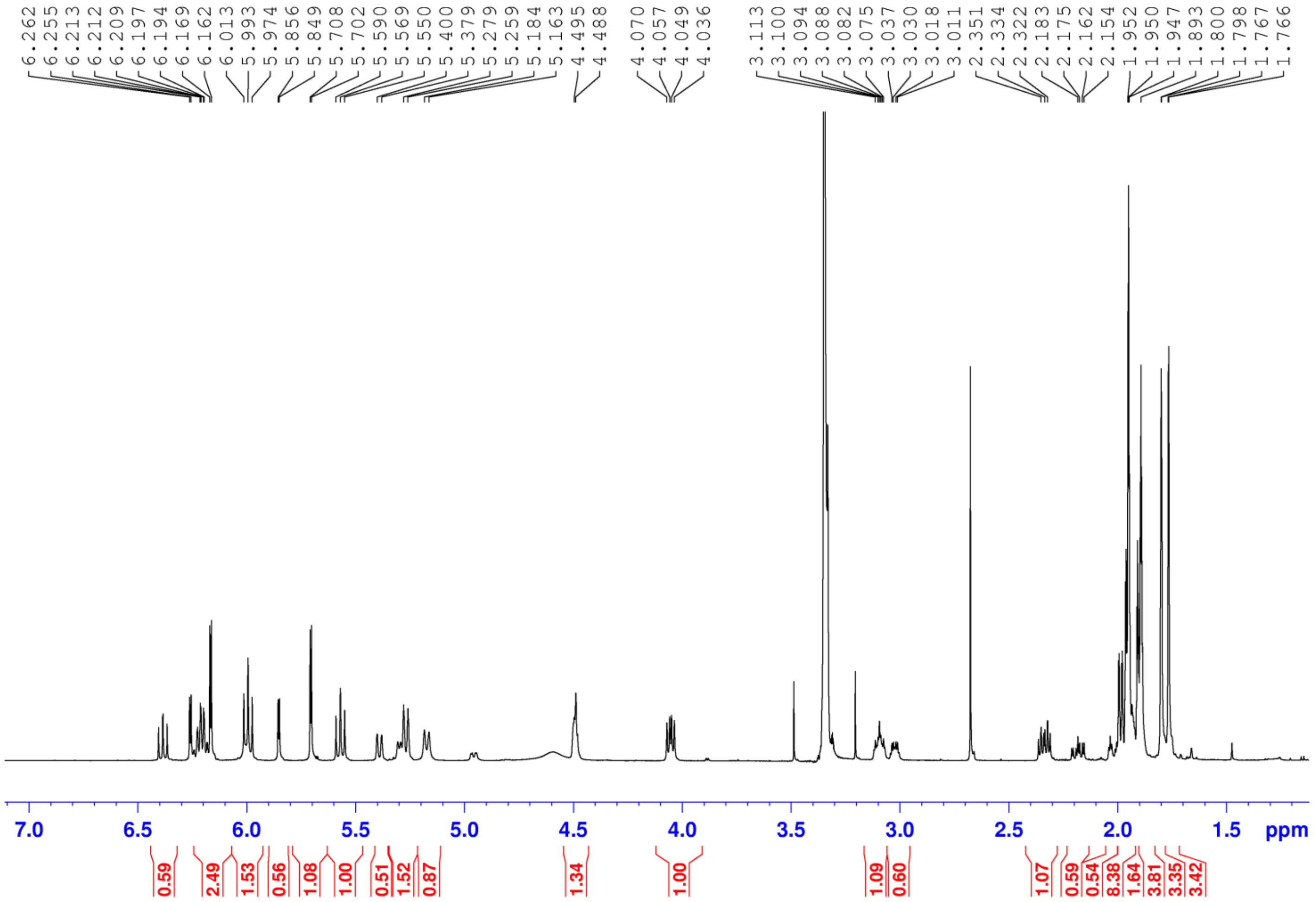

Figure S24: ^{1}H-NMR spectrum of compounds **6-7** in CD_3OD.

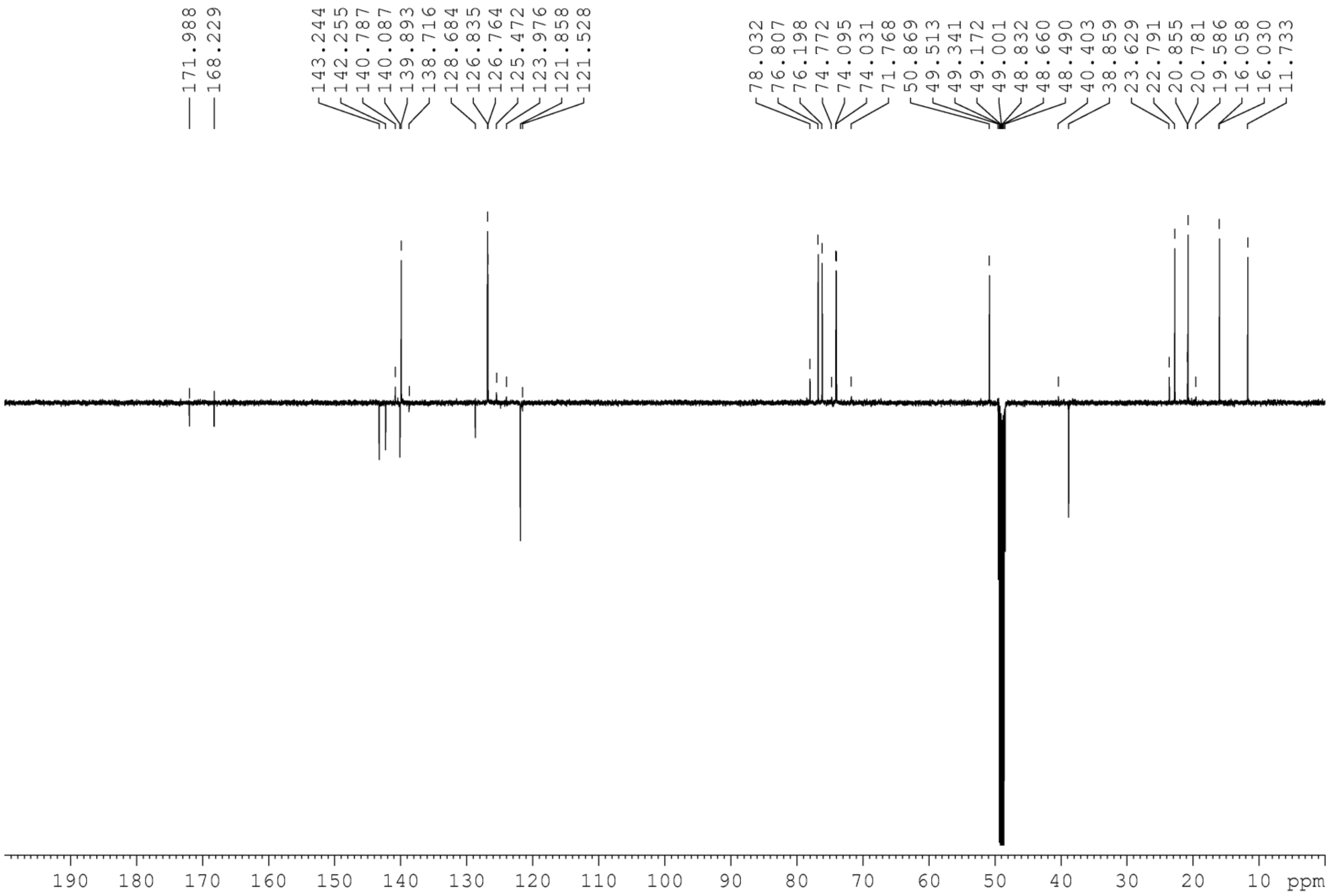

Figure S25: ^{13}C spectrum of compounds **6**-**7** in CD_3OD.

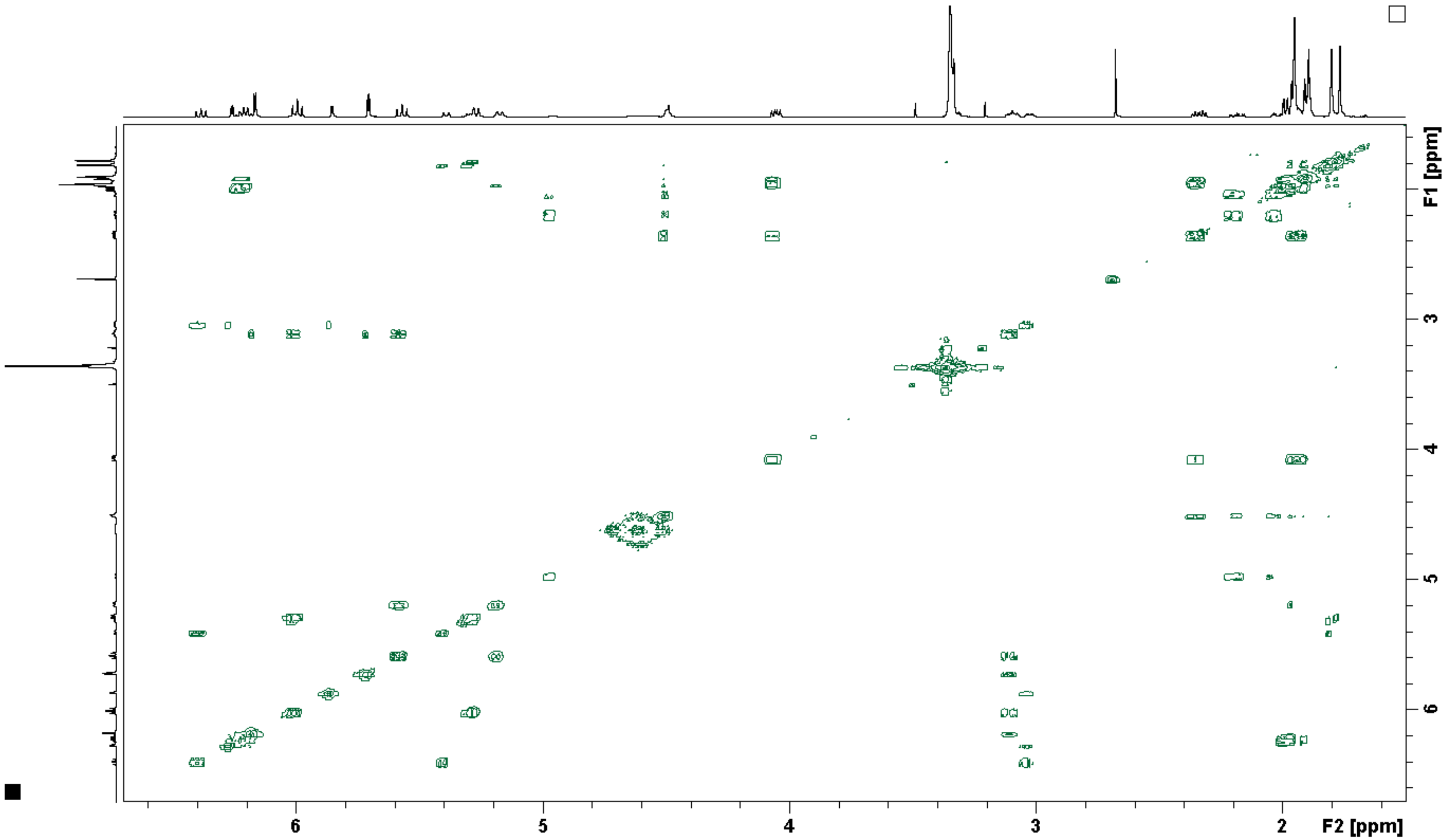

Figure S26: ^{1}H-^{1}H COSY spectrum of compounds **6-7** in CD_3OD.

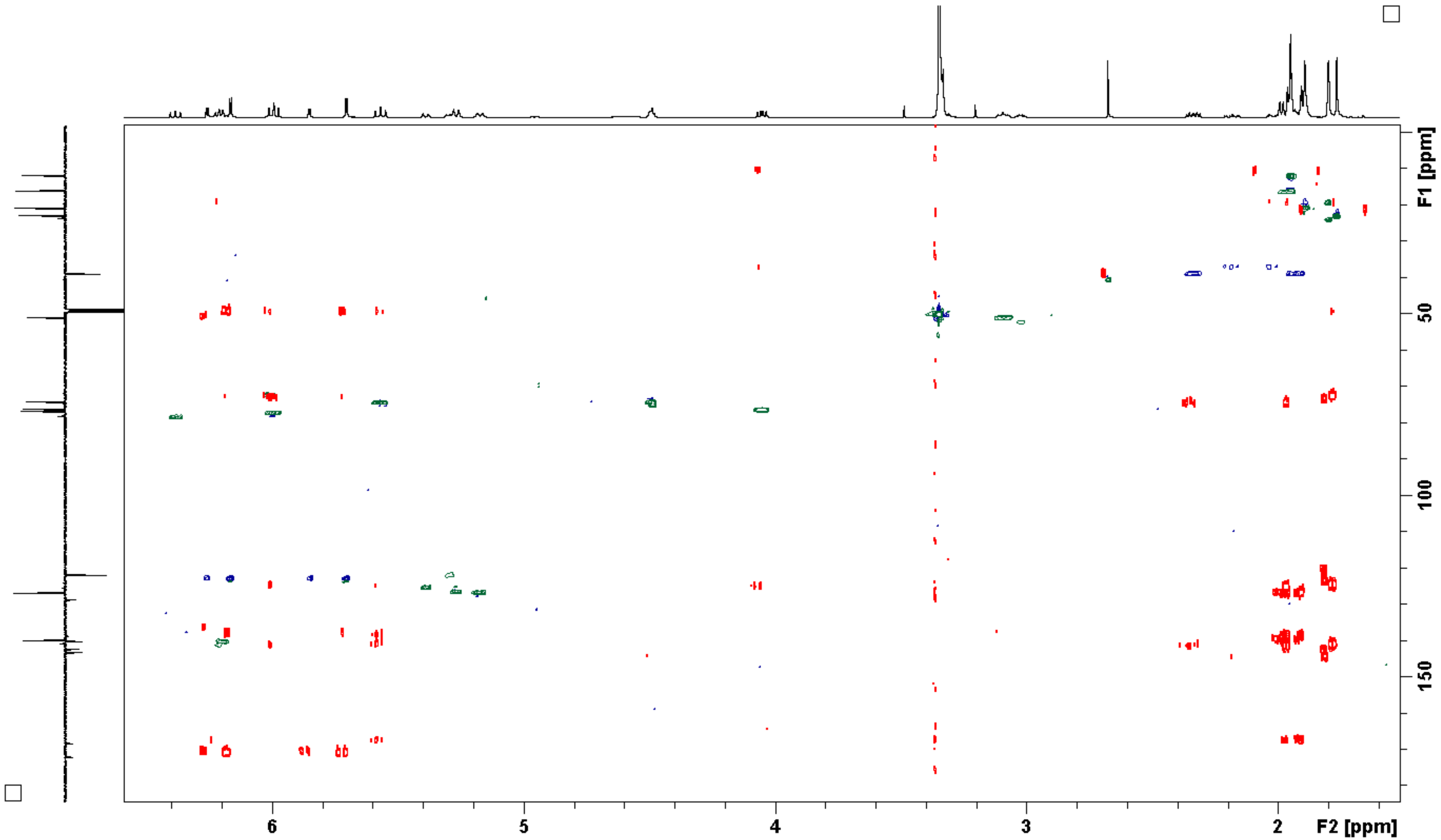

Figure S27: Overlay of HSQC and HMBC spectra of compounds **6**-**7** in CD_3OD.

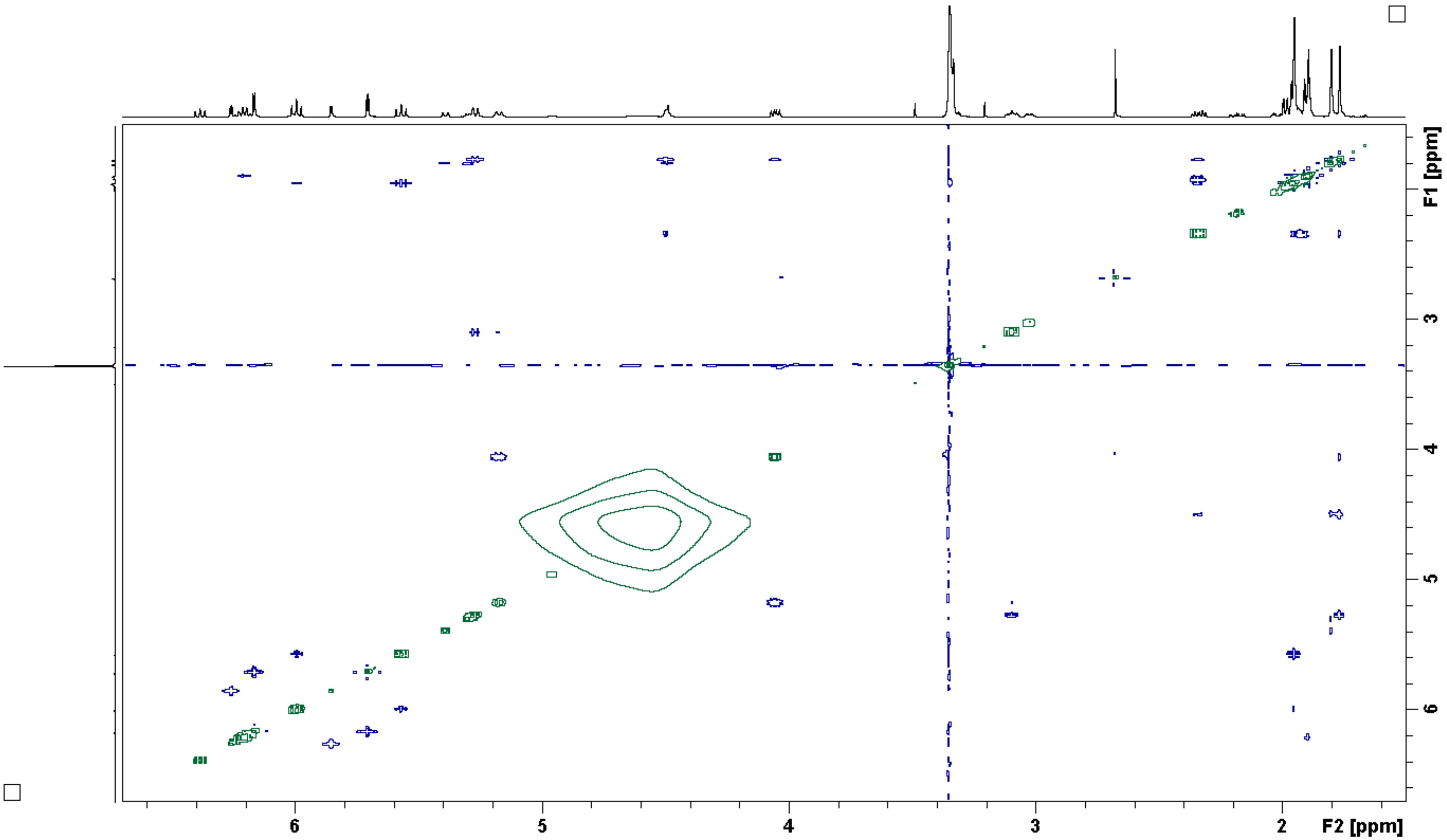

Figure S28: 2D ^1H-^1H NOESY spectrum of compounds **6-7** in CD_3OD.

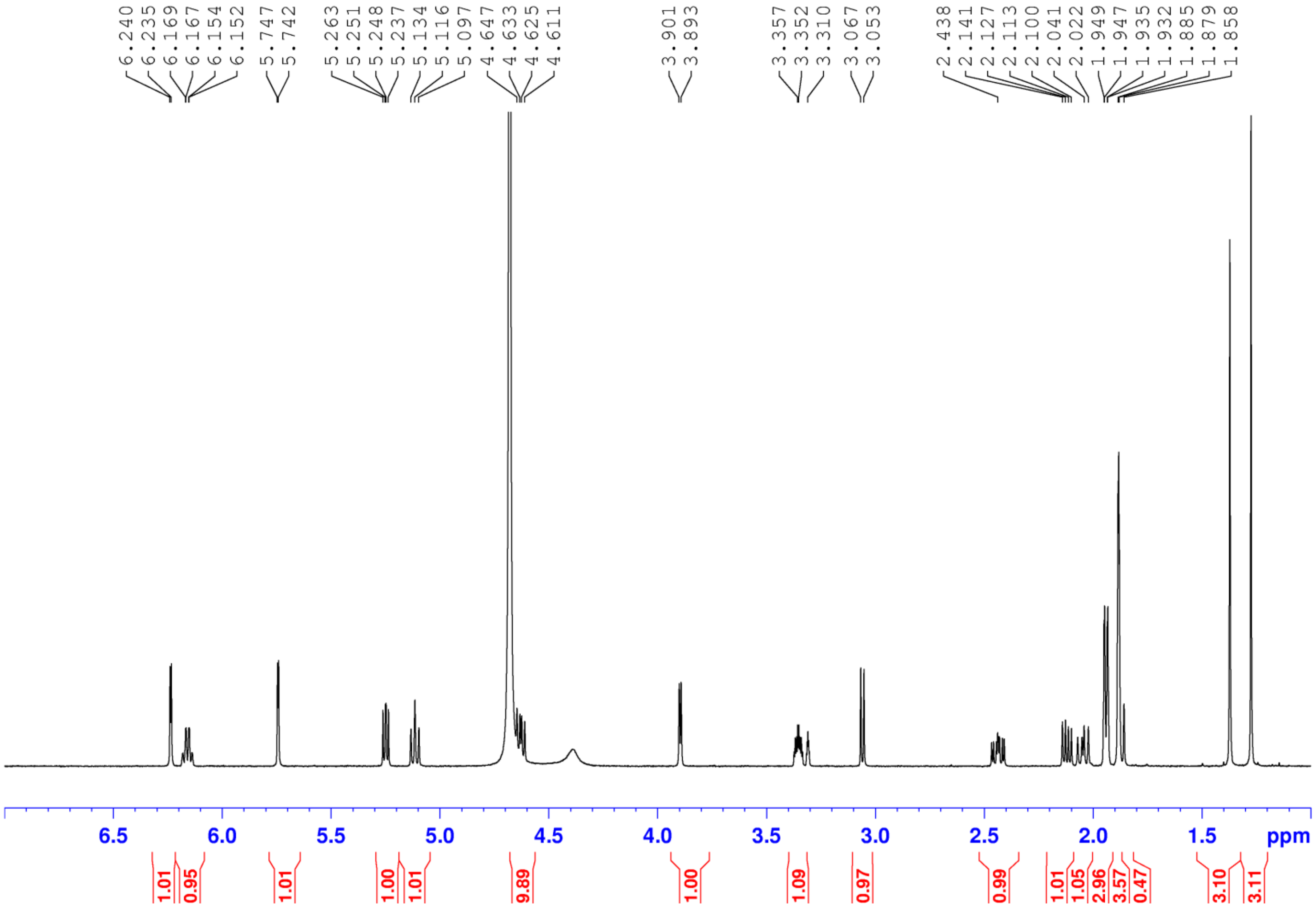

Figure S29: ^{1}H-NMR spectrum of compound **8** in CD_3OD.

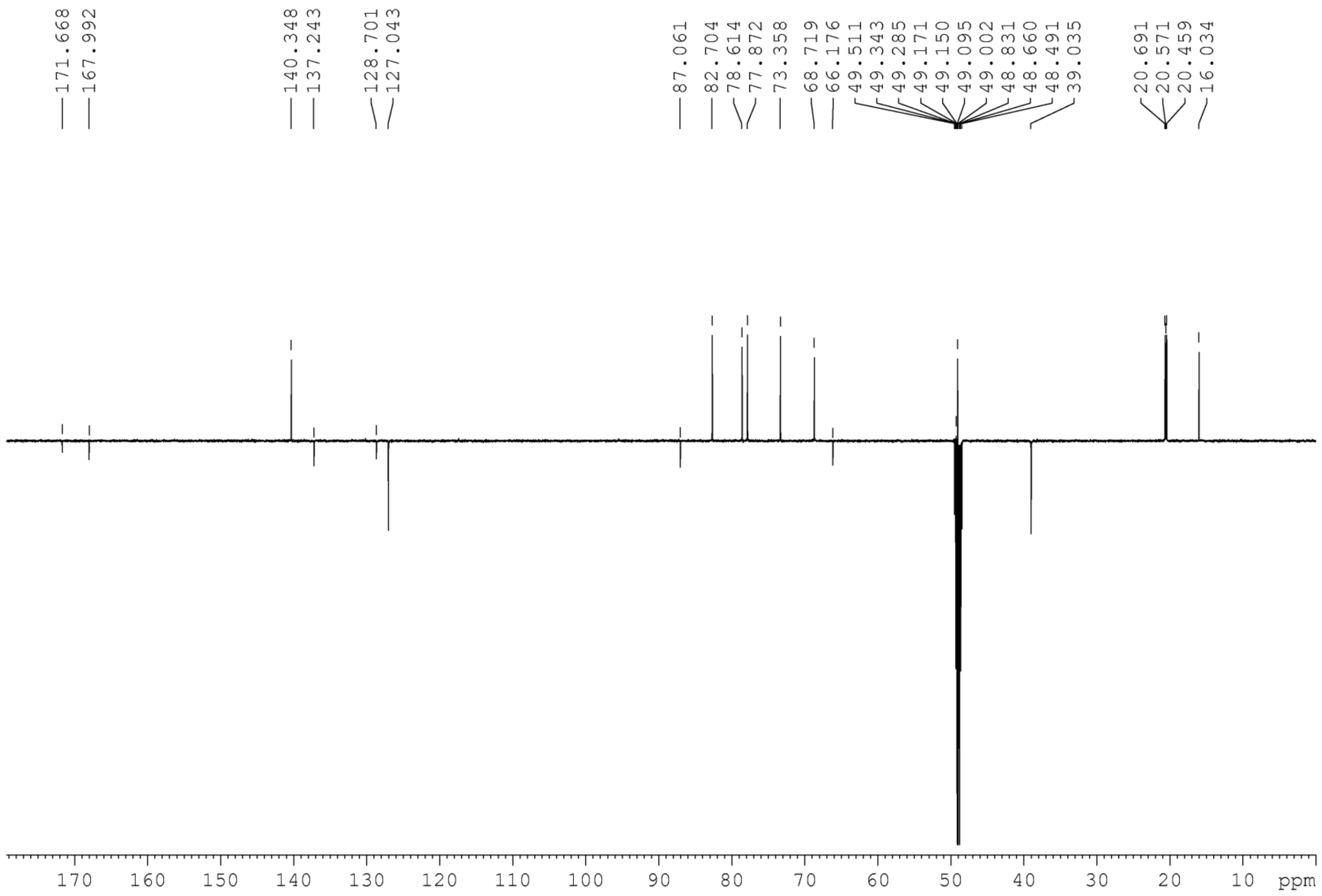

Figure S30: ^{13}C spectrum of compound **8** in CD_3OD.

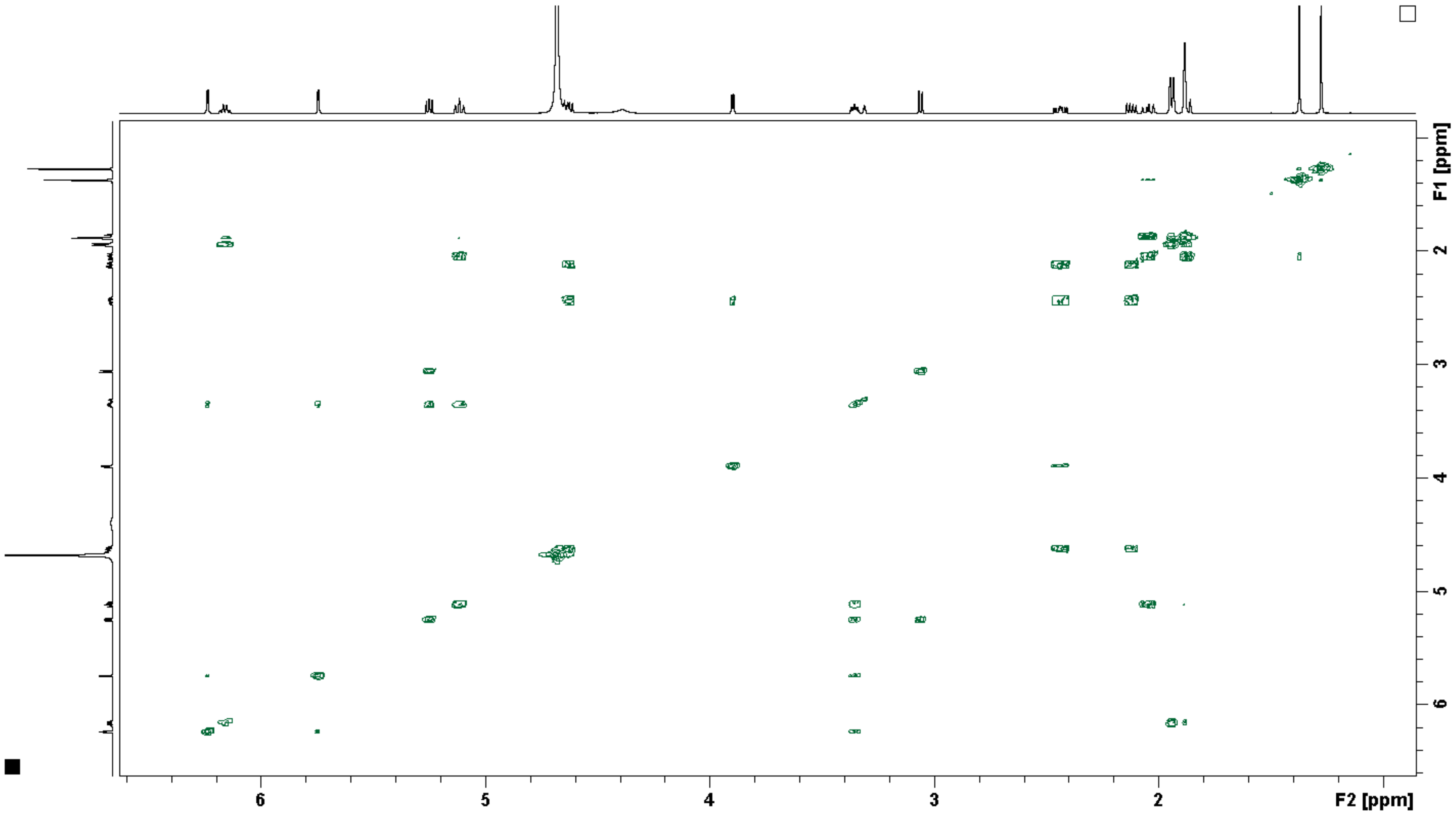

Figure S31: ^{1}H-^{1}H COSY spectrum of compound **8** in CD_3OD.

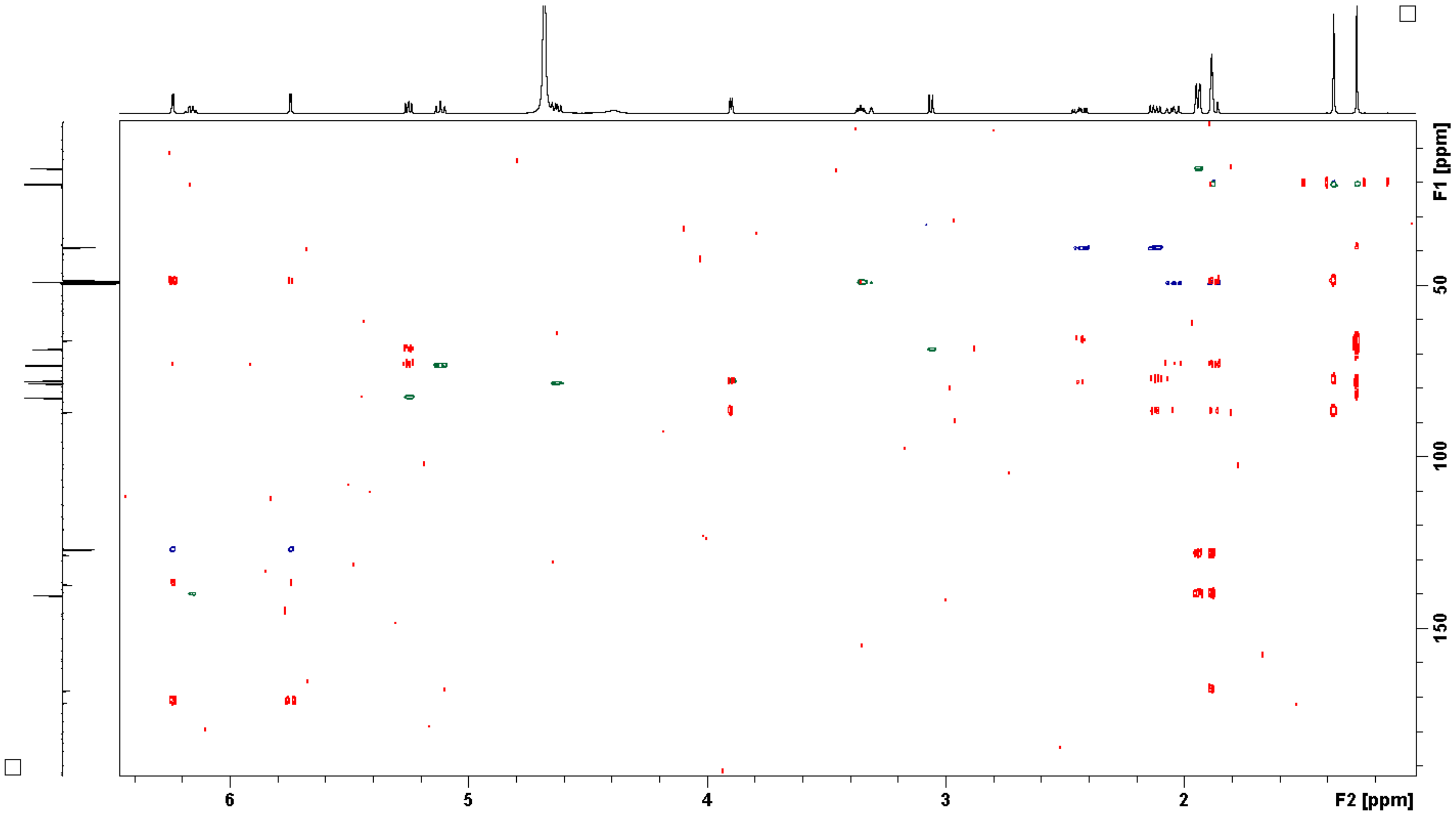

Figure S32: Overlay of HSQC and HMBC spectra of compound **8** in CD_3OD.

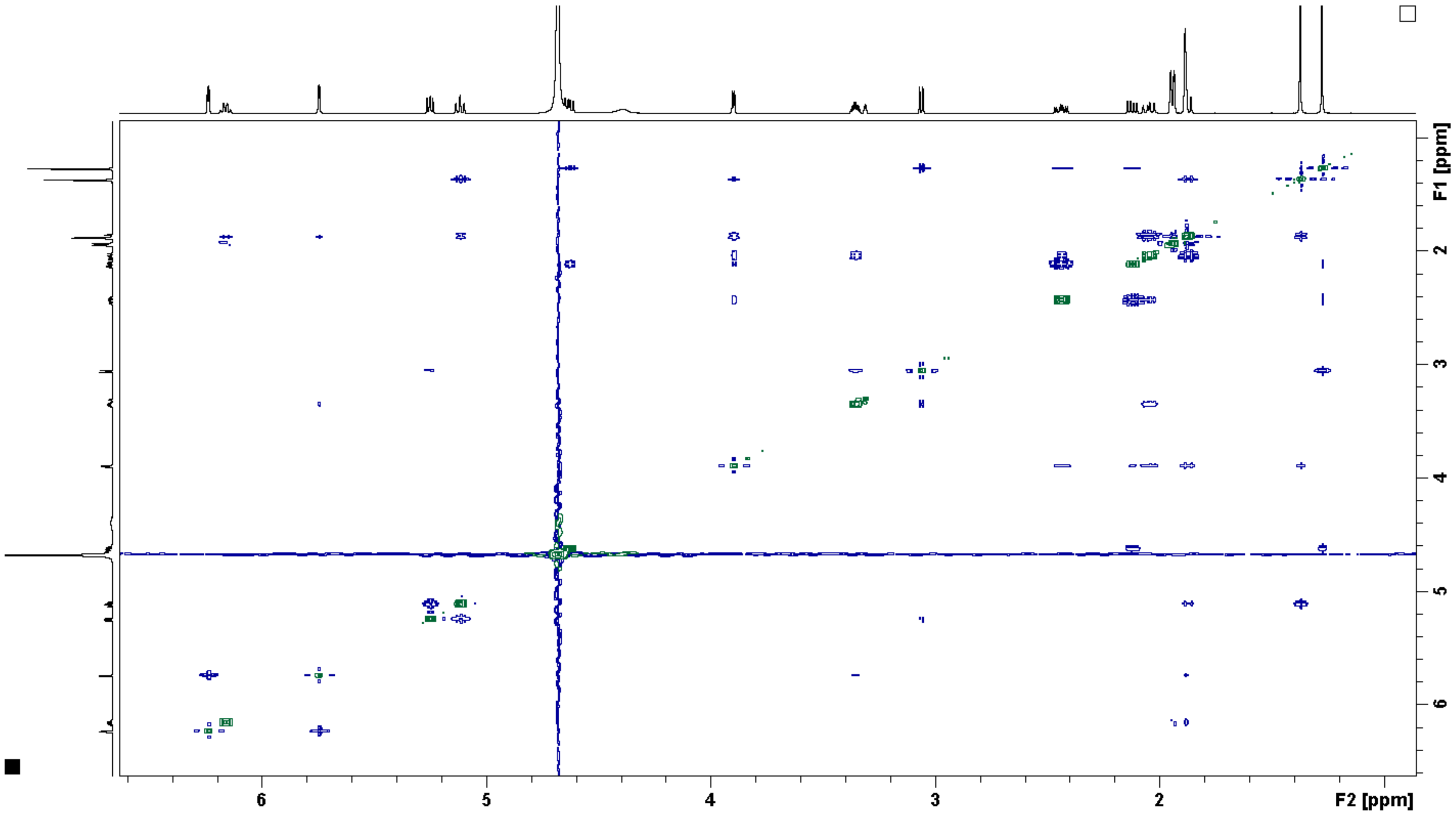

Figure S33: 2D ^{1}H-^{1}H NOESY spectrum of compound **8** in CD_3OD.

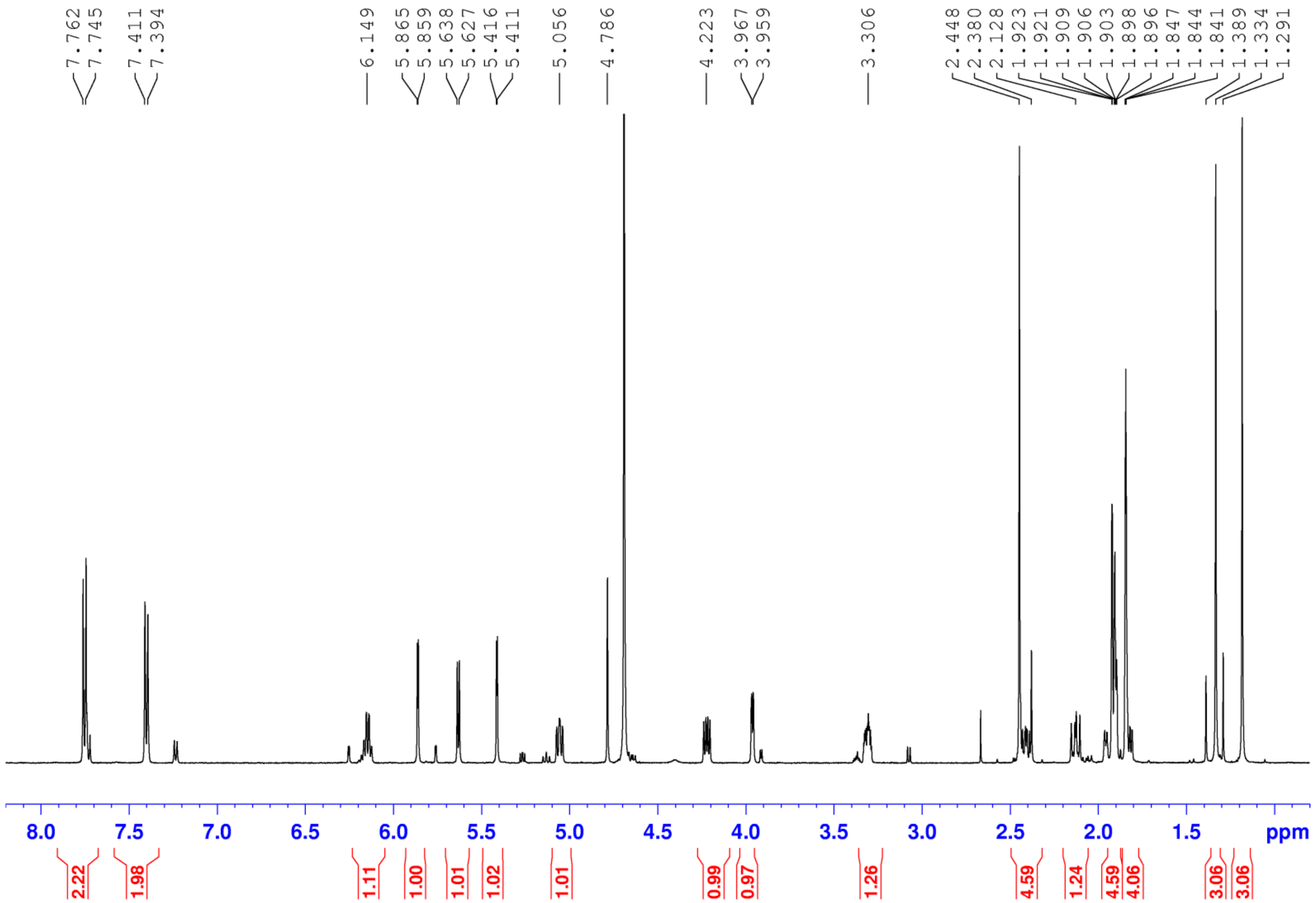

Figure S34: ^{1}H-NMR spectrum of compound **9** in CD_3OD.

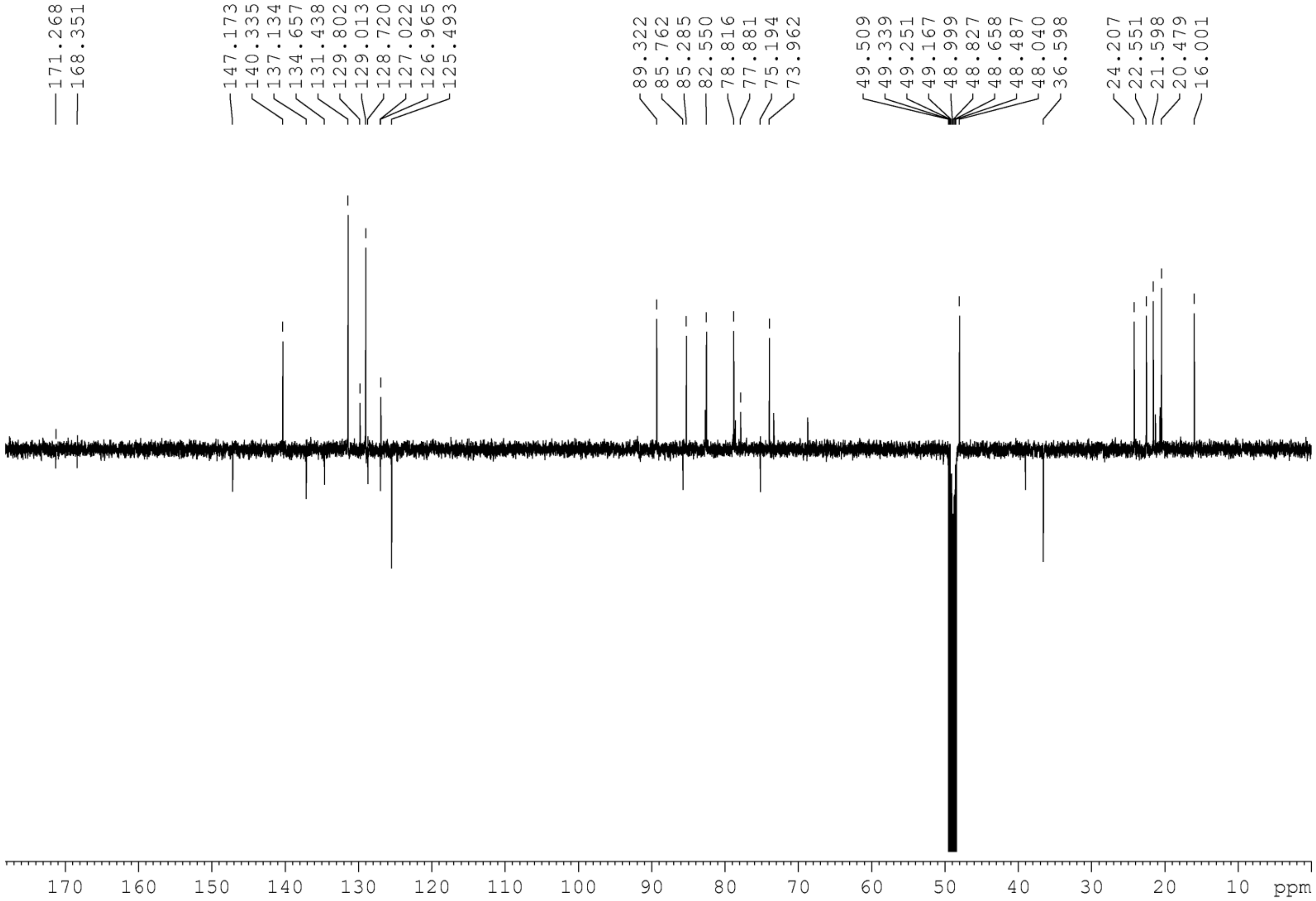

Figure S35: ^{13}C spectrum of compound **9** in CD_3OD.

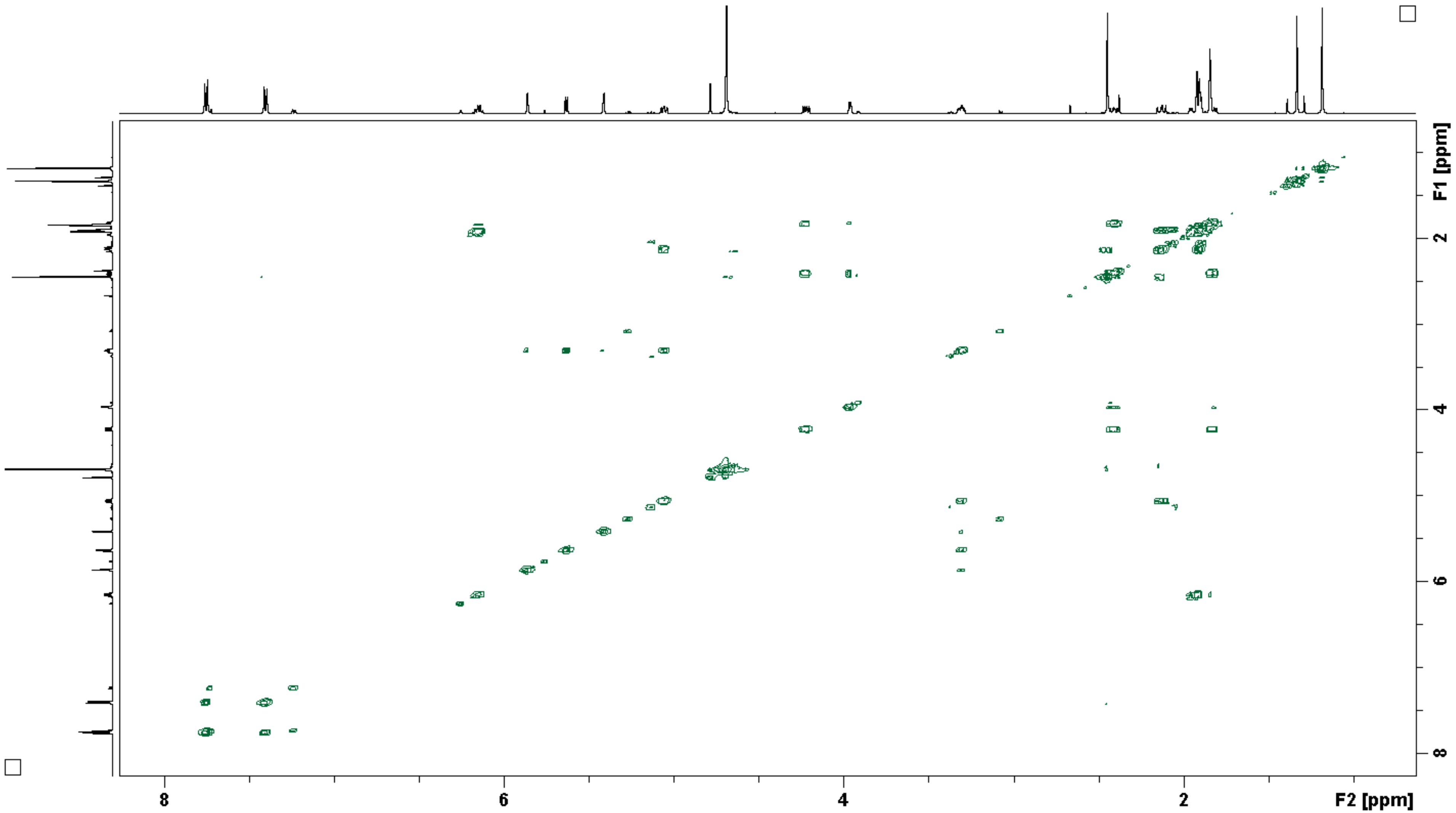

Figure S36: ^{1}H-^{1}H COSY spectrum of compound **9** in CD_3OD.

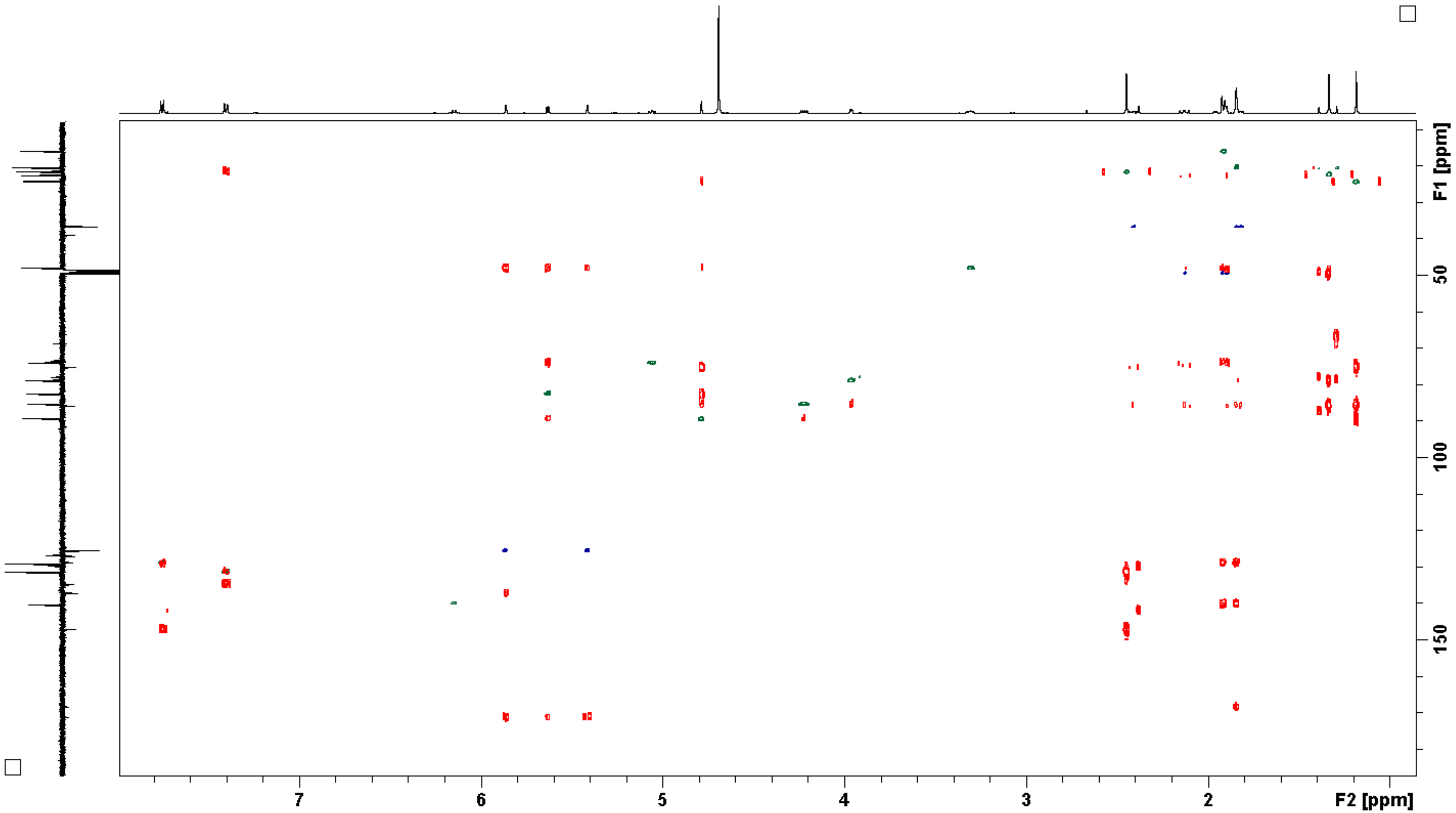

Figure S37: Overlay of HSQC and HMBC spectra of compound **9** in CD_3OD.

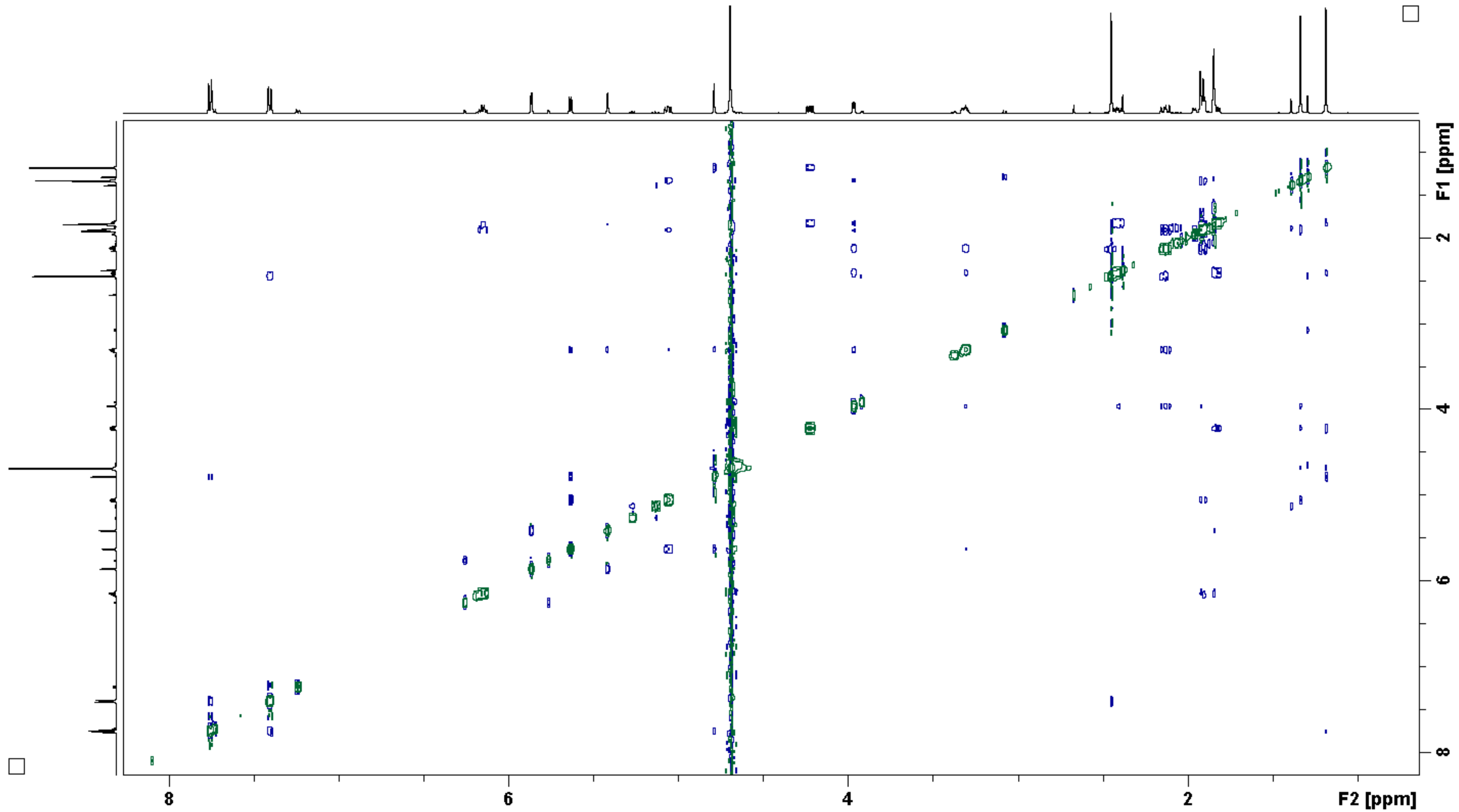

Figure S38: 2D ^1H-^1H NOESY spectrum of compound **9** in CD_3OD.

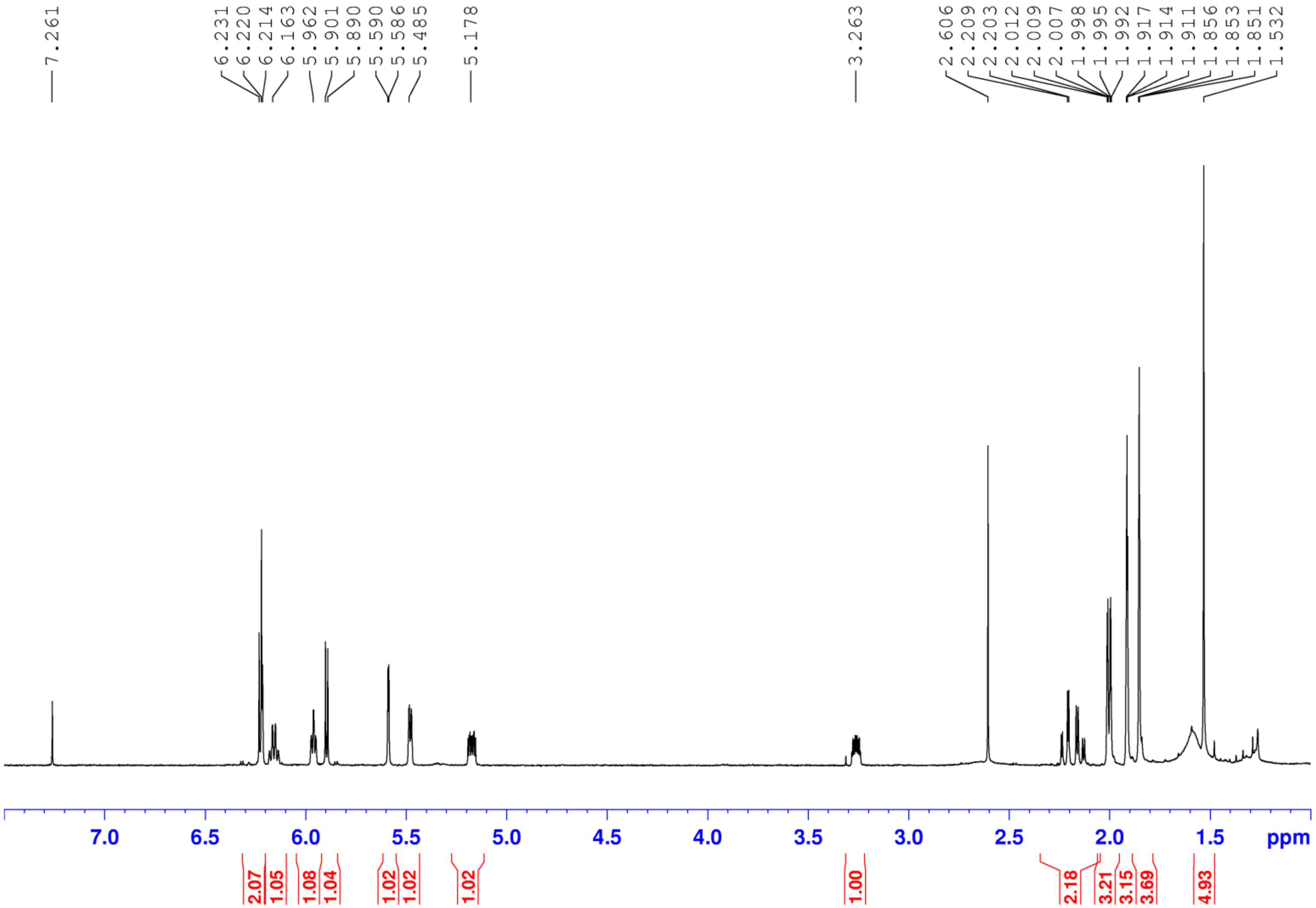

Figure S39. ^{1}H NMR spectrum of compound **10** in CD_3OD.

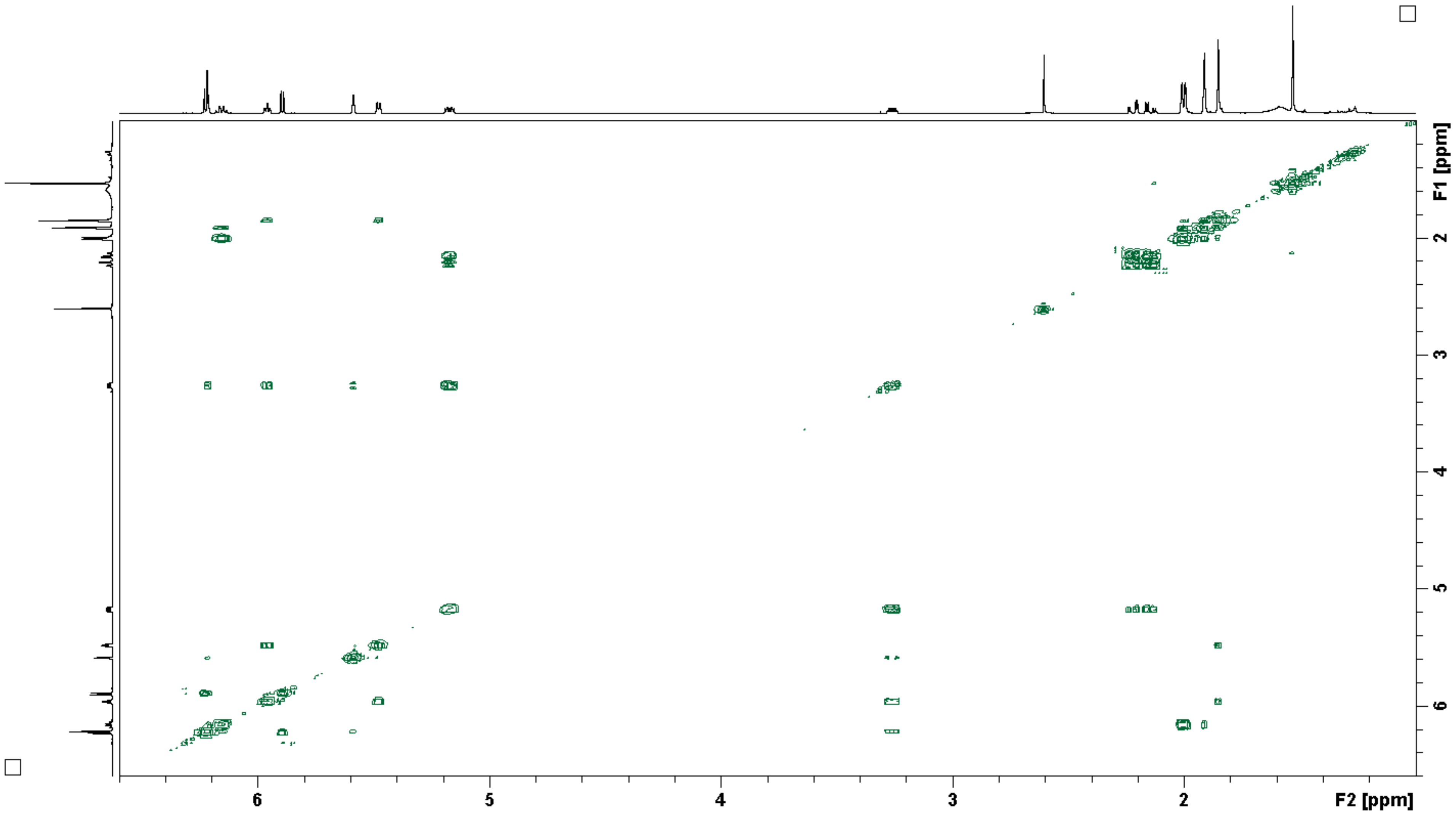

Figure S40. ^{1}H-^{1}H-COSY spectrum of compound **10** in CD_3OD.

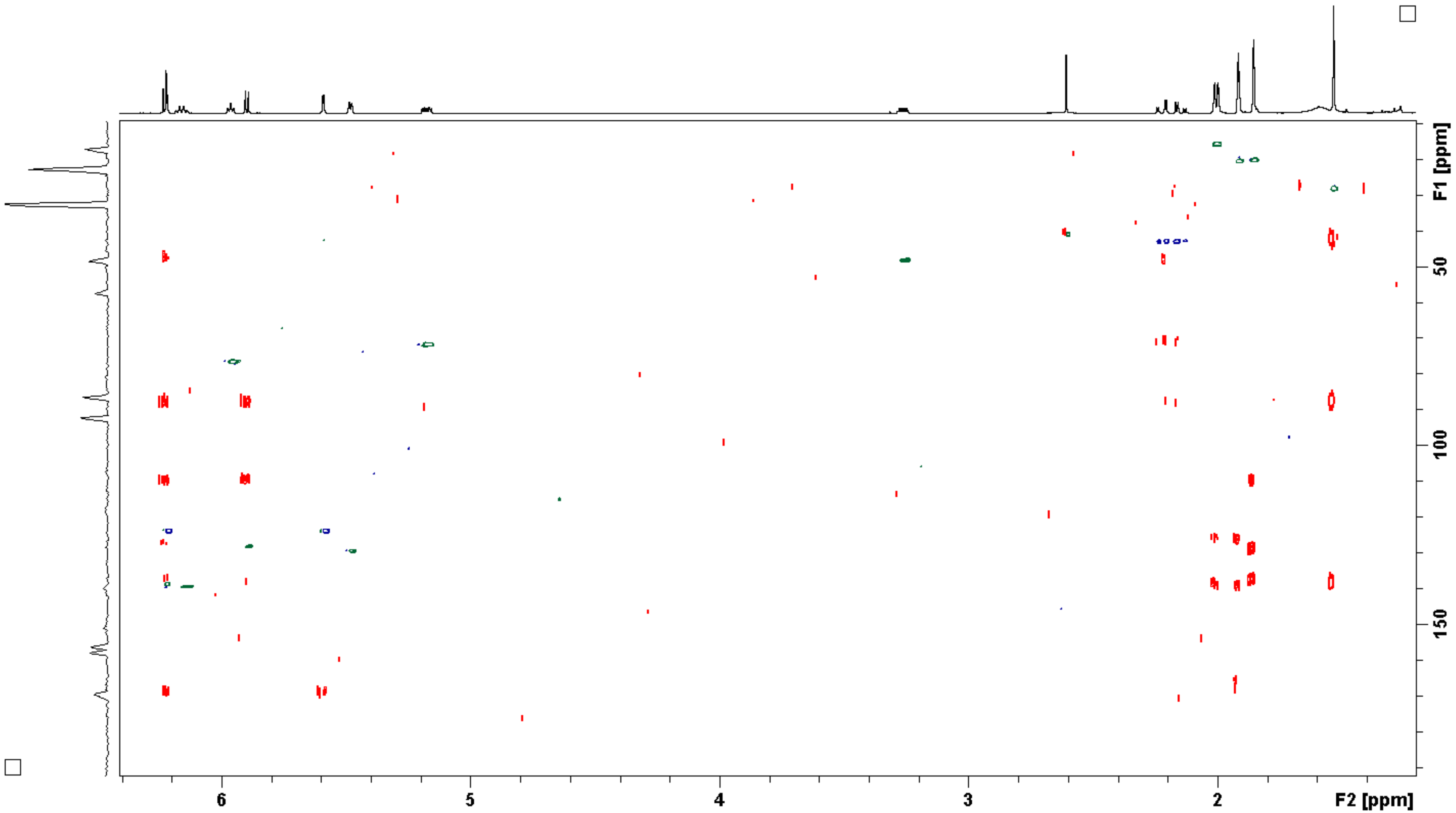

Figure S41. HSQC (green) and HMBC (red) overlaid spectra of compound **10** in CD_3OD.

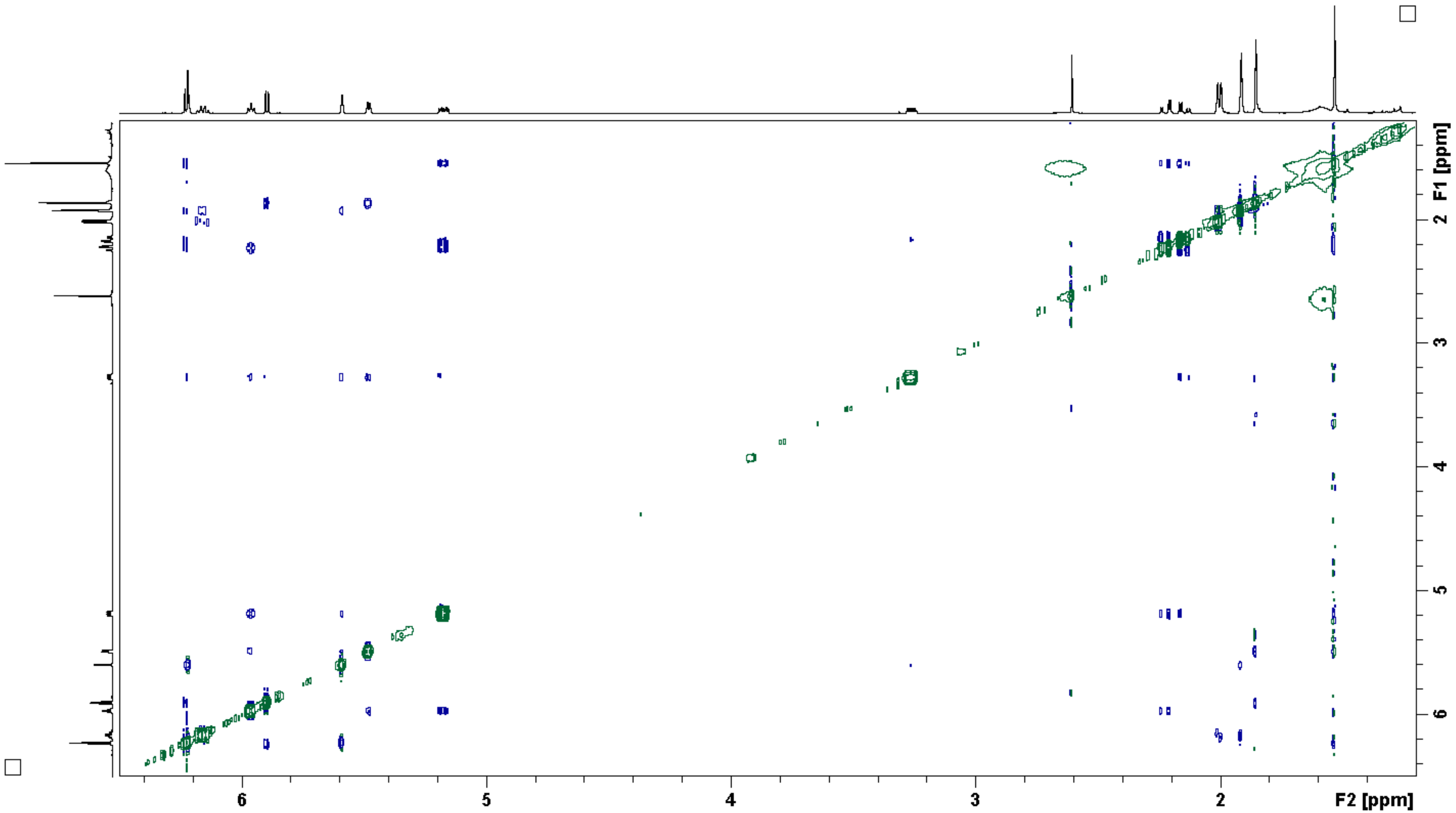

Figure S42: 2D ^{1}H-^{1}H NOESY spectrum of compound **10** in CD_3OD.

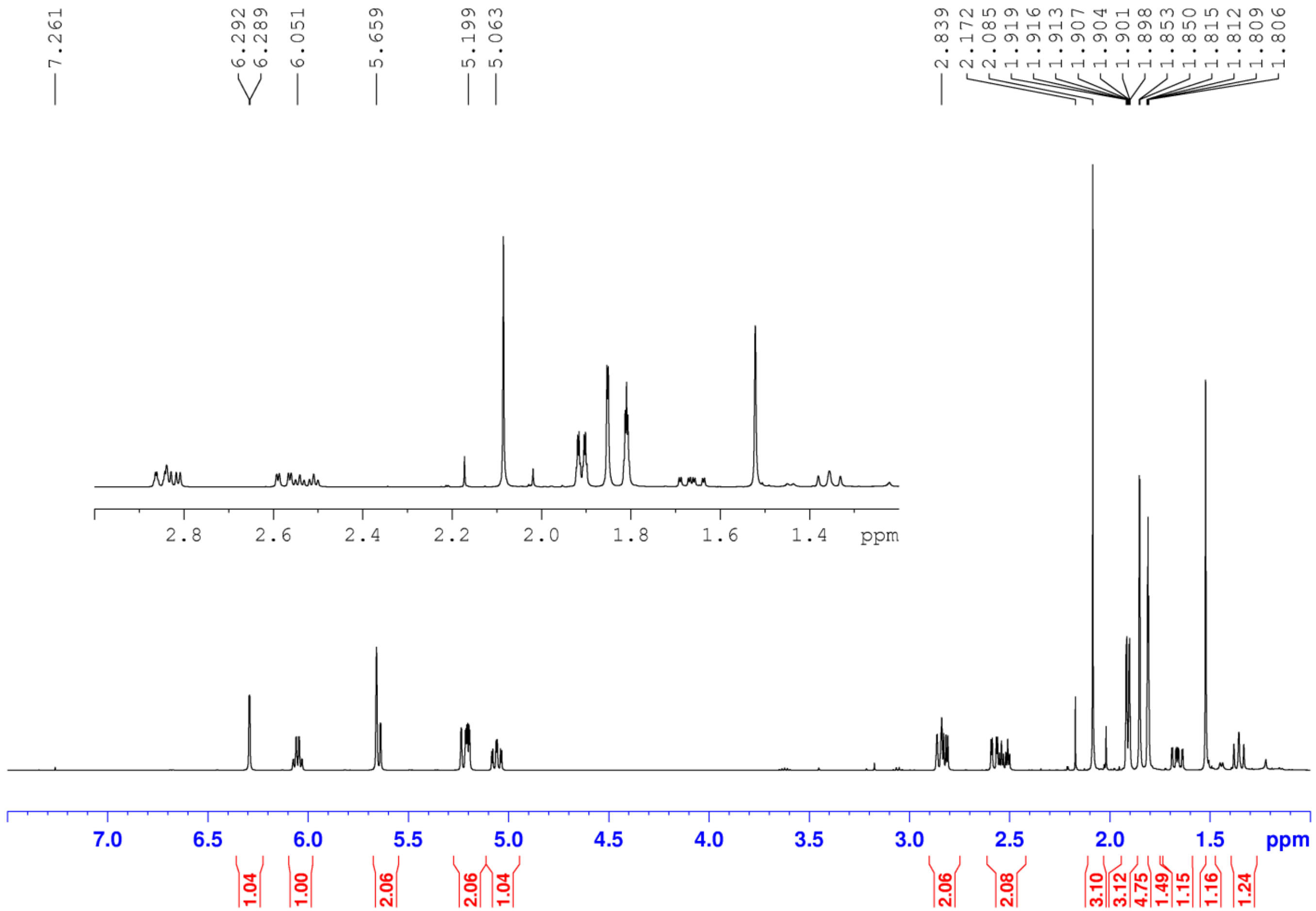

Figure S43: ^{1}H-NMR spectrum of compound **11** in $CDCl_3$.

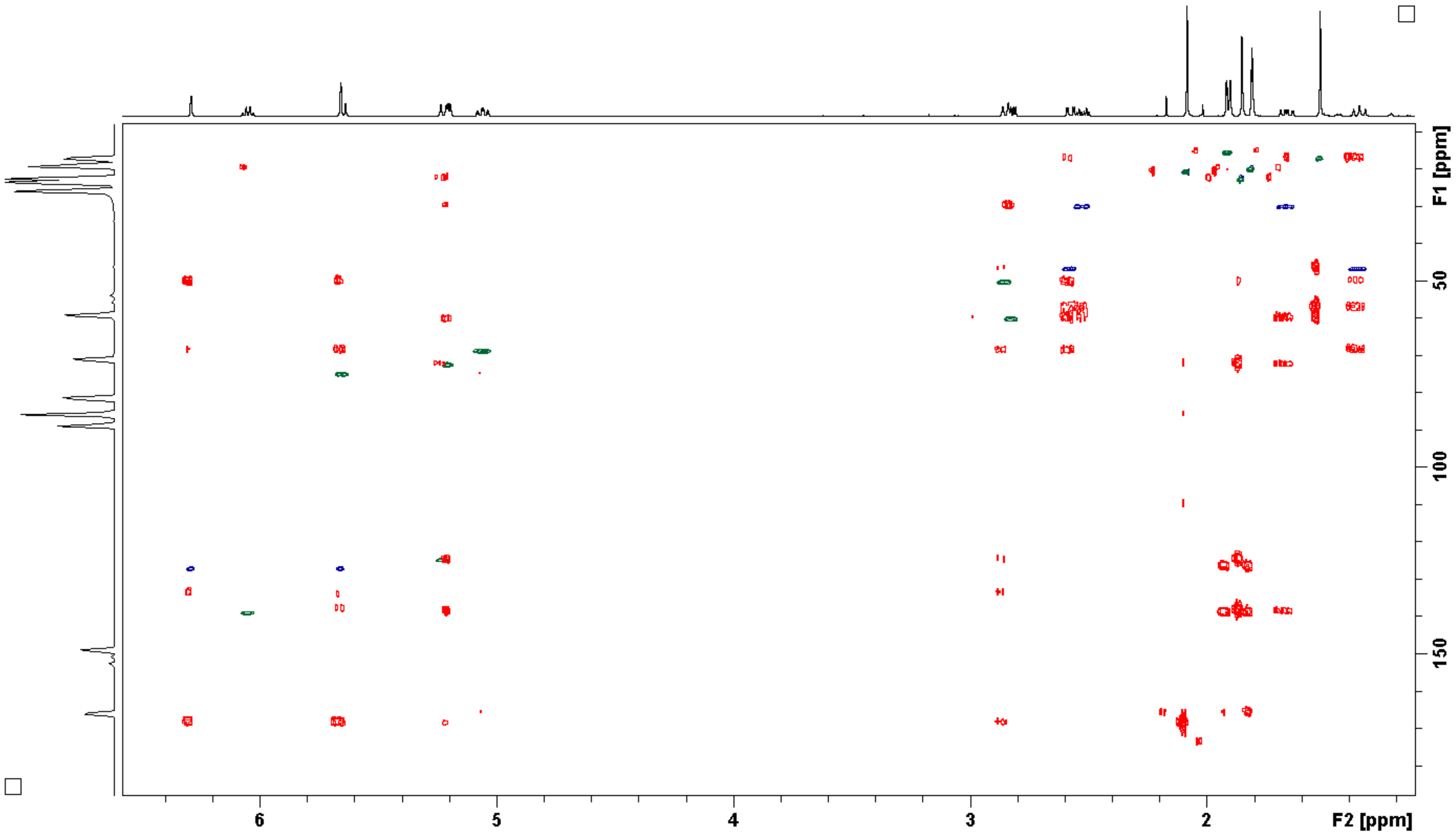

Figure S44: Overlay of HSQC and HMBC spectra of compound **11** in $CDCl_3$.

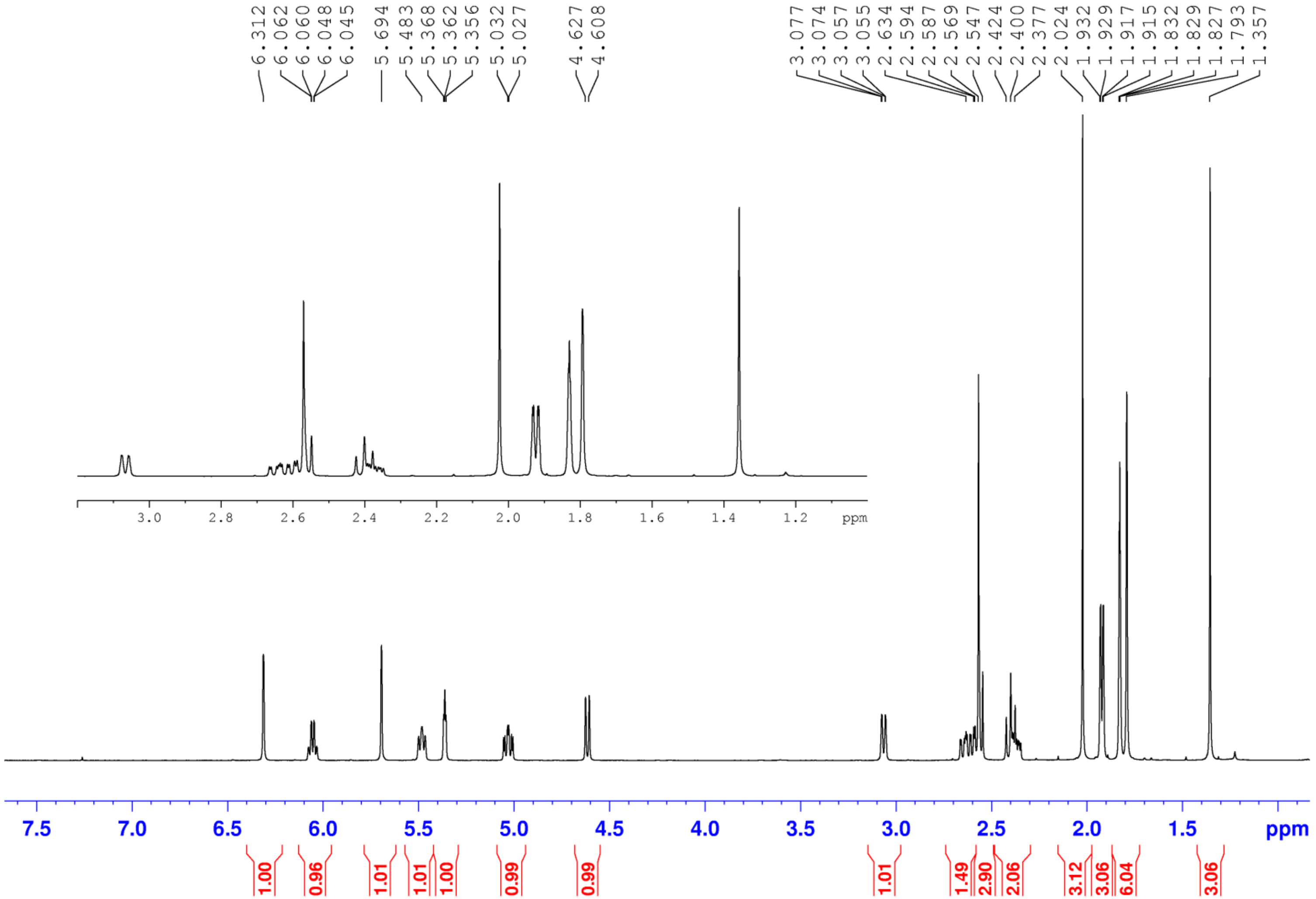

Figure S45: ^{1}H-NMR spectrum of compound **12** in $CDCl_3$.

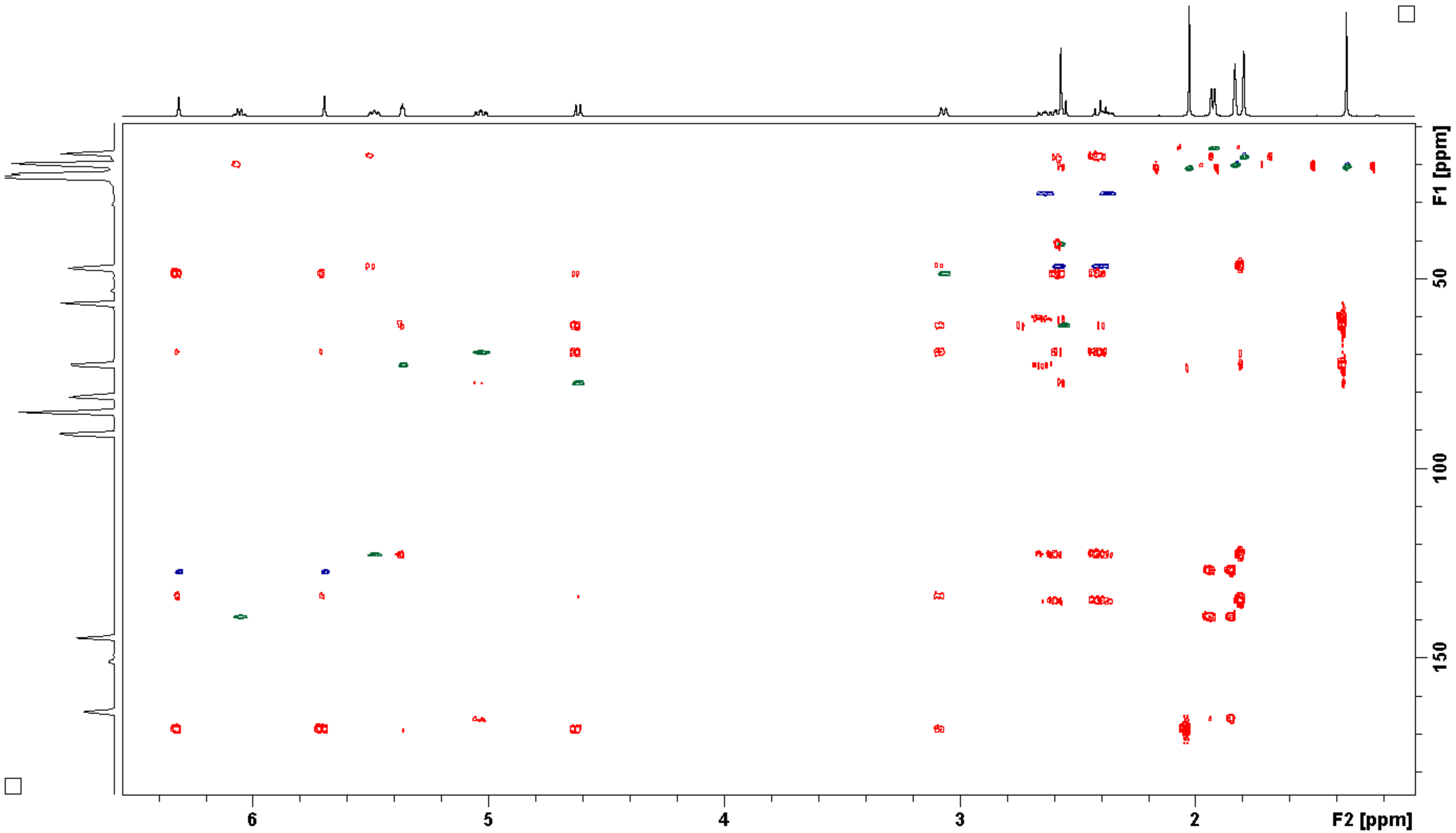

Figure S46: Overlay of HSQC and HMBC spectra of compound **12** in $CDCl_3$.

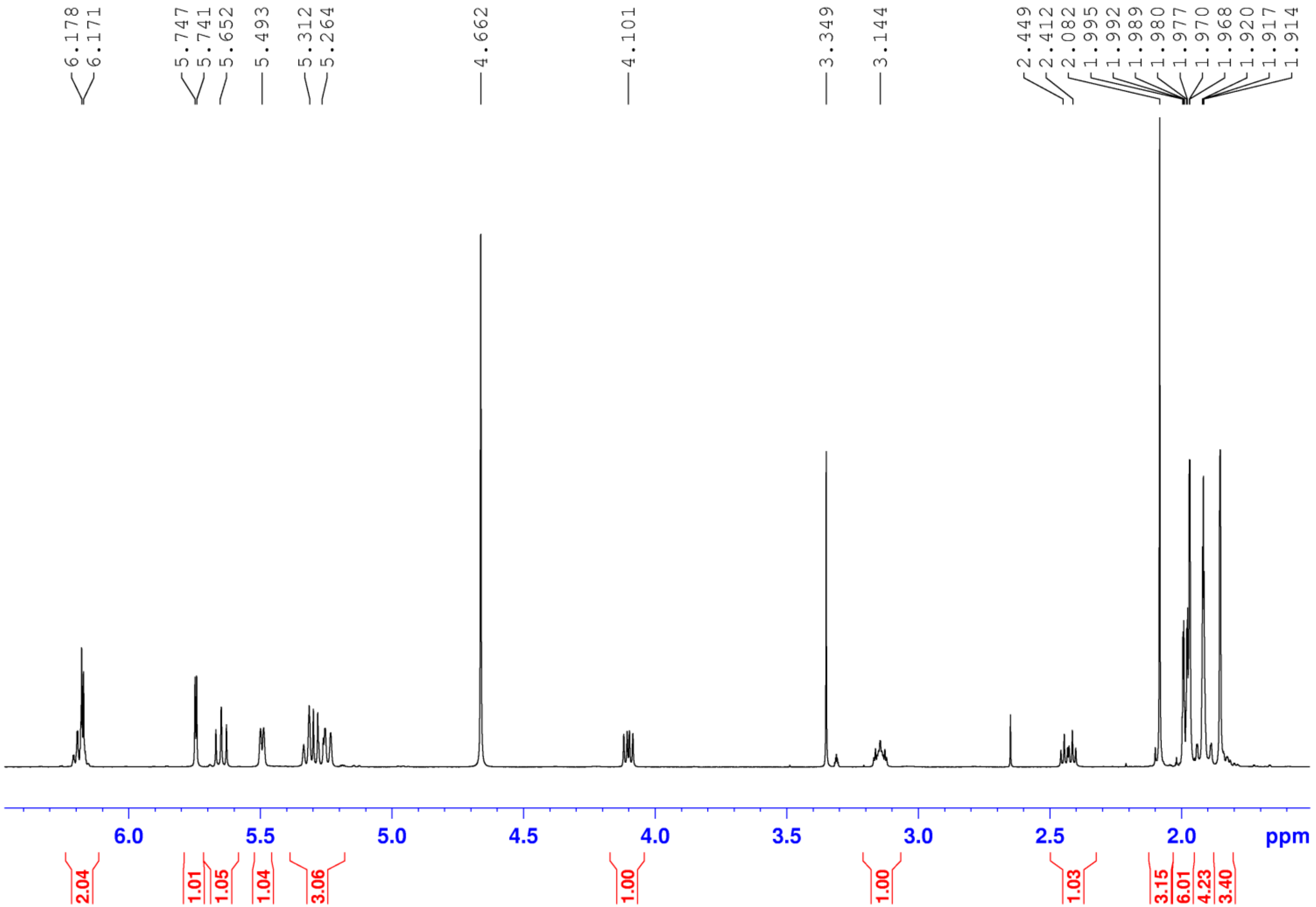

Figure S47: ^{1}H NMR spectrum of compound **13** in CD_3OD.

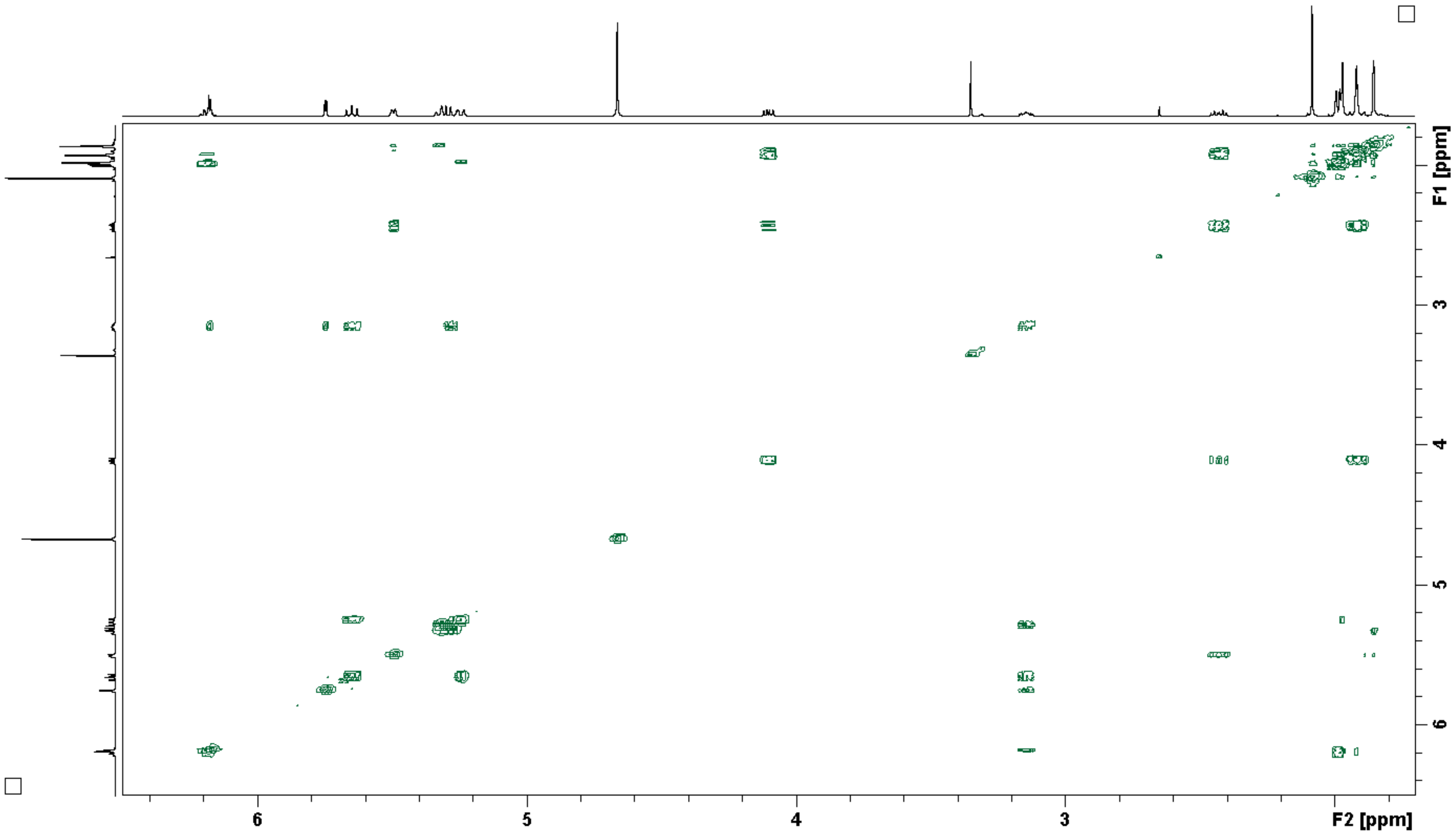

Figure S48: ^{1}H-^{1}H-COSY spectrum of compound **13** in CD_3OD.

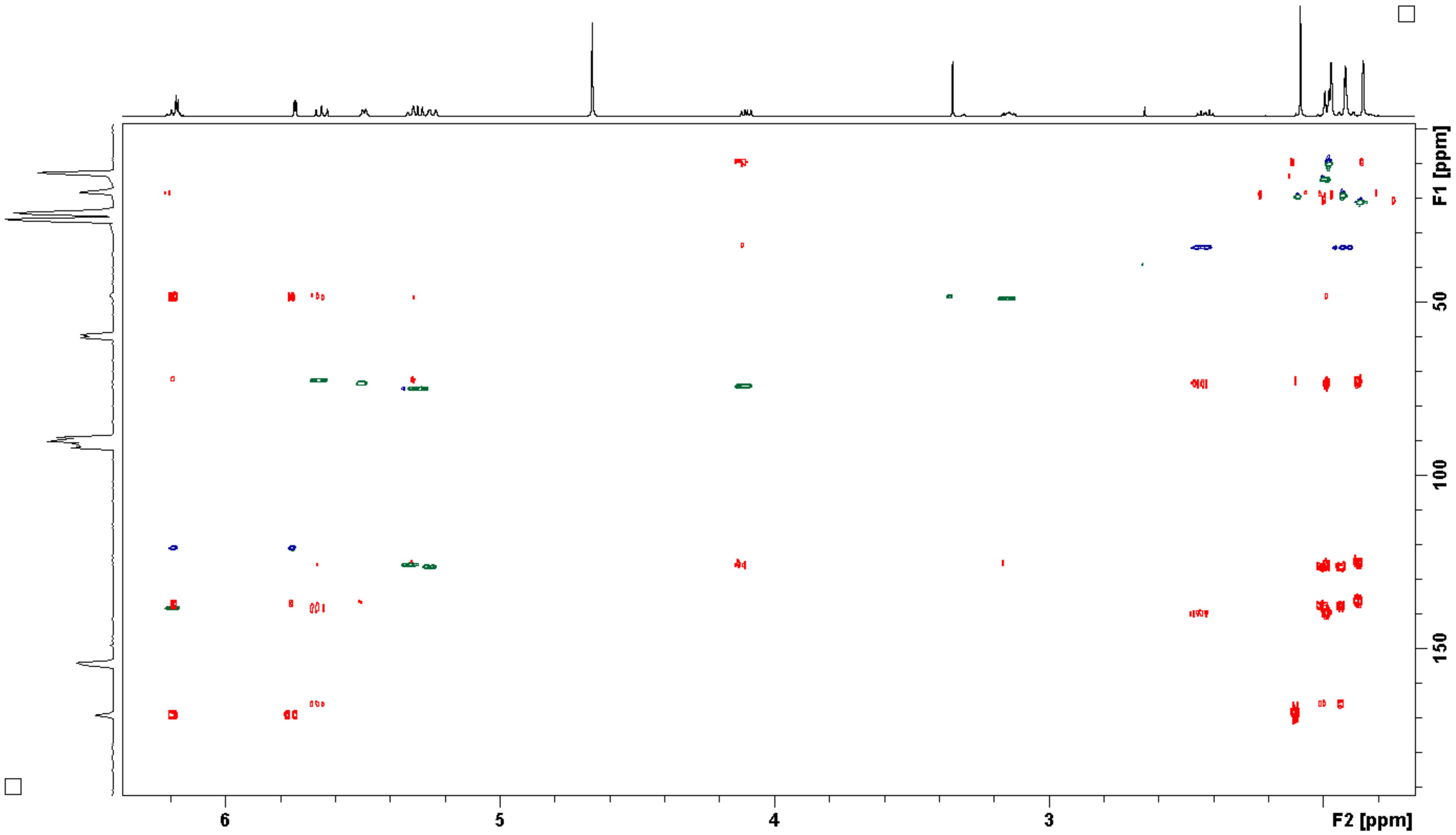

Figure S49: HSQC (green) and HMBC (red) overlaid spectra of compound **13** in CD_3OD.

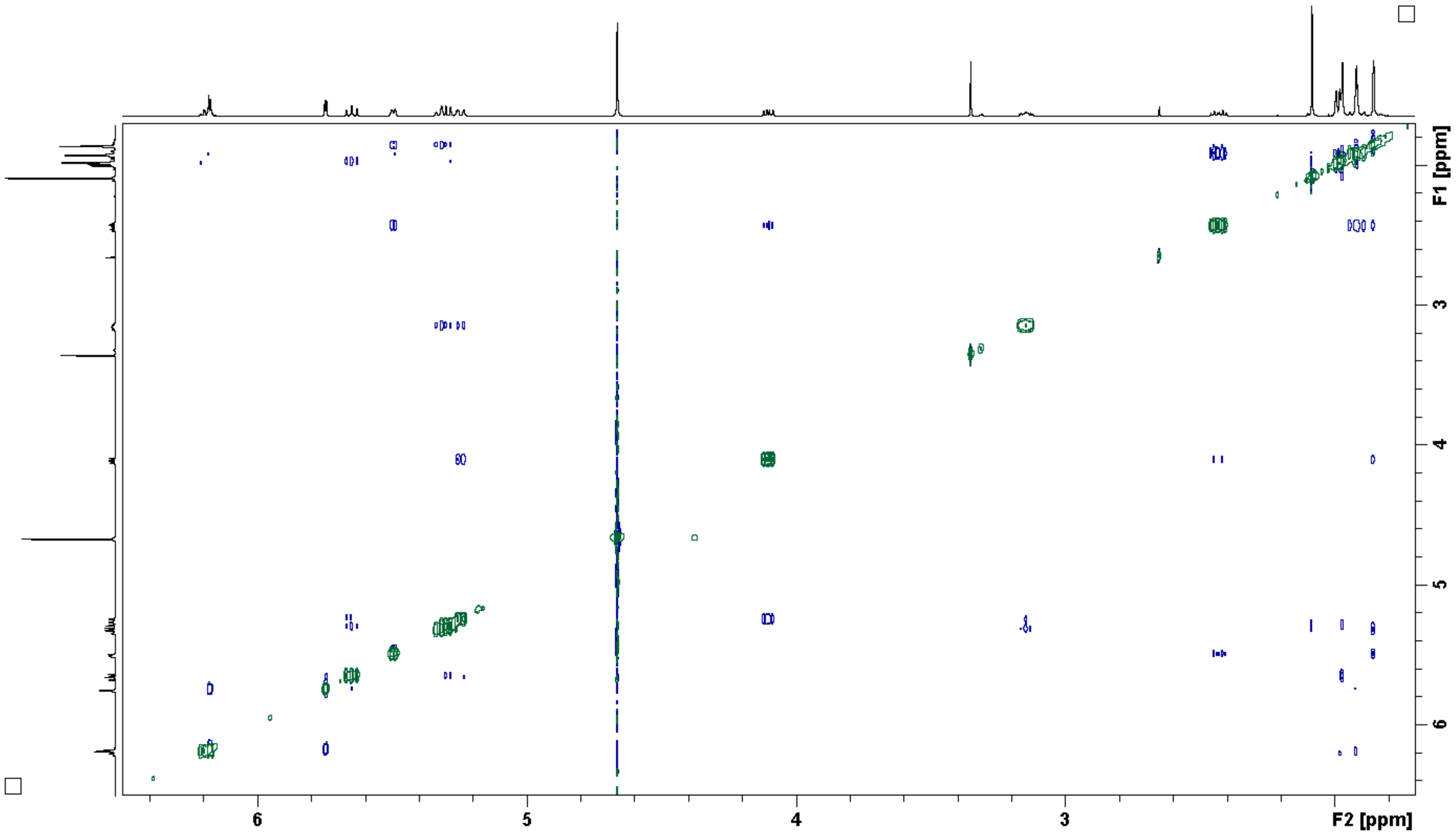

Figure S50: 2D ^1H-^1H NOESY spectrum of compound **13** in CD_3OD.

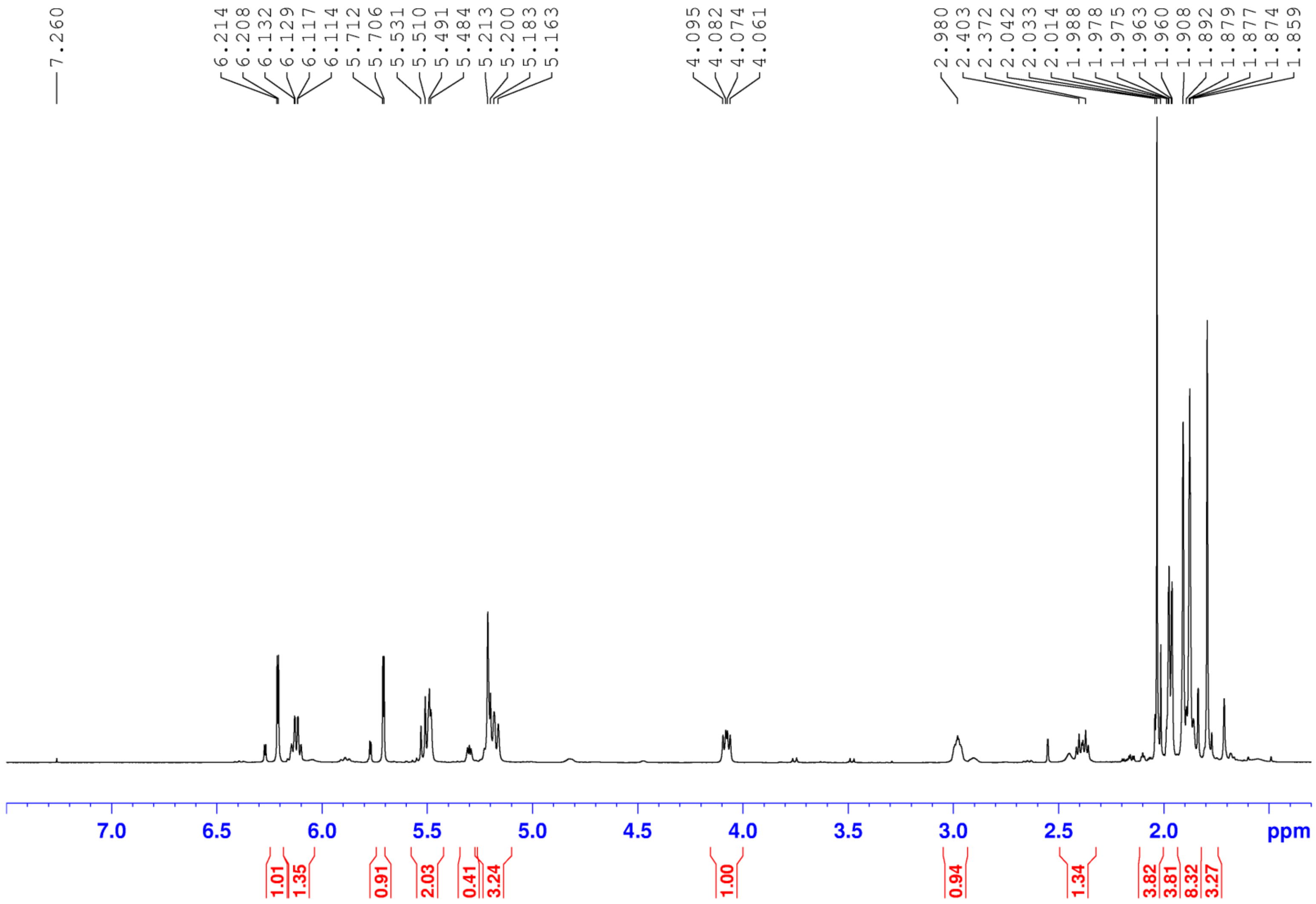

Figure S51: ^{1}H NMR spectrum of compound **14** in CD_3OD.

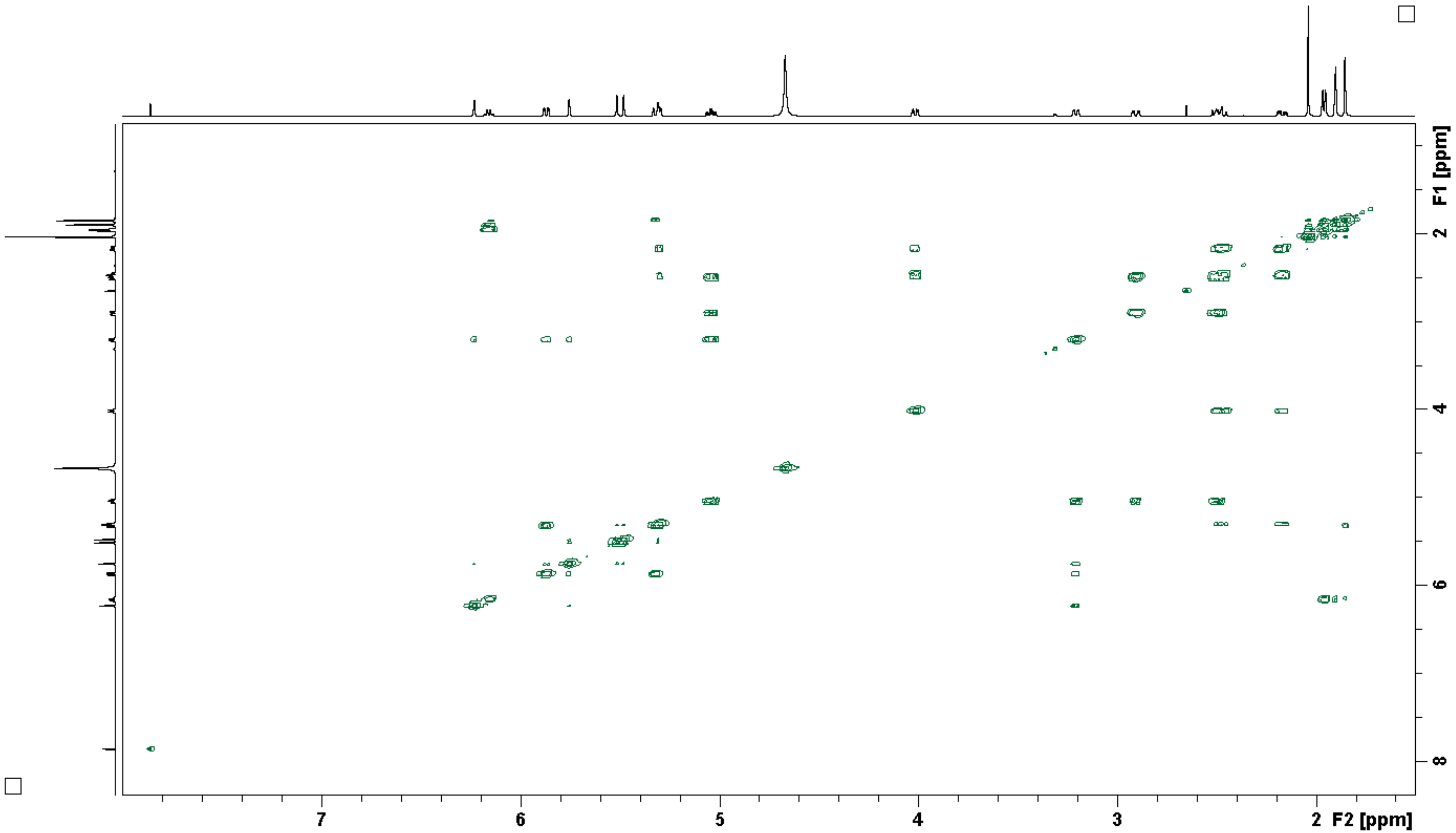

Figure S52: ^{1}H-^{1}H-COSY spectrum of compound **14** in CD_3OD.

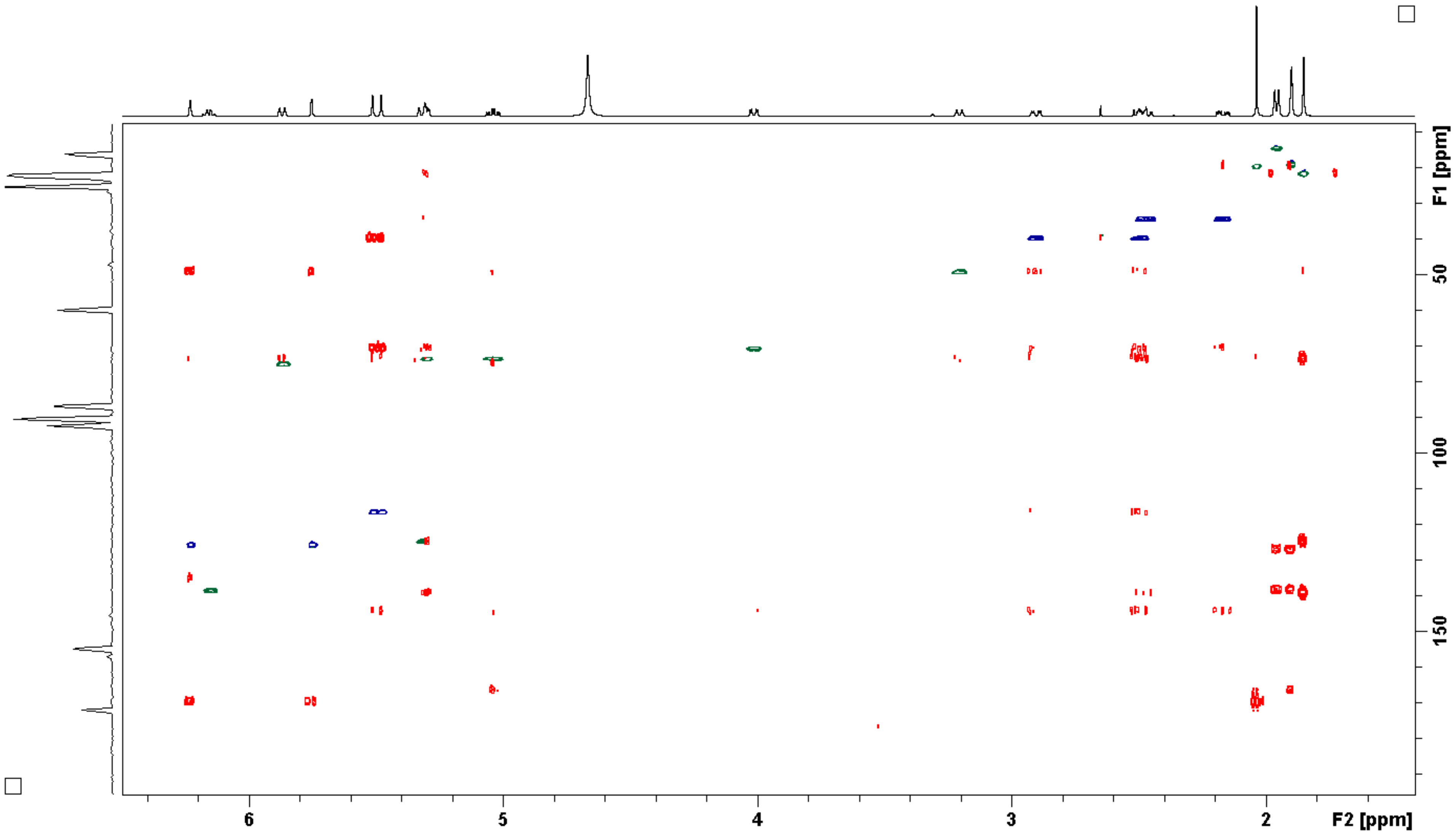

Figure S53: HSQC (green) and HMBC (red) overlaid spectra of compound **14** in CD_3OD.

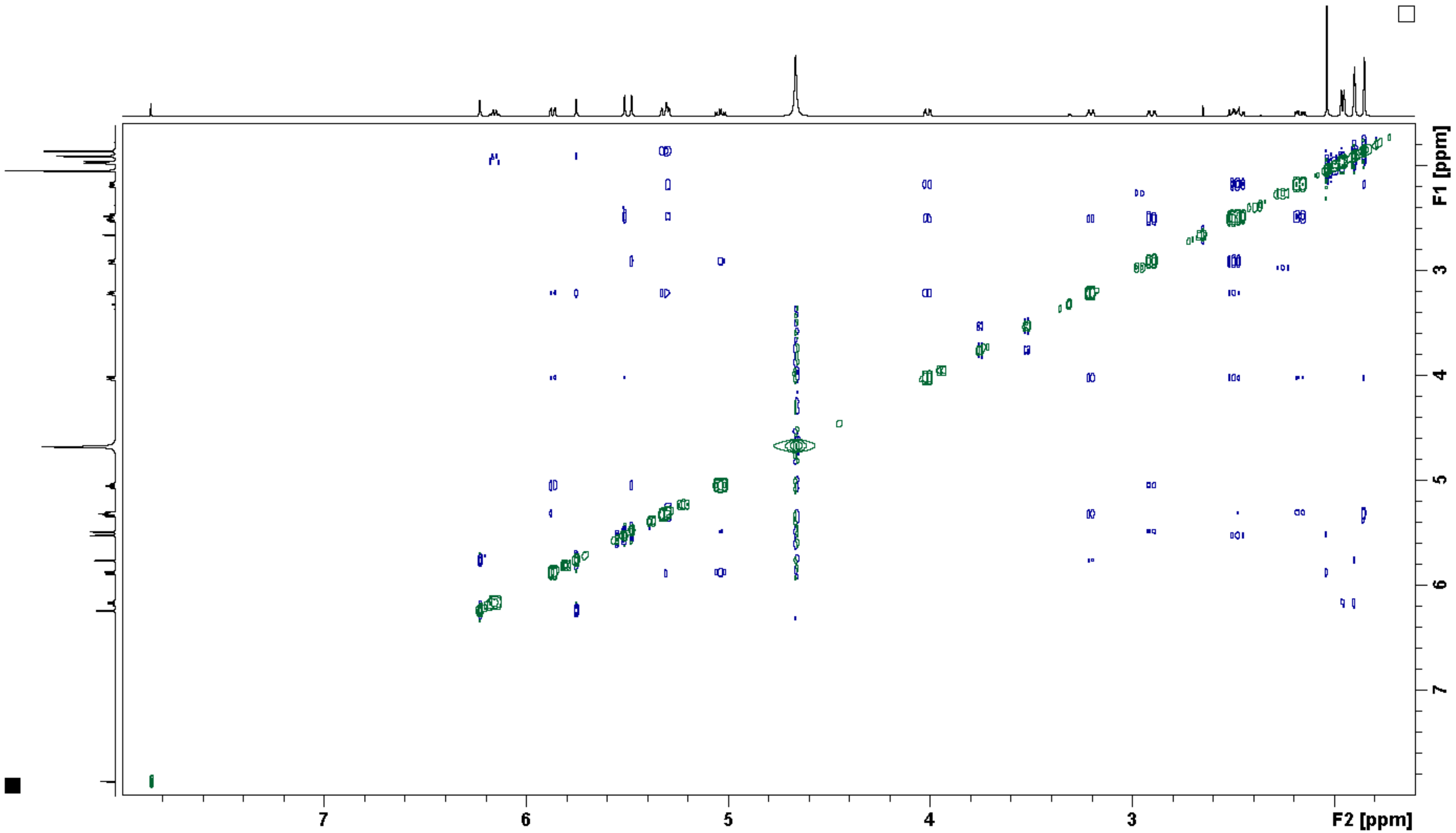

Figure S54: 2D ^{1}H-^{1}H NOESY spectrum of compound **14** in CD_3OD.

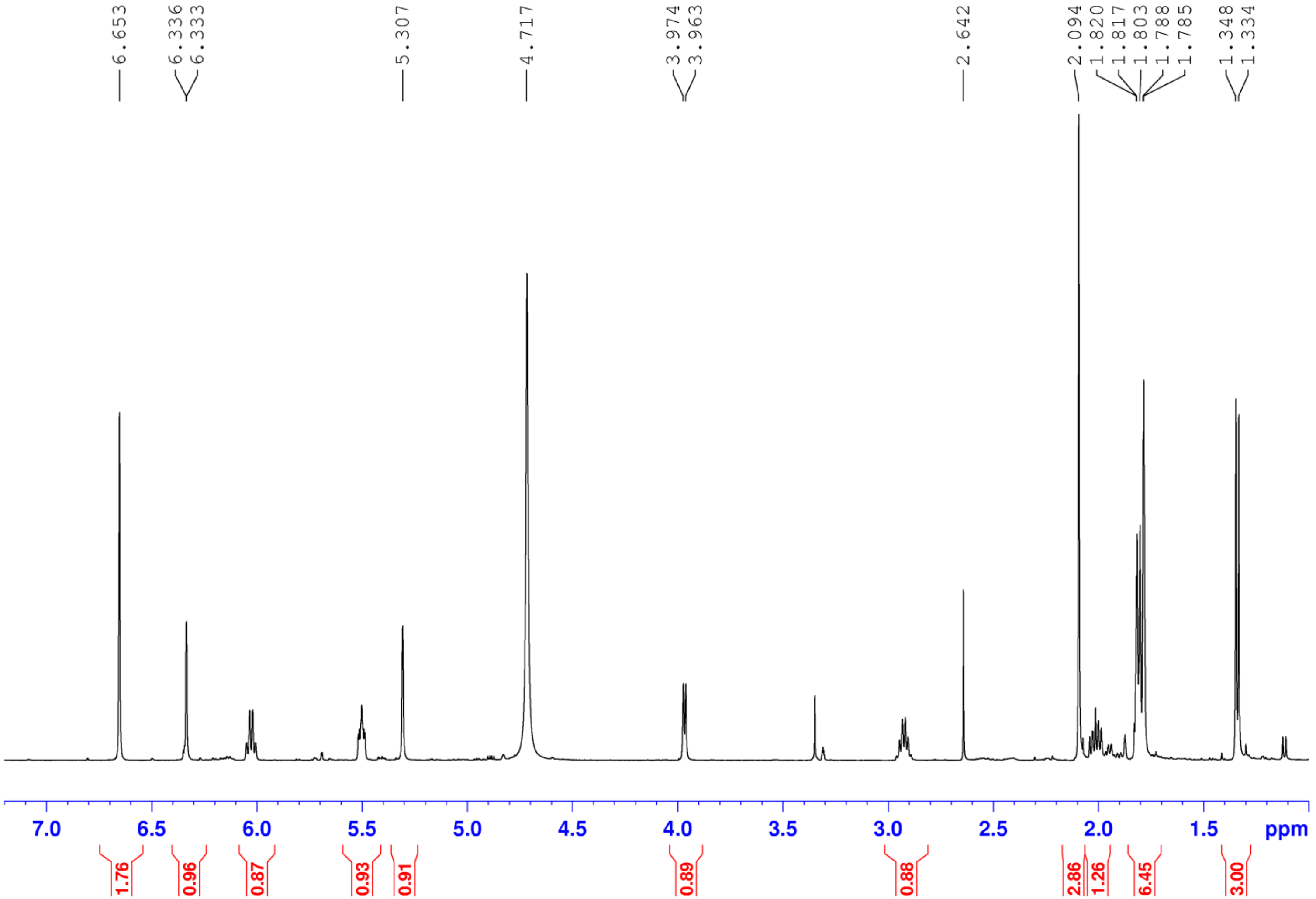

Figure S55: ^{1}H NMR spectrum of compound **15** in CD_3OD.

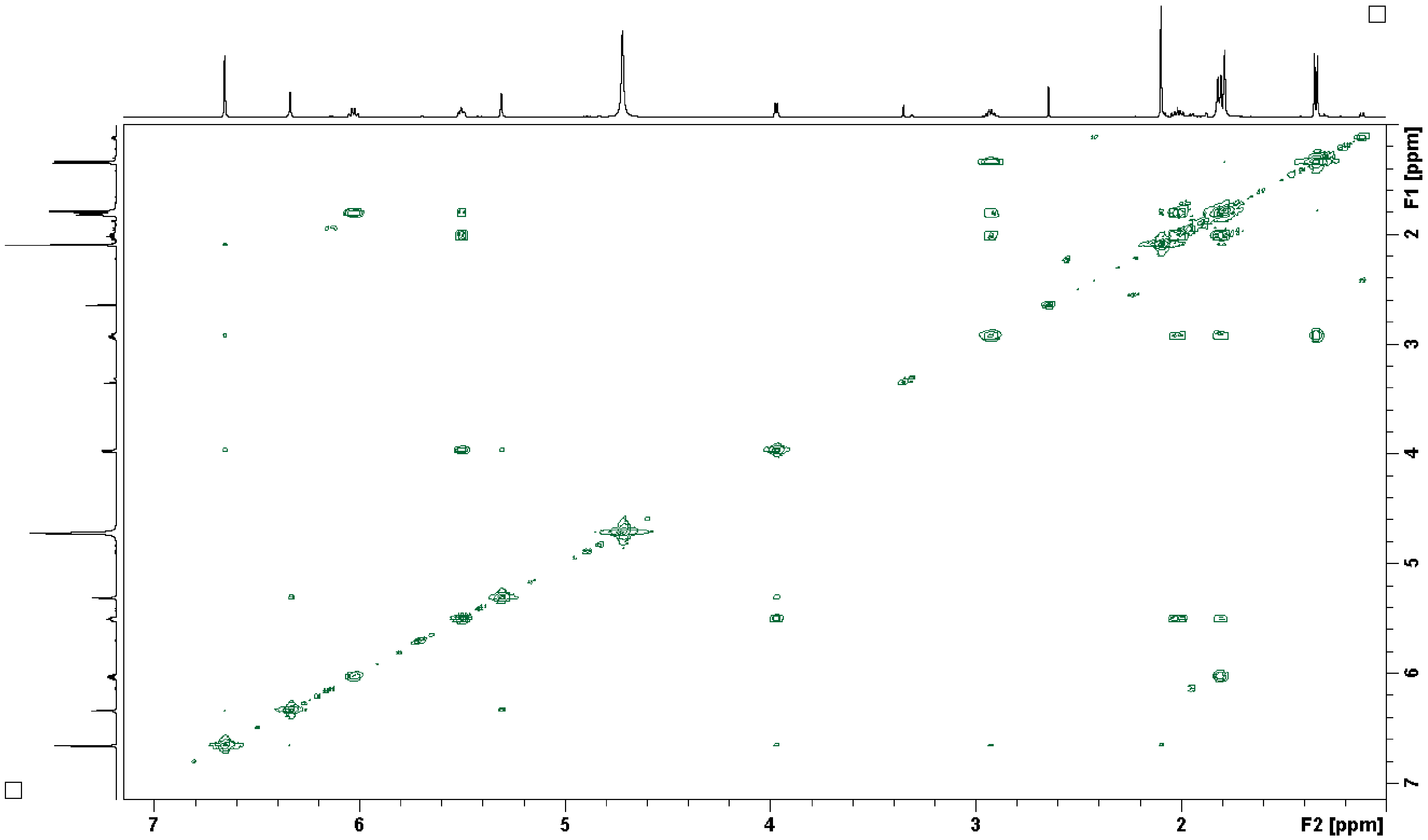

Figure S56: ^{1}H-^{1}H-COSY spectrum of compound **15** in CD_3OD.

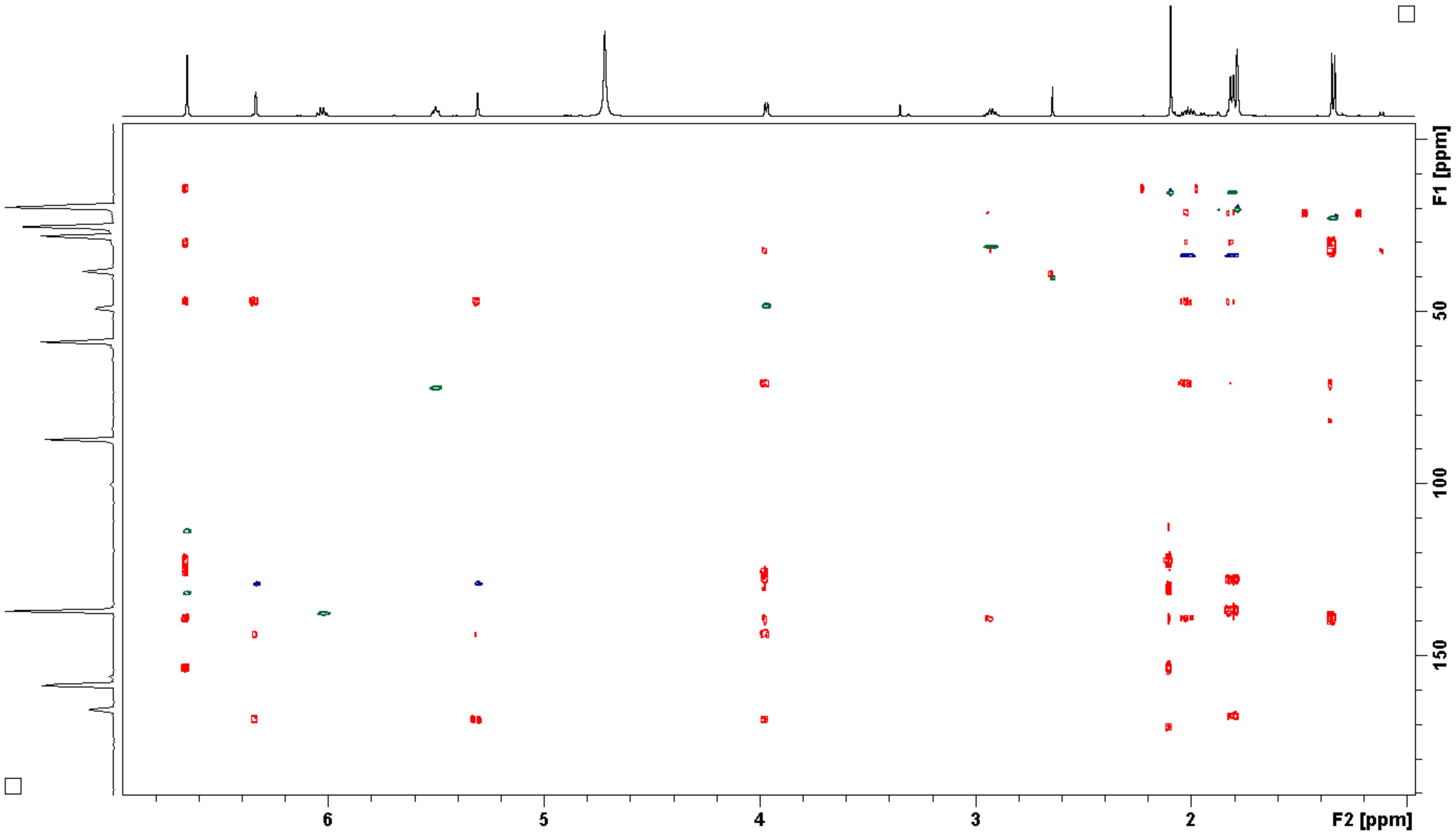

Figure S57: HSQC (green) and HMBC (red) overlaid spectra of compound **15** in CD_3OD.

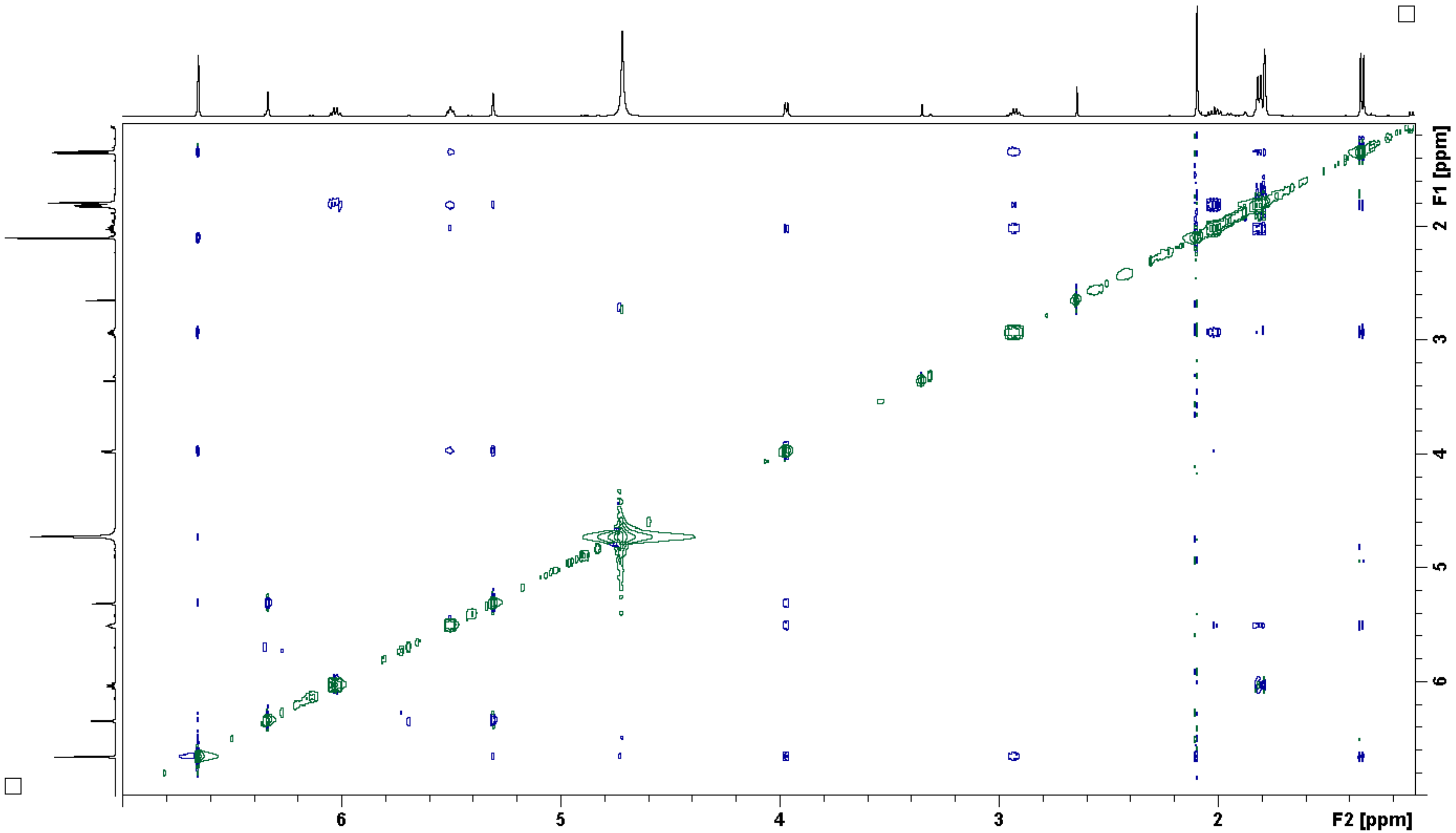

Figure S58: 2D ^1H-^1H NOESY spectrum of compound **15** in CD_3OD.

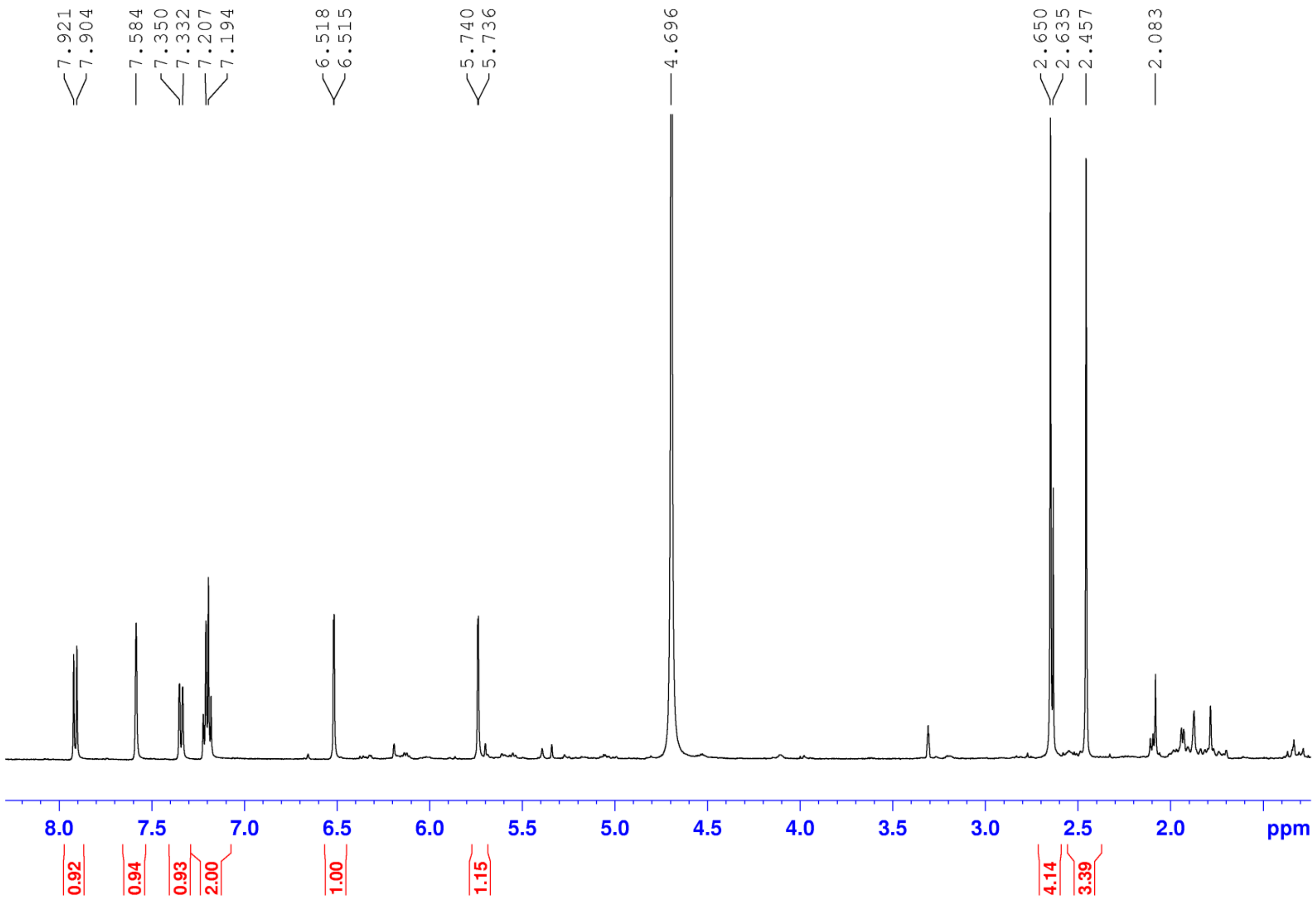

Figure S59: ^{1}H NMR spectrum of compound **16** in CD_3OD.

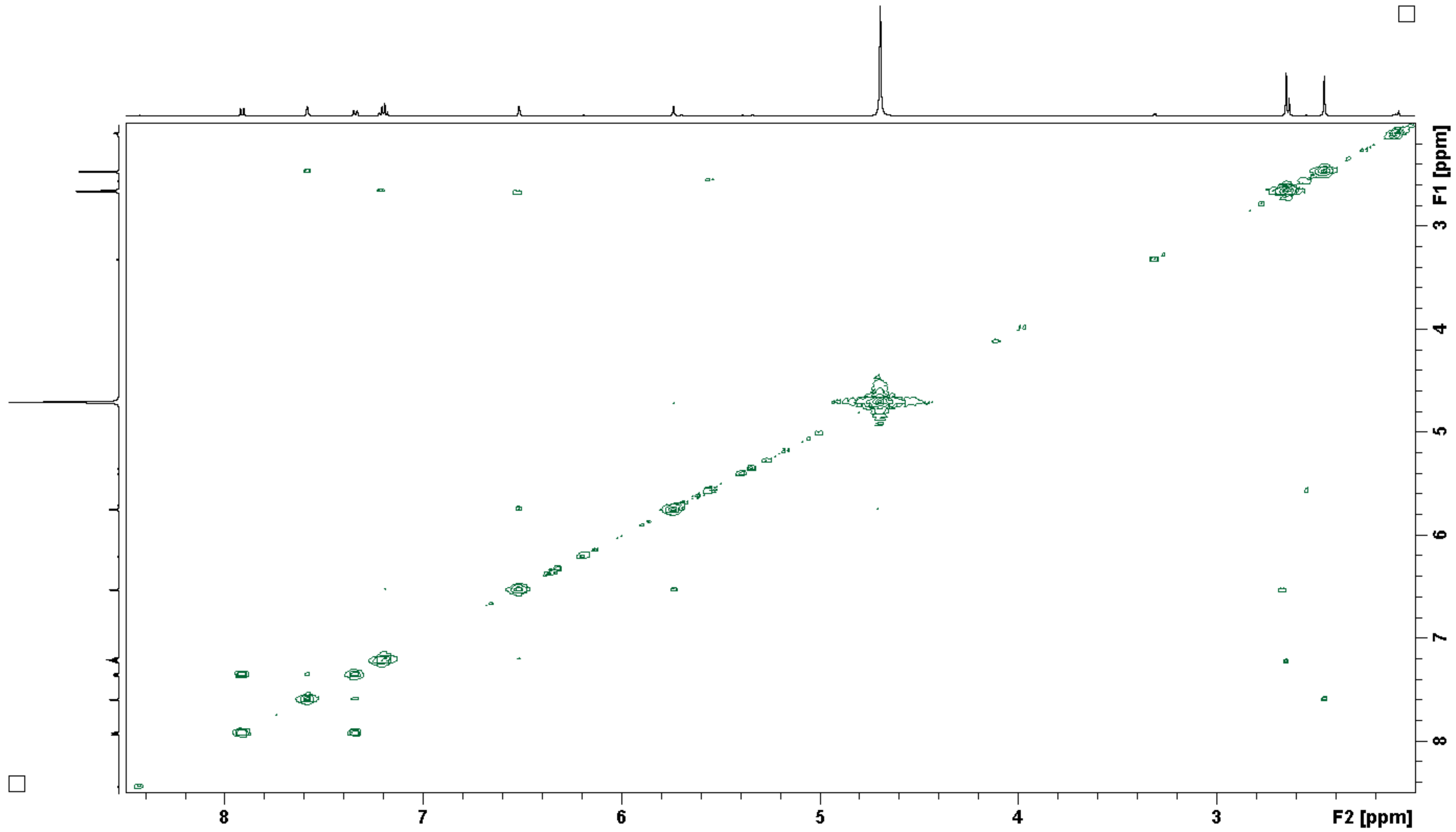

Figure S60: ^{1}H-^{1}H-COSY spectrum of compound **16** in CD_3OD.

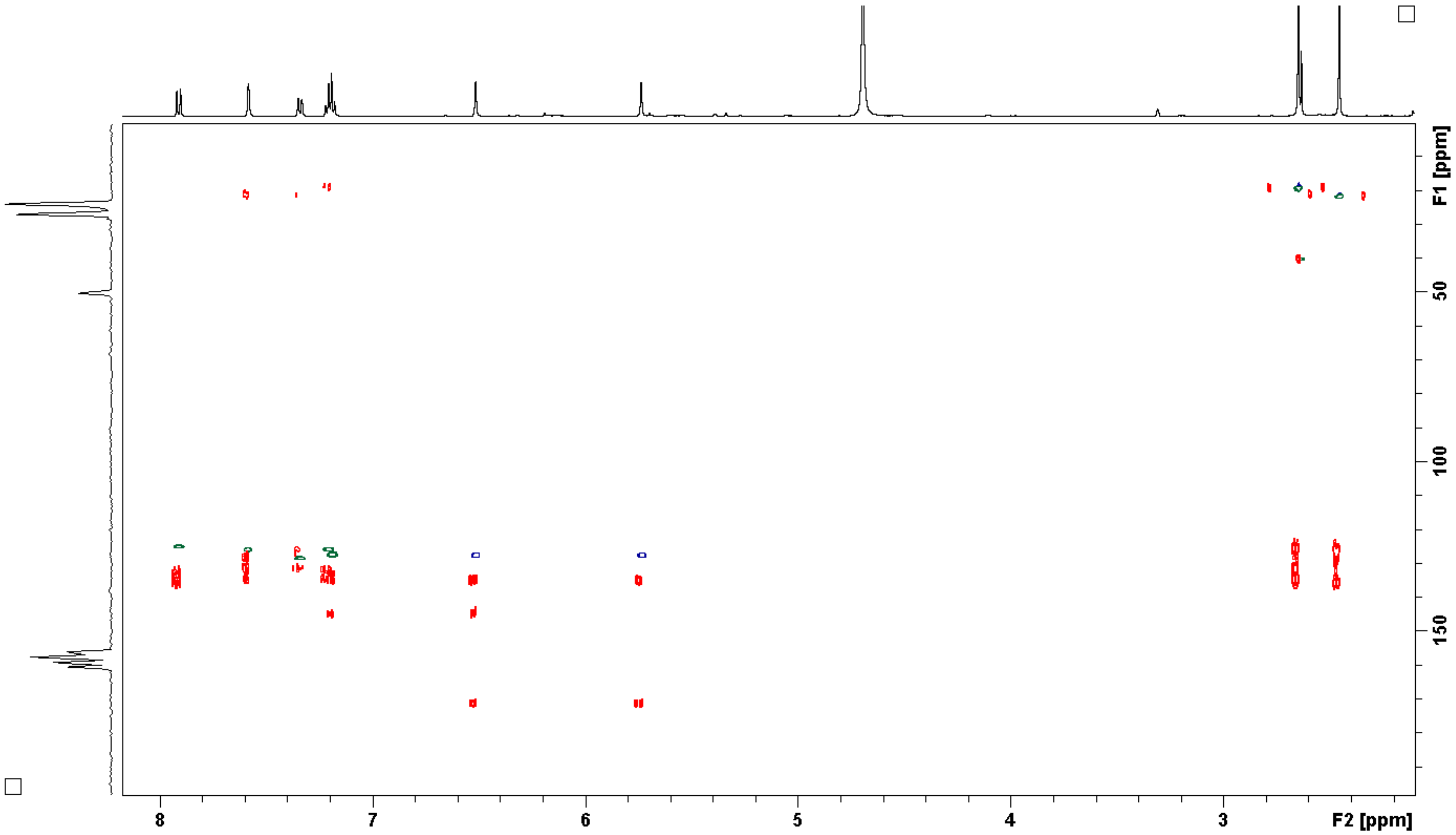

Figure S61: HSQC (green) and HMBC (red) overlaid spectra of compound **16** in CD_3OD.

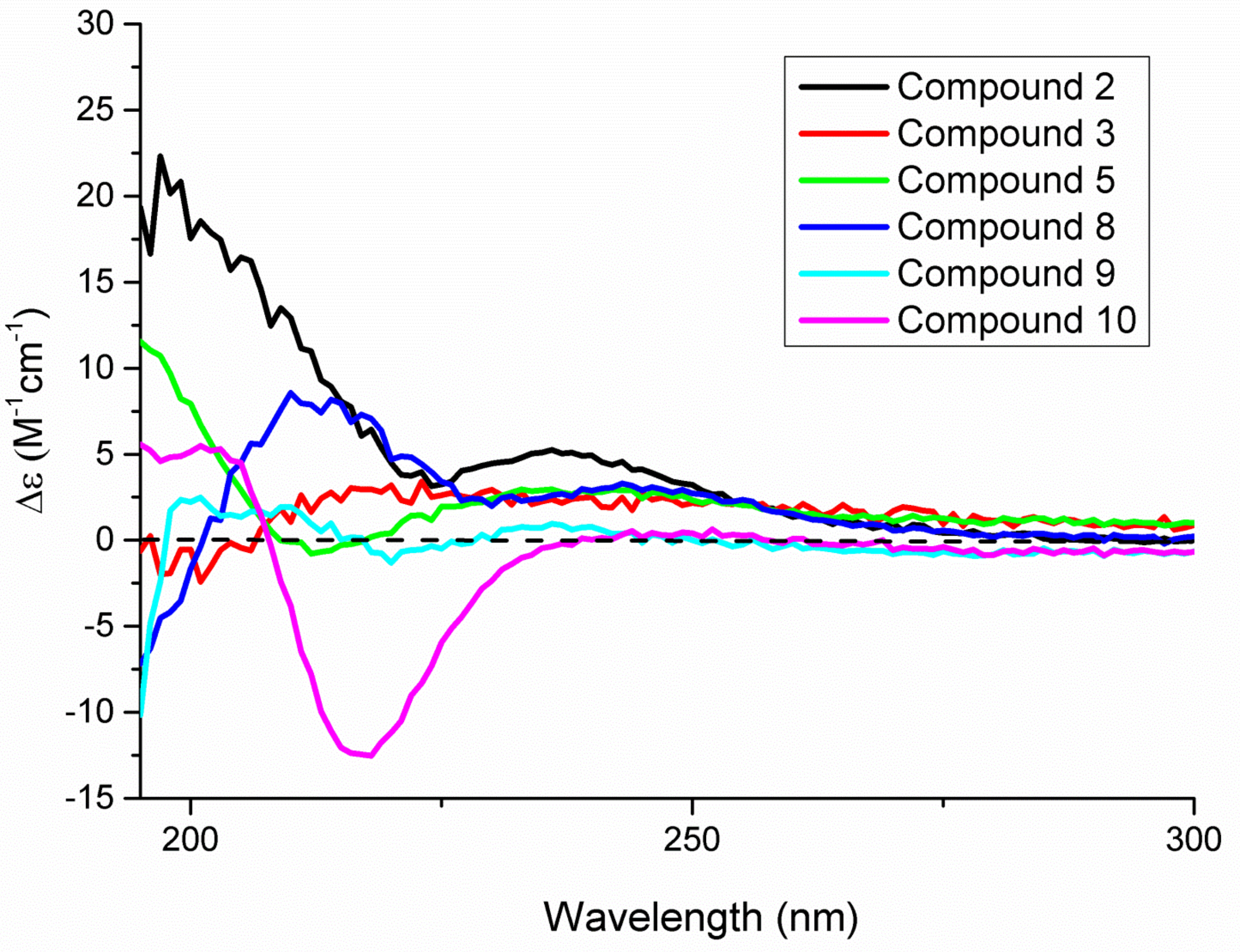

Figure S62: ECD spectra in MeOH of compounds **2**, **3**, **5**, **8**-**10**.

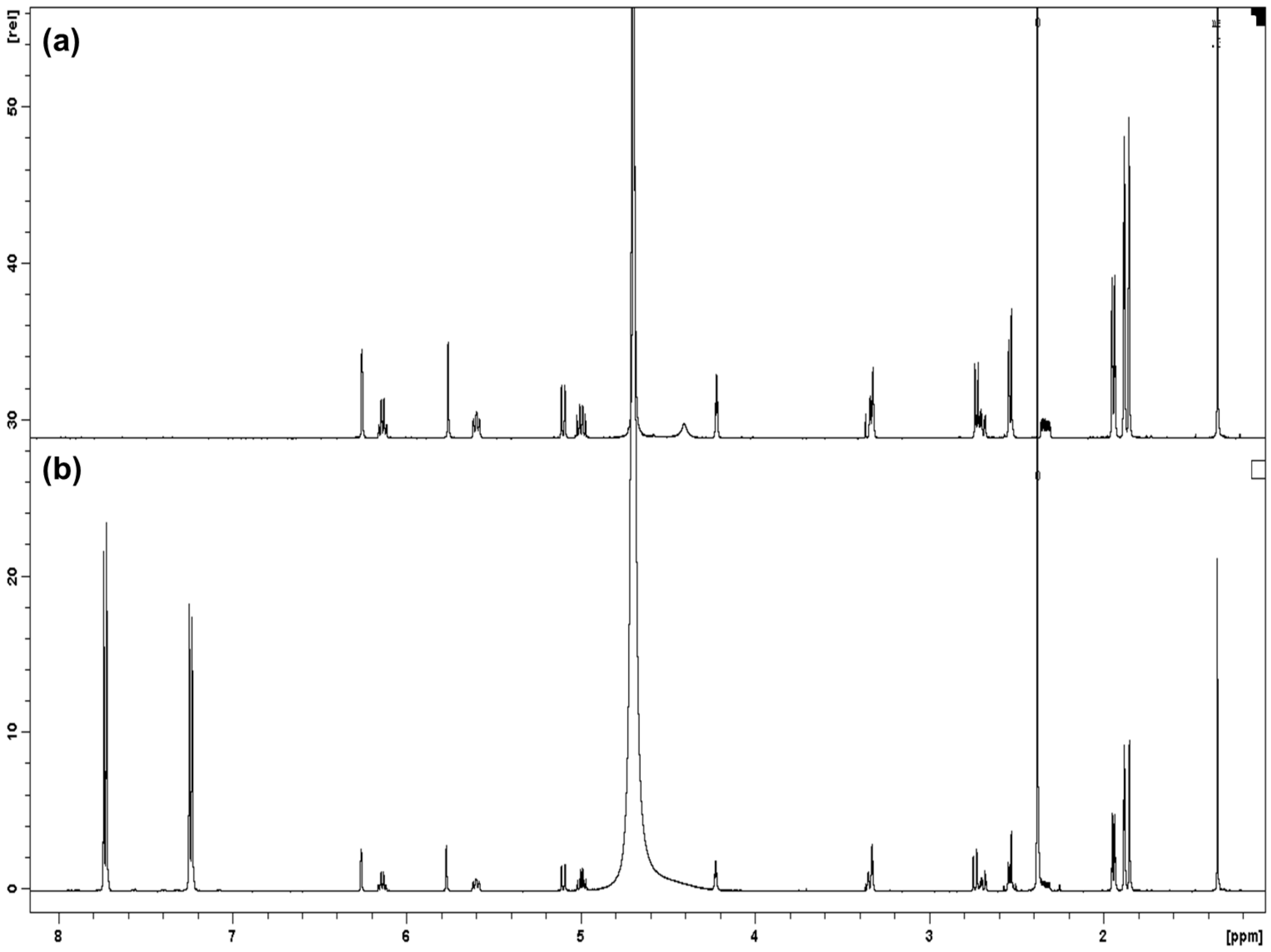

Figure S63: Overlay of ^{1}H NMR spectra of **3** (a) and of the reaction of **3** with *p*TSOH after 24 hours (b).
